# Covalently Labeled Fluorescent Exosomes for In Vitro and In Vivo Applications

**DOI:** 10.3390/biomedicines9010081

**Published:** 2021-01-16

**Authors:** María Isabel González, Mario González-Arjona, Ana Santos-Coquillat, Javier Vaquero, Elena Vázquez-Ogando, Antonio de Molina, Héctor Peinado, Manuel Desco, Beatriz Salinas

**Affiliations:** 1 Unidad de Medicina y Cirugía Experimental, Instituto de Investigación Sanitaria Gregorio Marañón (IiSGM), 28007 Madrid, Spain; migonzalez@hggm.es (M.I.G.); mgarjona@hggm.es (M.G.-A.); ascoquillat@hggm.es (A.S.-C.); 2 Unidad de Imagen Avanzada, Centro Nacional de Investigaciones Cardiovasculares (CNIC), 28029 Madrid, Spain; 3 HepatoGastro Lab, Servicio de Ap. Digestivo del HGU Gregorio Marañón, Instituto de Investigación Sanitaria Gregorio Marañón (IiSGM), 28007 Madrid, Spain; javiervaq@gmail.com (J.V.); elena.vazquez.ogando@hotmail.com (E.V.-O.); 4 Centro de Investigación Biomédica en Red en Enfermedades Hepáticas y Digestivas (CIBEREHD), 28029 Madrid, Spain; 5 Comparative Medicine Unit, Centro Nacional de Investigaciones Cardiovasculares (CNIC), 28029 Madrid, Spain; ademolina@cnic.es; 6 Microenvironment and Metastasis Laboratory, Department of Molecular Oncology, Spanish National Cancer Research Center (CNIO), 28029 Madrid, Spain; hpeinado@cnio.es; 7 Departamento de Bioingeniería e Ingeniería Aeroespacial, Universidad Carlos III de Madrid, 28911 Madrid, Spain; 8 Centro de Investigación Biomédica en Red de Salud Mental (CIBERSAM), 28029 Madrid, Spain

**Keywords:** extracellular vesicles, exosomes, vesicle labeling, optical imaging, fluorescence

## Abstract

The vertiginous increase in the use of extracellular vesicles and especially exosomes for therapeutic applications highlights the necessity of advanced techniques for gaining a deeper knowledge of their pharmacological properties. Herein, we report a novel chemical approach for the robust attachment of commercial fluorescent dyes to the exosome surface with covalent binding. The applicability of the methodology was tested on milk and cancer cell-derived exosomes (from U87 and B16F10 cancer cells). We demonstrated that fluorescent labeling did not modify the original physicochemical properties of exosomes. We tested this nanoprobe in cell cultures and healthy mice to validate its use for in vitro and in vivo applications. We confirmed that these fluorescently labeled exosomes could be successfully visualized with optical imaging.

## 1. Introduction

Exosomes are considered the smallest extracellular vesicles (50–150 nm) of endosomal origin [1]; their structures consist of a lipid bilayer membrane derived from the parent cell membrane [2]. Based on their physicochemical and structural properties, exosomes constitute a natural alternative to synthetic nanoparticles for developing novel drug delivery systems (DDS). In particular, they are especially suited to substituting for liposomes, due to their similar size and morphology [3,4]. In addition, the roles of exosomes in neural communication and pathological states, such as inflammation, tumorigenesis, and pre-metastatic niche formation [5,6], suggest that they could serve as a natural bio-targeting agent, with better accumulation properties, compared to synthetic nanoparticles [7]. Among the different types of exosomes extracted from natural sources, those derived from plants or food (including milk exosomes) have been widely tested as natural nanoplatforms in therapy and diagnostics due to their low toxicity and lack of immunogenicity [8,9]. Milk exosomes are particularly interesting because their production is suitable, scalable, and economic, and their strength and stability are superior to those of other exosomes [10,11,12]. These properties support the further development of exosomes as a DDS for translation into the clinical field.

Although exosomes have shown promise as a DDS in preclinical research [11,13], the transfer of these nanovesicles into the clinical field for therapeutic applications requires a better understanding of their in vivo behavior, particularly their biodistribution and organ/tissue targeting capabilities. Non-invasive imaging techniques can provide accurate in vivo information about their pharmacokinetics and biodistribution. Among the different techniques applied in biomedical research, fluorescence confocal imaging is currently highly popular because it provides detailed microscopic images easily, cost-effectively, and rapidly. Optical imaging also offers a highly versatile platform for non-invasive in vivo imaging due to its high sensitivity and the possibility of using a wide variety of probes, based on proteins, enzymes, or antibodies. In addition, optical imaging does not require ionizing radiation, unlike other techniques, such as nuclear (i.e., single photon emission computed tomography or positron emission tomography) or X-ray imaging. 

During the last few years, different approaches have been developed for the application of optical imaging to in vitro and in vivo evaluations of extracellular vesicles. These approaches vary from genetic engineering methods for incorporating bioluminescent proteins [14] or green fluorescent protein (GFP) [15] into the exosome structure, to directly labeling exosomes with fluorescent lipophilic dyes [16]. Due to the complexity of the genetic engineering approach, the use of fluorescent dyes has become the gold standard for visualizing exosomes. Currently, the integration of lipophilic fluorophores, such as dialkylcarbocyanine or PKH dyes, into the exosomal membrane represents the most widely used labeling methodology, due to its simplicity and low cost [17]. Nevertheless, some drawbacks limit the use of labeled exosomes, such as excessive exosome aggregation, the weak bond between the exosome and the fluorophore, which can cause the release of the dye from the exosome to non-targeted cells/tissues, and the instability of the resulting probes, which can result in false signals in optical imaging [17,18,19].

Here, we describe a direct, straightforward, covalent method for labeling exosomes with commercial fluorescent dyes, which enables in vitro and in vivo assessments with optical imaging techniques.

## 2. Experimental Section

### 2.1. Isolation of Milk Exosomes

Exosomes were isolated from commercial, fresh pasteurized, semi-skimmed goat milk (El Cantero de Letur, Albacete, Spain). Samples were centrifuged at 4 °C in 30-mL polycarbonate tubes, in an AVANTI J-30I centrifuge equipped with a Ja 30,50 Ti fixed-angle rotor (Beckman Coulter Instruments, Brea, CA, USA). Briefly, milk was centrifuged 10 min at 5000 G and treated with microbial rennet to remove fat globules and milk casein. The resultant supernatant was successively centrifuged for 10 min at 5000 G, 35 min at 13,000 G, and 15 min at 35,000 G to exclude contaminants, such as cell debris and large vesicles. Then, the milk whey was ultracentrifuged at 100,000 G for 70 min to precipitate the exosomes. The resultant pellet was washed three times with phosphate-buffered saline (1× PBS) and additionally purified in PD-10 size-exclusion columns (GE Healthcare Bio-Sciences, Uppsala, Sweden). The exosomes were concentrated to achieve the required volume by ultracentrifugation at 100,000 G for 90 min. The nanovesicles pellet was dispersed in 100 µL 1× PBS and stored at −20 °C until characterization and subsequent labeling. G values described on the isolation protocol refer to the average relative centrifugal force reached during the centrifugation step, after 5 min acceleration.

### 2.2. Cancer Cell Culture

Glioblastoma (U87) and mouse melanoma (B16F10) cell lines were kindly provided by the Cell Culture Unit of the Experimental Medicine and Surgery Unit at Hospital General Universitario Gregorio Marañón (Madrid, Spain) and Centro Nacional de Investigaciones Oncológicas (CNIO) Carlos III (Madrid, Spain), respectively. These cell lines were cultured in Dulbecco’s Modified Eagle Medium (DMEM; Gibco, ThermoFisher Scientific, Waltham, MA, USA) supplemented with 10% fetal bovine serum (FBS; exosome-free, Gibco, ThermoFisher Scientific, Waltham, MA, USA) and 1% L-glutamine, at 37 °C, 5% CO_2_, and controlled humidity in 95% air.

In order to remove extracellular vesicles present in serum, FBS was ultracentrifuged at 100,000 G and 4 °C for 18 h.

### 2.3. Isolation of Cancer Cell Line-Derived Exosomes

Exosomes were isolated from cancer cell lines U87 and B16F10 according to previous protocols [20]. Briefly, once cells reached approximately 80 % confluence, the cell culture was placed in polycarbonate tubes, and exosomes were harvested in four successive centrifugations at 4 °C, with an AVANTI J-30I centrifuge equipped with a Ja 30,50 Ti fixed-angle rotor (Beckman Coulter Instruments, Brea, CA, USA). The centrifugation steps were: first 10 min at 300 G, then 10 min at 2000 G, then 30 min at 10,000 G, and finally 70 min at 100,000 G. The nanovesicles pellet obtained was washed with 1× PBS and re-isolated at 100,000 G for 70 min. The final pellet of exosomes was dispersed in 200 µL of 1× PBS and stored at −20 °C until characterization and subsequent labeling. G values described on the isolation protocol refer to the average relative centrifugal force reached during the centrifugation step, after 5 min acceleration.

### 2.4. Protein Content Determination

The protein contents of unlabeled exosomes suspensions were quantified with the Bradford–Coomassie colorimetric assay. This assay employed a ready-to-use Coomassie staining reagent (Sigma-Aldrich, St. Louis, MO, USA), and the assay was performed according to the manufacturer’s instructions. Absorbance was measured with a Synergy™ HT Multi-Mode Microplate Reader (Biotek Instruments Inc., Winooski, VT, USA).

### 2.5. Fluorescent Labeling of Exosomes

Unless otherwise noted, all reagents were purchased from Sigma-Aldrich (St. Louis, MO, USA) and used without further purification. Commercial SCy 7.5 and BDP-FL succinimidyl ester fluorophores were acquired from Lumiprobe (Hannover, Germany).

Synthesis of SCy-Exo: a solution of 75 µg exosomes (from goat milk, B16F10, or U87) in 100 µL 1× PBS was mixed with 10 µL SCy 7.5 (17 mM) at pH = 8.5 (with NaHCO_3_ 0.1 M) and vortexed for 2 h at 4 °C. The product was purified with Exosome Spin Columns (Invitrogen, Carlsbad, CA, USA).

Synthesis of BDP-Exo: a solution of 75 µg exosomes (from goat milk) in 100 µL 1× PBS was mixed with 10 µL BDP-FL (25 mM) at pH= 8.5 (with NaHCO_3_ 0.1 M), and vortexed for 2 h at 4 °C. The product was purified with Exosome Spin Columns (Invitrogen, Carlsbad, CA, USA).

### 2.6. Physicochemical Characterization

The morphology and size of exosomes were evaluated at ICTS Centro Nacional de Microscopía Electrónica (Universidad Complutense de Madrid, Spain). They employed TEM (JEOL JEM-1010, JEOL USA Inc., Peabody, MA, USA), which operated at 100 kV. Exosomes were negatively stained with uranyl acetate on formvar carbon-coated copper grids at room temperature. The hydrodynamic sizes of control and labeled exosomes samples were measured with a Zetasizer Nano system (Malvern Panalytical, Malvern, UK). Concentrations (particles/mL) of exosomes in suspension as well as nanoparticles size were measured with a NanoSight NS500 (Malvern Panalytical, Malvern, UK), from Centro Nacional de Investigaciones Oncológicas (CNIO, Madrid, Spain), equipped with a high sensitivity sCMOS camera. Camera level and threshold were established in 11 and 10, respectively. Videos were recorded in static conditions and analyzed with NTA 3.1 Build software, and three replicate histograms were obtained for each sample

### 2.7. Flow Cytometry Analysis

Unlabeled and fluorescently labeled exosomes were evaluated with a GalliosTM 10 color Flow Cytometer (Beckman Coulter Instruments, Brea, CA, USA). A blue-light laser (488 nm; BODIPY 550 SP filter) was employed for analyzing exosomes labeled with BDP-FL. A red-light laser (635 nm; 725/20 filter) was employed for analyzing exosomes labeled with SCy 7.5. The maximum number of events was not restricted during the measurement. For discriminating exosomes from other non-specific events and background, a delimited region was placed in the flow cytometry dot-plot; this gate was adjusted to the expected size range of exosomes.

Flow cytometry data were analyzed with KaluzaTM Acquisition Software (version 1.5; Beckman Coulter, Brea, CA, USA). 

### 2.8. In Vitro Stability of Fluorescent Exosomes

In vitro stability was assessed by incubating 75 µg milk exosomes labeled with BDP-FL (BDP-MiExo) in 1× PBS for 72 h at 37 °C. At baseline (0 min), 12 h, 48 h, and 72 h, the samples were collected and measured with a HPLC system (Agilent 1200 series; Agilent Technologies, Santa Clara, CA, USA), equipped with a UV absorbance detector and a Yarra SEC-3000 column (300 × 7.8 mm; Phenomenex Inc., Torrance, CA, USA). An isocratic gradient of 1× PBS with a flow rate of 0.2 mL/min was employed with a UV measurement at 503 nm. In addition, HPLC chromatogram of non-labeled milk exosomes was performed to confirm purity of the sample using same gradient and column but with a UV measurement at 254 nm.

### 2.9. Isolation of Primary Mouse Hepatocytes

Animal experiments conducted this project complied with the ARRIVE guidelines and were in accordance with the EU Directive (2010/63/EU) for animal experiments. The HGUGM Animal Care and Use Committee approved all procedures. 

Hepatocytes were isolated from adult C57BL/6 mice (12–16 weeks old). These mice were genetically engineered to expressed enhanced green fluorescent protein EGFP on hepatocyte plasma membranes (mG) and Tomato-dye protein on the plasma membranes (mT) of other cell-types. Mice were generated with the Cre/LoxP system by crossing female mice that expressed Cre-recombinase under the control of the albumin promoter (AlbCre mice, B6.Cg-Speer6-ps1Tg(Alb-cre)21Mgn/J, stock# 3574, Jackson Lab, Farmington, CT, USA) and male mice that expressed the double-fluorescent Cre reporter (mT/mG mice; B6.129(Cg)-Gt(ROSA)26Sortm4(ACTB-tdTomato,-EGFP)Luo/J, stock# 7676, Jackson Lab, Farmington, CT, USA) [21].

Primary mouse hepatocytes were isolated with a two-step protocol for hepatic perfusion from the inferior cava vein, according to previously published protocols [22], with some modifications. Briefly, the liver was first perfused with Hank’s Balanced Salt Solution (without calcium, magnesium, or phenol-red; Gibco, ThermoFisher Scientific, Waltham, MA, USA), containing 10 mM HEPES (pH 7.4), 0.2 mM EGTA, and 10 U/mL heparin. This was followed by perfusion with Williams E Medium (Gibco, ThermoFisher Scientific, Waltham, MA, USA), containing 10 mM HEPES (pH 7.4) and 0.4 mg/mL collagenase type IV from Clostridium histolyticum (Sigma-Aldrich, St. Louis, MO, USA). After filtering the digested liver through a 100 µm-pore cell strainer, cells were resuspended in attachment media (DMEM:F12 with 10% FBS supplemented with 5 mM sodium pyruvate, 2 mM L-glutamine, 0.05% NaHCO_3_, 20 mM HEPES, 0.12% glucose, 0.02% BSA, 100 U/mL penicillin, and 100 μg/mL streptomycin). Next, the mixture was centrifuged at 50 G, and purified with density gradient centrifugation in an isotonic solution of Percoll (GE Healthcare Bio-Sciences AB, Uppsala, Sweden). After a wash in attachment media with a 50 G centrifugation, cell viability was checked with Trypan blue.

### 2.10. Confocal Studies of in Vitro Uptake 

A 24-well cell culture plate (Corning, NY, USA) was pretreated with a 1:1000 solution of collagen type (I), and one round glass coverslip was added per well. Isolated primary mouse hepatocytes were seeded at a density of 20,000 cells per well and cultured overnight in attachment medium under controlled humidity conditions, in 95% air and 5% CO_2_, at 37 °C. Next, cells were incubated for 30 min, 60 min, 120 min, 240 min, and 24 h with 5 µg/mL (2.5 µg/well) or 0.5 µg/mL (0.25 µg/well) milk exosomes labeled with SCy 7.5 (SCy-MiExo). Next, the hepatocytes were washed twice with PBS and fixed with 4% paraformaldehyde (PFA) for 5 min. After removing the PFA and washing with PBS, the glass coverslips were placed on confocal slides and mounted with DAKO mounting medium (Agilent Technologies, Santa Clara, CA, USA), which contained DAPI fluorescent dye, for nuclei staining.

NIR fluorescent signals from SCy-MiExo were captured with a Leica-SPE microscope (Leica Microsystems Inc., Buffalo Grove, IL, USA) equipped with a 635 nm laser. Images were processed with Fiji ImageJ Software (U. S. National Institutes of Health, Bethesda, MD, USA). Exosome uptake was assessed by measuring the fluorescent signals in regions of interest (ROIs) in the perinuclear region of the cell.

### 2.11. In Vivo Optical Imaging

Fluorescent milk exosomes (SCy-MiExo) were administered to healthy CD1 female mice (*n* = 3 animals, weight 20–25 g). Mice were previously shaved in the abdominal region, and SCy-MiExo solutions were intravenously injected through the lateral tail vein (25 μg, 150 μL in 1X PBS). The in vivo biodistribution of SCy-MiExo was evaluated with an in vivo imaging system (IVIS Spectrum System; PerkinElmer Inc., Waltham, MA, USA) at 1 h, 4 h, and 24 h after injection. During image acquisition, mice were maintained under 1.5–2% isofluorane. Fluorescent detection was performed with an indocyanine green filter set (excitation: 710/760 nm, emission: 810/875 nm), and the exposure time was under 0.5 s. To evaluate the natural behavior of the free SCy 7.5 dye, we conducted a control study with healthy CD1 female mice (*n* = 2, weight 20–25 g). Briefly, mice received an intravenous injection of commercial SCy 7.5 (17 mM, 150 μL, in 1× PBS). The imaging conditions were the same as those described above for SCy-MiExo imaging, except the exposure time was reduced to 0.2 s. All animals were sacrificed at 24 h post-injection, and the liver, spleen, kidneys, intestines, heart, lungs, brain, and skin were harvested and imaged with the same device. 

Fluorescence images were quantified with Living Image Software provided with the IVIS Spectrum System (PerkinElmer Inc., Waltham, MA, USA). We measured the average radiant efficiency of the SCy-MiExo fluorescent signal from ROIs in all the excised organs. The average radiant efficiency was defined as the number of photons (p) that left a square centimeter of tissue per second and radiated into a solid angle of one steradian (sr). The resulting values, expressed in (p/s/cm^2^/sr)/(μW/cm^2^), were normalized to the integration time, binning, f/stop, field of view, illumination intensity, and the ROI area, to ensure comparability between measurements. 

### 2.12. Histological Analysis of Hepatic Tissue

At 24 h after the in vivo imaging, mice inoculated with SCy-MiExo, (intravenous administration, *n* = 3) were sacrificed, and livers were collected. Tissues were prepared for both confocal microscopy and histopathological analysis. Briefly, organs were fixed in 4% PFA for 24 h, transferred to 70% ethanol, and embedded in paraffin for routine hematoxylin and eosin histochemical staining. 

For confocal imaging, liver tissues were fixed in 4% PFA for 24 h, dehydrated in a sucrose solution, and embedded in optimal cutting temperature compound (OCT) for cutting into 10-µm sections. Images were acquired with a Leica SP5 microscope (Leica Microsystems Inc., Buffalo Grove, IL, USA) equipped with a 633-nm excitation laser, combined with a 10× dry-objective and a 20× oil-immersion objective. Image processing was carried out with LAS-AF 2.7.3. build 9723 Software incorporated into the microscope and with Fiji ImageJ Software (U. S. National Institutes of Health, Bethesda, MD, USA).

### 2.13. Data Analysis

Data processing and graph construction were performed with Prism 6.0c software (GraphPad Software, La Jolla, CA, USA). Data are expressed as the mean ± standard deviation.

Statistical analysis of the in vitro uptake was performed using one-way ANOVA and Tukey’s multiple comparison test with Prism 8.4.3 software (GraphPad Software, La Jolla, CA, USA), after checking for normality. Significant differences stand for: * (*p* ≤ 0.05), ** (*p* ≤ 0.01), *** (*p* ≤ 0.001), **** (*p* ≤ 0.0001).

## 3. Results

The optimized labeling strategy we developed was successful and robust, based on a complete physicochemical characterization of fluorescent nanovesicles derived from milk and cancer cell lines. These novel nanoprobes displayed in vitro and in vivo stability. The applicability of these probes was evaluated by assessing uptake in primary hepatocytes and biodistribution in healthy mice, with confocal microscopy and in vivo optical imaging. 

### 3.1. Characterization of Fluorescent Milk Exosomes

**Figure 1:**
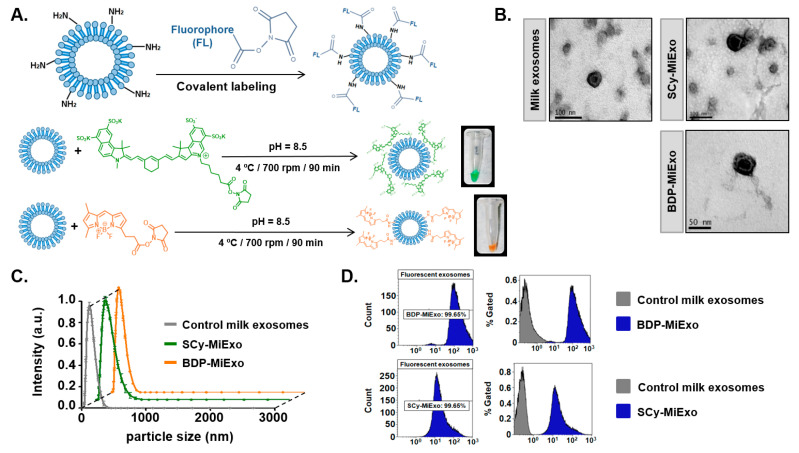
Optical labeling and physicochemical characterization of control and fluorescence-labeled milk exosomes (MiExo). (**A**) Chemical strategies for labeling exosomes with Bodipy FL (BDP-FL, orange) and sulfo-cyanine 7.5 (SCy 7.5, green), and the resulting fluorescently labeled exosomes in dilution fluids. (**B**) Transmission electron microscope images for morphological evaluations of exosomes. (**C**) Size distributions of nanovesicles, evaluated with dynamic light scattering. Data are expressed as the mean ± standard deviation; (**D**) Flow cytometry results show the abundances of control exosomes (grey) and fluorescent nano-probes (blue). SCy MiExo: SCy 7.5-labeled milk exosomes; BDP-MiExo: BDP-FL-labeled milk exosomes.

Purified milk exosomes (MiExo) labeled with bodipy FL (BDP-FL) and sulfo-cyanine 7.5 (SCy 7.5) showed bright orange and green colors, respectively (Figure 1A). To determine the successful attachment of the dye to the exosomes, we compared unlabeled exosomes to purified fluorescent exosomes with flow cytometry. For unlabeled milk exosomes, 0.44% of events emitted a fluorescent signal at 503 nm and 0.05% of events emitted a fluorescent signal at 778 nm. For BDP-MiExo and SCy-MiExo, we observed fluorescent signals in 99.56% (at 503 nm) and 99.98% (at 778 nm) of the events, respectively (Figure 1D). 

Transmission electron microscopy (TEM) analyses of fluorescently labeled exosomes showed ‘cup-shaped’ structures typical of extracellular vesicles. The core size was similar to that of control unlabeled nanovesicles (Figure 1B), with no morphological alterations. A dynamic light scattering (DLS) analysis showed that the hydrodynamic sizes were 135.31 ± 13.10 nm for BDP-MiExo and 135.33 ± 9.43 nm for SCy-MiExo (Figure 1C). With both fluorescent labels, the exosomes slightly increased in hydrodynamic size, compared to the control exosomes (126.12 ± 2.94 nm). Size measurements obtained by Nanoparticle tracking analysis (NTA) for fluorescent labeled exosomes registered quite similar values for SCy-MiExo (134.30 ± 8.50 nm) and BDP-MiExo (140.40 ± 6.30 nm). This technique also reported concentrations of 1.02 × 10^9^ ± 6.20 × 10^7^ particles/mL for BDP-MiExo and 1.65 × 10^9^ ± 1.18 × 10^8^ particles/ml for SCy-MiExo, slightly lower values than those measured for the unlabeled exosome solution (5.62 × 10^9^ ± 2.29 × 10^8^ particles/mL). 

### 3.2. Characterization of Fluorescent Cancer Cell Line-Derived Exosomes

**Figure 2:**
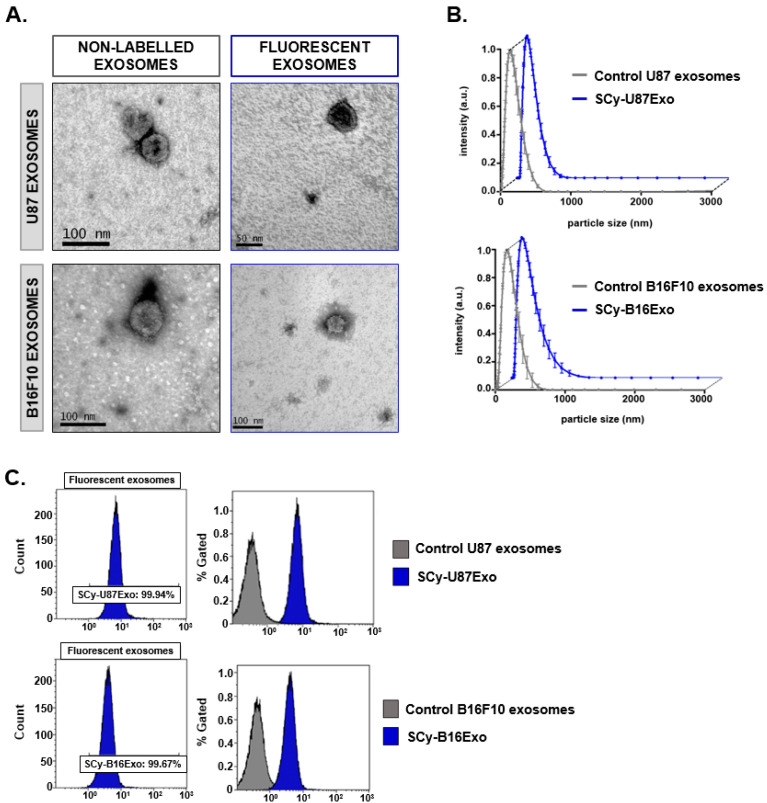
Physicochemical characterization of control and fluorescence-labeled cancer cell line-derived exosomes. (**A**) Transmission electron microscope images show the nanovesicle morphology. (**B**) Size distributions of exosomes produced with dynamic light scattering. Data are expressed as the mean ± standard deviation. (**C**) Flow cytometry results show abundances of control exosomes and the fluorescent nano-probes. SCy: sulfo-cyanine 7.5; U87Exo: exosomes derived from U87 glioblastoma cells; B16Exo: exosomes derived from B16F10 mouse melanoma cells.

Similar to milk exosomes, U87 and B16F10 exosome solutions exhibited a strong green color after labeling with SCy 7.5. Flow cytometry measurements in the size range of exosomes showed fluorescence signal emissions in 99.94% of events with SCy-labeled U87 exosomes (SCy-U87Exo) and 99.67% of events with SCy-labeled B16 exosomes (SCy-B16Exo, Figure 2C). In unlabeled exosomes derived from U87 cells (U87Exo) and B16F10 cells (B16Exo), 3.03% and 4.95% of events, respectively, exhibited fluorescent signals at 778 nm.

Physicochemical characterizations of control and labeled exosomes showed that labeling had minor effects on the morphological properties of these cancer cell line-derived exosomes. TEM imaging and DLS confirmed that SCy-U87Exo samples preserved the morphology and size of the non-labeled controls (Figure 2A); the hydrodynamic sizes were 132.00 ± 10.00 nm for control U87Exo and 135.32 ± 11.17 nm for the SCy-U87Exo (Figure 2B). Labeled U87Exo also exhibited a similar core size of 123.20 ± 7.00 nm after NTA analysis. However, the number of nanoparticles measured with NTA was slightly lower for SCy-U87Exo, after the chemical reaction (2.25 × 10^9^ ± 2.05 × 10^8^ particles/mL), compared to control U87Exo (3.35 × 10^9^ ± 1.42 × 10^8^ particles/mL).

TEM analyses confirmed that SCy-B16Exo showed typical exosome morphology. However, DLS showed a slight reduction in size distribution, compared to the controls (141.77 ± 1.00 for SCy-B16Exo nm vs. 177.00 ± 13.00 for control B16Exo; Figure 2B). The NTA analysis confirmed the size reduction of the nanovesicles (139.3 ± 10.2 nm) and revealed a slightly higher number of particles in the suspension of fluorescent SCy-B16Exo compared to unlabeled B16Exo (5.91 × 10^8^ ± 4.82 × 10^7^ particles/mL vs. 9.59 × 10^8^ ± 1.40 × 10^8^ particles/mL).

### 3.3. In Vitro Stability of Fluorescent Exosomes

**Figure 3:**
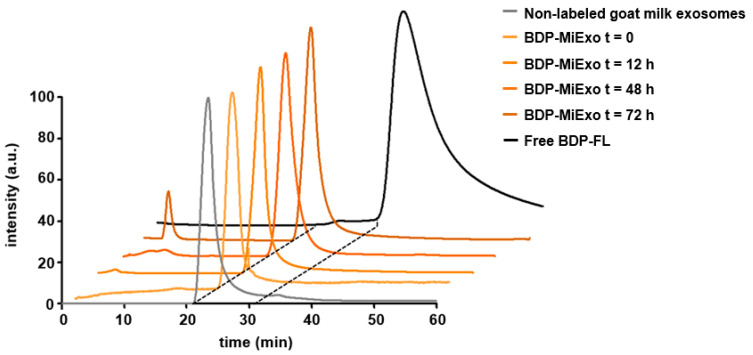
In vitro stability of Bodipy FL-labeled milk exosomes (BDP-MiExo) over time, evaluated with high performance liquid chromatography.

The temporal stability of exosome labeling was confirmed in a longitudinal in vitro study with high performance liquid chromatography (HPLC). Fluorescent chromatograms of BDP-MiExo showed a single peak at 25.2 min (Figure 3), which corresponded to the retention time of pure unlabeled milk exosomes. No peak was registered at 38.3 min, which was the characteristic retention time for free BDP-FL. 

A small peak was detected at a retention time of 3.8 min after 72 h of incubation, corresponding to events higher than exosomes, probably due to their aggregation at long time points.

It was not possible to assess the stability of exosomes labeled with SCy 7.5 (at 778 nm) due to the limitations of the HPLC equipment. The upper limit of the absorbance detector was 600 nm. 

### 3.4. In Vitro Study of SCy-MiExo

**Figure 4:**
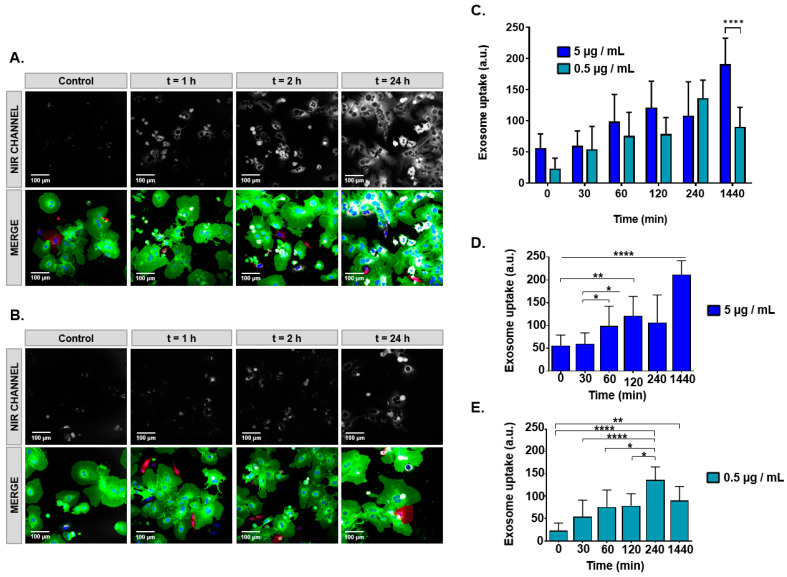
Confocal microscope imaging for assessing the uptake of sulfo-cyanine 7.5-labeled milk exosomes (SCy-MiExo) by hepatocytes. (**A**,**B**) Near infrared (NIR, top) and fluorescence images (bottom) taken over time as hepatocytes internalized: (**A**) 5 µg/mL and (**B**); 0.5 µg/mL of SCy-MiExo; (**C**) Quantification of the exosome uptake at different SCy-MiExo concentrations, analyzed in regions of interest. Values were statistically significant between both concentration at 24 h; **** (*p* ≤ 0.0001). Data are expressed as the mean ± standard deviation. (**D**) Statistical analysis for the dose of 5 µg/mL: all values were significant compare to 24 h; * (*p* ≤ 0.05), ** (*p* ≤ 0.01), *** (*p* ≤ 0.001), **** (*p* ≤ 0.0001). (**E**) Statistical analysis for the dose of 0.5 µg/mL; * (*p* ≤ 0.05), ** (*p* ≤ 0.01), *** (*p* ≤ 0.001), **** (*p* ≤ 0.0001).

The ability of cells to internalize these natural nanovesicles was assessed with confocal imaging. Hepatocytes were exposed to both high (5 µg/mL) and low (0.5 µg/mL) doses of SCy-MiExo, and fluorescent signals were observed in the cytoplasm, even at short time points (1 h). The exosomes were distributed throughout the cytoplasm, especially in the perinuclear region (Figure 4A,B), and uptake was dose-dependent (Figure 4C). The time to the peak signal depended on the dose of exosomes added; in the near infra-red (NIR) channel, the brightest signals were observed after 24 h of incubation at the high dose, and after 4 h for the low dose (Figure 4C). Values registered at the end point of the experiment (24 h) were statistically different if both dose. Moreover, exosome uptake registered at 24 h in case of 5 µg/ml is highly significant compare to the other time points for the same concentration (Figure 4D).

### 3.5. In Vivo and Ex Vivo Studies with SCy-MiExo

**Figure 5:**
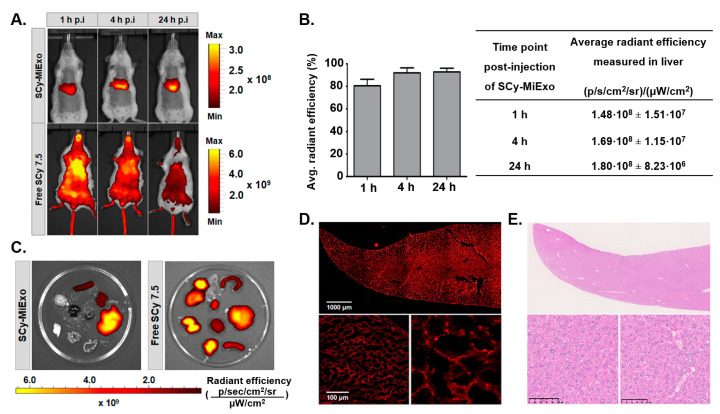
In vivo and ex vivo studies with SCy-MiExo: (**A**) In vivo optical imaging of sulfo-cyanine 7.5-labeled milk exosomes (SCy-MiExo, top) and free SCy 7.5 (bottom) in healthy mice. (**B**) The time course of average radiant efficiency measured in vivo in livers of mice treated with SCy-MiExo. The data are expressed as the % (graph) and in units of p/s/cm^2^/sr)/(μW/cm^2^) (table), with the mean ± standard deviation. (**C**) Ex vivo biodistribution of SCy-MiExo (left) and free SCy 7.5 (right) in excised organs. (**D**) Confocal images of liver sections from mice treated with SCy-MiExo. Right down image presents a zoom of left down image. (**E**) Hematoxylin and Eosin (H&E) histological images of liver sections from mice treated with SCy-MiExo.

In vivo whole-body optical imaging showed that SCy-MiExo was mainly taken up in liver tissues (Figure 5A). The signal intensity in this organ increased over time, with maximum values at 24 h (Figure 5B). The biodistribution examined ex vivo, in the organs excised at 24 h, confirmed these results. Among all the organs studied, exosome uptake was highest in the liver (Table 1). We also detected fluorescent signals in the spleen and kidneys, but no significant uptake was detected in the other harvested organs (Figure 5C). 

In contrast, in vivo imaging of the free dye showed a homogenous distribution throughout the entire body, and drastic reduction in the fluorescent intensity was observed after 4 h (Figure 5A). All of the excised organs evaluated ex vivo after 24 h post-injection showed the free fluorescent signal (Table 1). The biodistribution profile of free SCy 7.5 was absolutely different from that of SCy-MiExo (Figure 5C).

A histological study of the liver tissue (Figure 5D) showed high uptake and a homogeneous distribution of SCy-MiExo. Hematoxylin and eosin stained liver samples showed no significant alterations in liver tissue structure. We only detected some minor alterations related to the exposure to nanometric ‘foreign bodies’, such as large accumulations of Kupffer cells [23] or some vacuolization in hepatocytes, compared to the normal compartmentation of glycogen inside these hepatic cells (Figure 5E).

## 4. Discussion

Traditionally, the methodology employed in fluorescent exosome labeling for histological and cellular assessments has relied on the passive integration of lipophilic dyes into the exosome membrane [17,24]. However, passive integration implies a weak bond between the exosome structure and the dye, which could lead to the detachment and release of the fluorophore; the free fluorophore can produce false positives or increase the background signal [17]. To resolve these limitations, the present study described a straightforward methodology that ensured strong dye attachment to the exosome by creating covalent bonds between the ester groups of commercial fluorophores and the amine groups present in nanovesicles. This chemical methodology relied on the disposition of free amine groups from transmembrane proteins in the exosome membrane, and it was previously exploited in other protocols for exosome surface engineering with bio-orthogonal functional groups and radioisotopes [25,26]. To our knowledge, this study was the first to optimize this approach for labeling exosomes and to facilitate optical characterizations. 

To demonstrate the versatility of our approach, we tested two widely different commercial fluorophores with different hydrophobicities, wavelengths, and chemical structures: BDP-FL and SCy 7.5. Moreover, we tested the approach in different types of exosomes, derived from goat milk and U87 and B16F10 cancer cells. The BDP-FL fluorophore (503 nm excitation) is typically used in preclinical studies of tissues and cells [27,28]. The SCy 7.5 fluorophore (778 nm excitation) is an ideal tool for in vivo applications; it can be detected in the NIR range, which provides relatively deep penetration and avoids the inconvenience of autofluorescence [29]. Moreover, SCy 7.5 is highly soluble in aqueous media, which provides an advantage over the more commonly used NHS ester dyes. For example, Cyanine 7 requires organic solvents for the labeling reaction, which might be toxic or otherwise harm these natural nanovesicles. 

Exosomes isolated from goat milk were successfully labeled with both fluorophores. Visually, the intense colors emitted by the purified samples clearly showed that the dyes were incorporated into the exosome structures. Incorporation of the dye was confirmed and quantified with flow cytometry, which showed that 99% of the exosome population was labeled. Although it is well known that these natural nanoparticles are sensitive to alterations in media conditions, our physicochemical characterization of labeled exosomes showed that the morphological structure of these nanoparticles was not significantly altered. We only observed a slight increase in the hydrodynamic size of fluorescently labeled exosomes compared to controls. This change in size was most likely due to the presence of the fluorophore on the exosome surface. Nevertheless, TEM analyses confirmed that the vesicles were round, with an intact lipid bilayer membrane. The slight reduction in the number of nanovesicles measured with NTA after the labeling reaction could be attributed to some sample loss during the purification step. 

We also tested our procedure with two different cancer cell line-derived exosomes (U87 and B16F10). We employed SCy 7.5 for further in vivo experiments due to its convenient physicochemical properties (high solubility and appropriate emission wavelength). Moreover, BDP-Fl fluorophore requires organic media such as dimethyl sulfoxide (DMSO), which could compromise the integrity of the cancer cell line-derived exosomes, less robust than milk exosomes in degrading conditions [12,30].

Similar to the milk exosomes, both glioblastoma (SCy-U87Exo) and melanoma (SCy-B16Exo) cell-derived exosomes showed an intense green color after dye binding. Moreover, both samples were efficiently labeled, based on flow cytometry measurements. Similar to the fluorescent milk exosomes, the physicochemical characterization of SCy-U87 exosomes showed that both size and morphology were preserved after the labeling reaction. The slight reduction in the number of nanovesicles detected with NTA could also be explained by a small loss of nanovesicles during the purification step. In contrast, although SCy-B16Exo samples showed preserved morphological characteristics, the hydrodynamic size of these fluorescent exosomes was unexpectedly smaller than the size of unlabeled nanovesicles, unlike our findings with the milk and U87 exosomes. Previous studies showed that some exosomes tended to remain in an aggregated state after they are dispersed and stored in a saline solution, due to their surface composition [31]. NTA and DLS analyses could not distinguish between one big particle and two small, aggregated nanovesicles. Thus, exosome surface labeling with fluorophores could reduce their tendency to aggregate, which might lead to higher particle counts and more accurate size distribution measurements. 

Large extracellular vesicles, which typically present electron-dense appearance and irregular shape in TEM images [32] were not located in any of the samples analyzed, supporting the exosome nature of the isolated nanovesicles from both milk and cancer cell lines. In addition, DLS and NTA registered homogeneous populations of nanovesicles in the size range of exosomes (30–150 nm) [33].

One of the key points in the development of our chemical approach was the strong attachment of the dye to the nanoparticle, which prevented detachment and release of the free fluorophore. The high stability of labeled exosomes over time was demonstrated with an in vitro longitudinal HPLC study. The absence of a secondary peak at 38.3 min, associated with the free fluorophore, confirmed the absence of free BDP-FL in the sample at all time points, even after 3 days. This finding highlighted the strength of the covalent link established between the fluorophore and the vesicle structure. A small peak at 3.8 min was only detected after long incubation times (72 h). That peak suggested that the milk nanovesicles were beginning to aggregate because our size exclusion columns allowed the larger molecules and nanoparticles to pass through first, and the smaller particles passed through later. 

For in vitro and in vivo assessments of these novel optical nanoprobes, we decided to employ the SCy-MiExo vesicles because the milk exosomes showed promise as a DDS [11,34]. We performed in vitro evaluations of SCy-MiExo internalization into primary hepatocytes with confocal imaging. The reason for choosing this cell line was that it is well-known that the liver accumulates nanoparticles of 100–200 nm. We merged the green fluorescent images from the cells with the NIR images of the exosomes and we observed fluorescent exosome uptake even at short time points (30 min), which suggested rapid incorporation, as reported in previous studies of exosomes in tumors and inflammatory cells [35,36]. At all the studied time points, cytoplasmic accumulation was detected in the perinuclear area, similar to findings previously reported for synthetic liposomes of similar size [37]. This result indicated that these nanoparticles showed promise for use as drug delivery platforms. The cellular uptake time curve showed maximal uptake at different time points depending on the exosome dose. These time and dose effects were previously described for milk exosomes incubated with cancer cells [11]. In our case, a low dose (0.5 µg/mL) of SCy-MiExo showed the highest signal at 4 h, then it declined after 24 h. This pattern might reflect the fact that the total amount of exosomes was incorporated inside the hepatocytes after 4 h, then the exosomes were digested by the cells, which led to a decline in the fluorescent signal intensity at the latest time point (24 h). This behavior was previously observed with other cell-derived exosomes, which pointed to the possibility that nanovesicles were degraded after being engulfed by macrophages [35]. The high dose (5 µg/mL) of SCy-MiExo was clearly taken up at initial points (1 and 4 h), but the brightest signal intensity was recorded at 24 h. This result could be due to the high availability of exosomes; thus, after 4 h, exosomes remained available in the cell culture for internalization by hepatocytes at later time points. 

The in vivo behavior of fluorescent SCy-MiExo was evaluated with optical imaging. The SCy dye was selected because it showed high hydrophilicity and high intensity emission in the NIR range, which is far from the autofluorescence range of natural biomolecules, such as hemoglobin [38]. We performed longitudinal in vivo imaging of the fluorescent nanovesicles in healthy mice to evaluate their biodistribution over time. Liver accumulation was observed at 1 h post-injection, which confirmed that these nanovesicles were cleared rapidly, primarily through hepatobiliary metabolism. This in vivo biodistribution and mainly hepatobiliary metabolism was consistent with the pharmacokinetic profile observed in previous studies for small nanoparticles of similar size and shape, like synthetic liposomes or metallic nanoparticles [39]. As a control, we evaluated the in vivo biodistribution of the free dye. We observed high uptake of free SCy in the lungs, and a rapid decline in the fluorescent signal; this decline was linked to rapid renal excretion, evidenced by the high accumulation of free dye in the kidneys at 24 h post-injection. This behavior was typical of free fluorophores [40] and other small molecules [41]. This clear difference in the biodistribution profiles of free SCy 7.5 and fluorescent exosomes indicated that the nanovesicle labeling was robust and stable. A previous study from our group showed that the administration route affected the biodistribution of goat milk exosomes [42]. In that study, nuclear imaging showed the same in vivo biodistribution profile as that obtained in the present study with fluorescent SCy-MiExo, which implied that our approach did not modify the pharmacokinetic properties of the exosomes. Finally, our ex vivo imaging study of the organs confirmed that exosomes mainly accumulated in the liver, spleen, and kidneys, consistent with findings from previous studies that employed exogenous exosomes [17].

In conclusion, we developed a technique for readily determining the natural biodistribution of fluorescently labeled vesicles over time, without requiring the sacrifice of animals. We showed that milk exosomes were mainly taken up and retained in the liver and spleen, due to the phagocytic activity of Kupffer macrophages in these organs [43]. On the other hand, our histological evaluation confirmed that SCy-MiExo accumulation did not cause tissue damage or alterations in liver tissue structure. These observations opened up a novel avenue of approach for deep research on the potential role of milk exosomes in liver-targeting therapies. 

This study presented a straightforward methodology for the covalent labeling of exosomes with commercial fluorophores. The reproducibility and robustness of the technique were assessed with two different dyes and several types of exosomes (derived from milk and two types of cancer cells). In all cases, we demonstrated the success of this chemical approach and the preservation of the physicochemical properties of the nanovesicles. 

The applicability of this approach was validated with different optical imaging techniques. On the cellular scale, we demonstrated with confocal imaging that covalently labeled exosomes were internalized in hepatocytes. The results confirmed that labeled exosomes rapidly accumulated around the nucleus in a dose-dependent manner. Fluorescent exosomes were also successfully visualized in vivo, where they showed behavior similar to that observed for other synthetic nanoparticles, such as liposomes. These results suggested that our approach could be used for non-invasive in vivo applications, and that fluorescently labeled natural nanovesicles could serve as a substitute for synthetic nanoparticles in therapeutic applications.

## Figures and Tables

**Table 1:** Ex vivo average radiant efficiencies (p/s/cm^2^/sr)/(μW/cm^2^) of sulfo-cyanine 7.5-labeled milk exosomes (SCy-MiExo) and free SCy 7.5 measured in organs excised at 24 h post-injection. Data are expressed as the mean ± standard deviation.

Organ	SCy-MiExo: Ex Vivo Average Radiant Efficiency	Free SCy 7.5: Ex Vivo Average Radiant Efficiency
Liver	3.36 × 10^8^ ± 5.84 × 10^6^	9.66 × 10^8^ ± 2.20 × 10^7^
Kidneys	1.12 × 10^8^ ± 4.72 × 10^6^	9.47 × 10^8^ ± 7.72 × 10^7^
Small intestine	-	8.62 × 10^8^ ± 3.50 × 10^8^
Skin	-	7.56 × 10^8^ ± 3.53 × 10^8^
Lungs	-	5.35 × 10^8^ ± 3.69 × 10^8^
Intestines	-	4.87 × 10^8^ ± 2.03 × 10^8^
Heart	-	4.45 × 10^8^ ± 1.13 × 10^7^
Spleen	9.94 × 10^7^ ± 4.63 × 10^6^	3.48 × 10^8^ ± 2.55 × 10^7^
Brain	-	3.29 × 10^8^ ± 1.05 × 10^7^

## Data Availability

Data is contained within the article as supplementary material: Supplementary Table S1: Experimental data from Figure 1C. Physicochemical characterization of control and fluorescently labeled milk exosomes. Size distributions established with DLS; Supplementary Table S2: Experimental data from Figure 2B. Physicochemical characterization of control and fluorescently labeled cancer cell line-derived exosomes. Size distributions established with DLS; Supplementary Table S3: Experimental data from Figure 3. In vitro stability of BDP-MiExo evaluated with HPLC; Supplementary Table S4: Experimental data from Figure 4C. Exosome uptake analysis in regions of interest (dose: 5 µg/mL SCy-MiExo); Supplementary Table S5: Experimental data from Figure 4C. Exosome uptake analysis in regions of interest (dose: 0.5 µg/mL SCy-MiExo); Supplementary Table S6: Experimental data from Figure 5B. In vivo average radiant efficiency (%), measured in livers from mice treated with SCy-MiExo.

## Supplementary Materials

The following are available online at https://www.mdpi.com/2227-9059/9/1/81/s1.
