# Supplementary material for: Covalently Labeled Fluorescent Exosomes for In Vitro and In Vivo Applications
Source: Biomedicines. 2021 Jan 16;9(1):81. doi: 10.3390/biomedicines9010081 (PMC7829962; doi:10.3390/biomedicines9010081)
Supplement: Supplementary file 1 [file biomedicines-09-00081-s001.pdf]

biomedicines

MDPI

Covalently Labeled Fluorescent Exosomes for In vitro and In vivo Applications

María Isabel González [1,2], Mario González-Arjona [1,2], Ana Santos-Coquillat [1,2], Javier Vaquero [3,4], Elena Vázquez-Ogando [3], Antonio de Molina [5], Héctor Peinado [6], Manuel Desco [1,2,7,8,]* and Beatriz Salinas [1,2,7,8*]

[1] Unidad de Medicina y Cirugía Experimental, Instituto de Investigación Sanitaria Gregorio Marañón (IiSGM), 28007 Madrid, Spain; migonzalez@hggm.es (M.I.G.); mgarjona@hggm.es (M.G-A.); ascoquillat@hggm.es (A.S-C.); bsalinas@hggm.es (B.S.)

[2] Unidad de Imagen Avanzada, Centro Nacional de Investigaciones Cardiovasculares (CNIC), 28029 Madrid, Spain

[3] HepatoGastro Lab, Servicio de Ap. Digestivo del HGU Gregorio Marañón, Instituto de Investigación Sanitaria Gregorio Marañón (IiSGM), 28007 Madrid, Spain; javiervaq@gmail.com (J.V.); elena.vazquez.ogando@hotmail.com (E.V-O.)

[4] Centro de Investigación Biomédica en Red en Enfermedades Hepáticas y Digestivas (CIBEREHD), 28029 Madrid, Spain

[5] Comparative Medicine Unit, Centro Nacional de Investigaciones Cardiovasculares (CNIC), 28029 Madrid, Spain; ademolina@cnic.es (A.M.)

[6] Microenvironment and Metastasis Laboratory, Department of Molecular Oncology, Spanish National Cancer Research Center (CNIO), 28029, Madrid Spain; hpeinado@cnio.es (H.P.)

[7] Departamento de Bioingeniería e Ingeniería Aeroespacial, Universidad Carlos III de Madrid, 28911 Madrid, Spain

[8] Centro de Investigación Biomédica en Red de Salud Mental (CIBERSAM), 28029 Madrid, Spain

* Correspondence: desco@hggm.es (M.D.); bsalinas@hggm.es (B.S.)

For ensuring and maintaining the transparency and reproducibility of the results recorded, authors have provided the following research data, openly available as supplementary information:

biomedicines

MDPI

Supplementary Table 1: Experimental data from Figure 1C. Physicochemical characterization of control and fluorescently labeled milk exosomes: Size distributions established with DLS.

**Particle size** (nm)	**1 Control exo.** (intensity, a.u.)	**2 Control exo.** (intensity, a.u.)	**3 Control exo.** (intensity, a.u.)	**1 SCy-MiExo** (intensity, a.u.)	**2 SCy-MiExo** (intensity, a.u.)	**3 SCy-MiExo** (intensity, a.u.)	**1 BDP-MiExo** (intensity, a.u.)	**2 BDP-MiExo** (intensity, a.u.)	**3 BDP-MiExo** (intensity, a.u.)
0.4	0	0	0	0	0	0	0	0	0
0.463	0	0	0	0	0	0	0	0	0
0.536	0	0	0	0	0	0	0	0	0
0.621	0	0	0	0	0	0	0	0	0
0.719	0	0	0	0	0	0	0	0	0
0.833	0	0	0	0	0	0	0	0	0
0.965	0	0	0	0	0	0	0	0	0
1.12	0	0	0	0	0	0	0	0	0
1.29	0	0	0	0	0	0	0	0	0
1.5	0	0	0	0	0	0	0	0	0
1.74	0	0	0	0	0	0	0	0	0
2.01	0	0	0	0	0	0	0	0	0
2.33	0	0	0	0	0	0	0	0	0
2.7	0	0	0	0	0	0	0	0	0
3.12	0	0	0	0	0	0	0	0	0
3.62	0	0	0	0	0	0	0	0	0
4.19	0	0	0	0	0	0	0	0	0
4.85	0	0	0	0	0	0	0	0	0
5.61	0	0	0	0	0	0	0	0	0
6.5	0	0	0	0	0	0	0	0	0
7.53	0	0	0	0	0	0	0	0	0
8.72	0	0	0	0	0	0	0	0	0
10.1	0	0	0	0	0	0	0	0	0
11.7	0	0	0	0	0	0	0	0	0
13.545	0	0	0	0	0	0	0	0	0
15.686	0	0	0	0	0	0	0	0	0
18.166	0	0	0	0	0	0	0.005728814	0	0
21.037	0.013554217	0	0	0	0	0	0.016271186	0	0
24.363	0.028012048	0	0	0	0	0	0.021864407	0	0
28.214	0.02876506	0	0	0	0	0	0.020847458	0	0
32.674	0.017243976	0	0	0	0	0	0.022372881	0	0
37.84	0.011295181	0.010268698	0	0.013365385	0.009298969	0	0.04220339	0.025877193	0.029224138
43.821	0.035768072	0.074276913	0.032208589	0.0575	0.052268041	0.026902655	0.095762712	0.106140351	0.106896552
50.748	0.110466867	0.200667465	0.120168712	0.140384615	0.13814433	0.09380531	0.192372881	0.24122807	0.234482759
58.771	0.242093373	0.374636317	0.265644172	0.260576923	0.262886598	0.203539823	0.329661017	0.415789474	0.400862069
68.061	0.420331325	0.568714701	0.452147239	0.408653846	0.41443299	0.350442478	0.496610169	0.603508772	0.58362069

78.82	0.619503012	0.751754236	0.650843558	0.570192308	0.577319588	0.519469027	0.672033898	0.776315789	0.756034483
91.28	0.805045181	0.896286154	0.82791411	0.725961538	0.730927835	0.690265487	0.829661017	0.912280702	0.896551724
105.709	0.94126506	0.982286497	0.951840491	0.859615385	0.860824742	0.840707965	0.949152542	0.99122807	0.982758621
122.42	1	1	1	0.954807692	0.953608247	0.946902655	1	1	1
141.772	0.967243976	0.950111244	0.962960123	1	1	1	0.974576271	0.947368421	0.948275862
164.183	0.846762048	0.842204347	0.846625767	0.990384615	0.998969072	0.991150442	0.889830508	0.837719298	0.84137931
190.137	0.660240964	0.693051515	0.671165644	0.926923077	0.950515464	0.911504425	0.737288136	0.69122807	0.690517241
220.194	0.443674699	0.523446859	0.467714724	0.817307692	0.86185567	0.782300885	0.551694915	0.524561404	0.51637931
255.002	0.239909639	0.355211364	0.272392638	0.674038462	0.742268041	0.615929204	0.359322034	0.35877193	0.343103448
295.312	0.088027108	0.208112271	0.118788344	0.513461538	0.604123711	0.43539823	0.190677966	0.213157895	0.193103448
341.995	0.010918675	0.096868047	0.028220859	0.353846154	0.459793814	0.266371681	0.070932203	0.102631579	0.083275862
396.058	0	0.028495636	0	0.211538462	0.322680412	0.12920354	0.010508475	0.03254386	0.020517241
458.666	0	0.000770152	0	0.101923077	0.203092784	0.040265487	0	0.002666667	0
531.172	0	0	0	0.032211538	0.109278351	0.001681416	0	0	0
615.139	0	0	0	0.002192308	0.045360825	0	0	0	0
712.379	0	0	0	0	0.010927835	0	0	0	0
824.992	0	0	0	0	0	0	0	0	0
955.406	0	0	0	0	0	0	0	0	0
1106.435	0	0	0	0	0	0	0	0	0
1281.34	0	0	0	0	0	0	0	0	0
1483.893	0	0	0	0	0	0	0	0	0
1718.466	0	0	0	0	0	0	0	0	0
1990.119	0	0	0	0	0	0	0	0	0
2304.716	0	0	0	0	0	0	0	0	0
2669.043	0	0	0	0	0	0	0	0	0
3090.964	0	0	0	0	0	0	0	0	0

Supplementary Table 2: Experimental data from Figure 2B. Physicochemical characterization of control and fluorescently labeled cancer cell line-derived exosomes: Size distributions established with DLS.

**Particle size** (nm)	**1 Control U87 exo.** (intensity, a.u.)	**2 Control U87 exo.** (intensity, a.u.)	**1 SCy-U87Exo** (intensity, a.u.)	**2 SCy-U87Exo** (intensity, a.u.)	**3 SCy-U87Exo** (intensity, a.u.)	**1Control B16F10 exo.** (intensity, a.u.)	**2 Control B16F10 exo.** (intensity, a.u.)	**1 SCy-B16F10Exo** (intensity, a.u.)	**2 SCy-B16F10Exo** (intensity, a.u.)	**3 SCy-B16F10Exo** (intensity, a.u.)
0.4	0.00	0.00	0.00	0.00	0.00	0.00	0.00	0.00	0.00	0.00
0.463	0.00	0.00	0.00	0.00	0.00	0.00	0.00	0.00	0.00	0.00
0.536	0.00	0.00	0.00	0.00	0.00	0.00	0.00	0.00	0.00	0.00
0.621	0.00	0.00	0.00	0.00	0.00	0.00	0.00	0.00	0.00	0.00
0.719	0.00	0.00	0.00	0.00	0.00	0.00	0.00	0.00	0.00	0.00
0.833	0.00	0.00	0.00	0.00	0.00	0.00	0.00	0.00	0.00	0.00
0.965	0.00	0.00	0.00	0.00	0.00	0.00	0.00	0.00	0.00	0.00

biomedicines

MDPI

1.117	0.00	0.00	0.00	0.00	0.00	0.00	0.00	0.00	0.00	0.00
1.294	0.00	0.00	0.00	0.00	0.00	0.00	0.00	0.00	0.00	0.00
1.499	0.00	0.00	0.00	0.00	0.00	0.00	0.00	0.00	0.00	0.00
1.736	0.00	0.00	0.00	0.00	0.00	0.00	0.00	0.00	0.00	0.00
2.01	0.00	0.00	0.00	0.00	0.00	0.00	0.00	0.00	0.00	0.00
2.328	0.00	0.00	0.00	0.00	0.00	0.00	0.00	0.00	0.00	0.00
2.696	0.00	0.00	0.00	0.00	0.00	0.00	0.00	0.00	0.00	0.00
3.122	0.00	0.00	0.00	0.00	0.00	0.00	0.00	0.00	0.00	0.00
3.615	0.00	0.00	0.00	0.00	0.00	0.00	0.00	0.00	0.00	0.00
4.187	0.00	0.00	0.00	0.00	0.00	0.00	0.00	0.00	0.00	0.00
4.849	0.00	0.00	0.00	0.00	0.00	0.00	0.00	0.00	0.00	0.00
5.615	0.00	0.00	0.00	0.00	0.00	0.00	0.00	0.00	0.00	0.00
6.503	0.00	0.00	0.00	0.00	0.00	0.00	0.00	0.00	0.00	0.00
7.531	0.00	0.00	0.00	0.00	0.00	0.00	0.00	0.00	0.00	0.00
8.721	0.00	0.00	0.00	0.00	0.00	0.00	0.00	0.00	0.00	0.00
10.1	0.00	0.00	0.00	0.00	0.00	0.00	0.00	0.00	0.00	0.00
11.696	0.00	0.00	0.00	0.00	0.00	0.00	0.00	0.00	0.00	0.00
13.545	0.00	0.00	0.00	0.00	0.00	0.00	0.00	0.00	0.00	0.00
15.686	0.01	0.02	0.00	0.00	0.00	0.00	0.00	0.00	0.00	0.00
18.166	0.04	0.06	0.00	0.00	0.00	0.01	0.00	0.00	0.00	0.00
21.037	0.09	0.09	0.00	0.00	0.00	0.03	0.00	0.00	0.00	0.00
24.363	0.14	0.10	0.00	0.00	0.00	0.06	0.01	0.01	0.00	0.00
28.214	0.18	0.10	0.00	0.00	0.00	0.06	0.03	0.04	0.00	0.01
32.674	0.22	0.12	0.00	0.00	0.00	0.05	0.06	0.09	0.00	0.05
37.84	0.25	0.16	0.00	0.00	0.03	0.03	0.10	0.18	0.00	0.11
43.821	0.29	0.24	0.04	0.03	0.10	0.02	0.14	0.28	0.04	0.21
50.748	0.35	0.35	0.13	0.12	0.20	0.05	0.19	0.39	0.14	0.32
58.771	0.42	0.49	0.27	0.27	0.34	0.13	0.26	0.51	0.28	0.45
68.061	0.51	0.64	0.45	0.46	0.50	0.25	0.35	0.63	0.45	0.59
78.82	0.62	0.78	0.62	0.65	0.65	0.41	0.46	0.74	0.62	0.71
91.28	0.74	0.90	0.78	0.82	0.79	0.59	0.59	0.84	0.78	0.82
105.709	0.85	0.97	0.91	0.94	0.90	0.76	0.72	0.92	0.90	0.91
122.42	0.94	1.00	0.98	1.00	0.97	0.90	0.84	0.97	0.98	0.97
141.772	0.99	0.98	1.00	1.00	1.00	0.98	0.94	1.00	1.00	1.00
164.183	1.00	0.91	0.97	0.94	0.98	1.00	0.99	1.00	0.97	0.99
190.137	0.96	0.81	0.88	0.83	0.92	0.95	1.00	0.97	0.90	0.96
220.194	0.87	0.68	0.76	0.69	0.83	0.84	0.95	0.92	0.80	0.89
255.002	0.74	0.54	0.62	0.53	0.71	0.69	0.85	0.84	0.67	0.80
295.312	0.58	0.40	0.47	0.38	0.57	0.52	0.72	0.74	0.53	0.69
341.995	0.42	0.27	0.32	0.24	0.43	0.34	0.55	0.64	0.39	0.57
396.058	0.26	0.16	0.20	0.13	0.30	0.19	0.39	0.52	0.26	0.45

458.666	0.14	0.08	0.10	0.05	0.18	0.08	0.24	0.41	0.16	0.33
531.172	0.05	0.03	0.04	0.01	0.10	0.02	0.12	0.30	0.08	0.23
615.139	0.01	0.00	0.01	0.00	0.04	0.00	0.04	0.21	0.03	0.14
712.379	0.00	0.00	0.00	0.00	0.01	0.00	0.01	0.13	0.01	0.07
824.992	0.00	0.00	0.00	0.00	0.00	0.00	0.00	0.07	0.00	0.03
955.406	0.00	0.00	0.00	0.00	0.00	0.00	0.00	0.03	0.00	0.01
1106.435	0.00	0.00	0.00	0.00	0.00	0.00	0.00	0.01	0.00	0.00
1281.34	0.00	0.00	0.00	0.00	0.00	0.00	0.00	0.00	0.00	0.00
1483.893	0.00	0.00	0.00	0.00	0.00	0.00	0.00	0.00	0.00	0.00
1718.466	0.00	0.00	0.00	0.00	0.00	0.00	0.00	0.00	0.00	0.00
1990.119	0.00	0.00	0.00	0.00	0.00	0.00	0.00	0.00	0.00	0.00
2304.716	0.00	0.00	0.00	0.00	0.00	0.00	0.00	0.00	0.00	0.00
2669.043	0.00	0.01	0.00	0.00	0.00	0.00	0.00	0.00	0.00	0.00
3090.964	0.00	0.02	0.00	0.00	0.00	0.00	0.00	0.00	0.00	0.00

Supplementary Table 3: Experimental data from Figure 3. In vitro stability of BDP-MiExo evaluated by HPLC.

**Time** (min)	**BDP-MiExo t = 0h** (Intensity, normalized)	**BDP-MiExo t = 12h** (Intensity, normalized)	**BDP-MiExo t = 48h** (Intensity, normalized)	**BDP-MiExo t = 72h** (Intensity, normalized)	**Control exo.** (Intensity, normalized)	**free BDP** (Intensity, normalized)
0.00	0.00	0.00	-0.33	0.26	0.00	0.00
0.02	0.00	0.00	-0.33	0.26	0.00	0.00
0.03	0.00	-0.06	-0.01	0.16	0.01	0.00
0.05	0.09	0.00	0.00	0.00	0.01	0.00
0.07	0.14	0.00	0.00	0.00	0.02	-0.01
0.08	0.05	-0.01	0.00	0.01	0.02	-0.05
0.10	-0.02	0.00	0.00	0.00	0.01	-0.09
0.12	-0.02	0.00	0.01	0.00	0.01	-0.10
0.13	-0.09	0.01	0.01	-0.01	0.02	-0.10
0.15	-0.11	0.01	0.01	-0.01	0.02	-0.10
0.17	-0.02	0.01	0.01	-0.01	0.02	-0.12
0.18	0.06	0.01	0.01	-0.01	0.02	-0.13
0.20	0.15	0.02	0.01	-0.01	0.02	-0.13
0.22	0.22	0.02	0.01	-0.01	0.02	-0.13
0.23	0.17	0.02	0.02	-0.01	0.02	-0.14
0.25	0.03	0.03	0.02	-0.01	0.02	-0.14
0.27	-0.03	0.03	0.02	-0.01	0.02	-0.15
0.28	-0.03	0.04	0.02	-0.02	0.02	-0.16
0.30	-0.05	0.04	0.02	-0.02	0.02	-0.17
0.32	0.00	0.05	0.01	-0.03	0.02	-0.18
0.33	0.05	0.05	0.02	-0.03	0.02	-0.18
0.35	0.14	0.06	0.02	-0.03	0.02	-0.19
0.37	0.28	0.06	0.03	-0.03	0.02	-0.19
0.38	0.26	0.07	0.03	-0.04	0.02	-0.20
0.40	0.22	0.07	0.03	-0.03	0.02	-0.20
0.42	0.26	0.08	0.04	-0.03	0.02	-0.20
0.43	0.29	0.08	0.06	-0.04	0.02	-0.20
0.45	0.34	0.08	0.09	-0.04	0.02	-0.20
0.47	0.37	0.09	0.13	-0.04	0.02	-0.21
0.48	0.45	0.10	0.17	-0.04	0.02	-0.21
0.50	0.57	0.10	0.20	-0.04	0.02	-0.21
0.52	0.66	0.11	0.22	-0.05	0.02	-0.23
0.53	0.71	0.11	0.24	-0.05	0.02	-0.24
0.55	0.70	0.12	0.24	-0.05	0.03	-0.25

0.57	0.68	0.13	0.24	-0.05	0.03	-0.26
0.58	0.59	0.14	0.25	-0.05	0.03	-0.26
0.60	0.48	0.14	0.25	-0.05	0.03	-0.26
0.62	0.46	0.15	0.25	-0.05	0.03	-0.27
0.63	0.45	0.16	0.26	-0.04	0.03	-0.27
0.65	0.53	0.16	0.26	-0.04	0.03	-0.27
0.67	0.63	0.17	0.27	-0.04	0.03	-0.28
0.68	0.60	0.18	0.28	-0.03	0.03	-0.28
0.70	0.51	0.18	0.30	-0.03	0.03	-0.29
0.72	0.59	0.19	0.32	-0.03	0.03	-0.29
0.73	0.70	0.19	0.34	-0.02	0.03	-0.30
0.75	0.68	0.20	0.37	-0.02	0.03	-0.30
0.77	0.66	0.21	0.41	-0.01	0.03	-0.31
0.78	0.73	0.22	0.45	-0.01	0.03	-0.32
0.80	0.71	0.22	0.49	-0.01	0.03	-0.32
0.82	0.59	0.23	0.54	-0.01	0.03	-0.33
0.83	0.49	0.23	0.60	-0.02	0.02	-0.34
0.85	0.46	0.24	0.68	-0.02	0.02	-0.34
0.87	0.48	0.25	0.76	-0.03	0.02	-0.34
0.88	0.43	0.26	0.83	-0.03	0.02	-0.34
0.90	0.29	0.27	0.89	-0.04	0.02	-0.35
0.92	0.25	0.28	0.93	-0.05	0.02	-0.35
0.93	0.28	0.29	0.94	-0.06	0.02	-0.36
0.95	0.23	0.29	0.93	-0.06	0.02	-0.38
0.97	0.31	0.30	0.89	-0.06	0.02	-0.39
0.98	0.51	0.31	0.85	-0.07	0.02	-0.40
1.00	0.56	0.32	0.81	-0.07	0.02	-0.40
1.02	0.46	0.32	0.76	-0.08	0.02	-0.40
1.03	0.45	0.33	0.72	-0.08	0.02	-0.41
1.05	0.48	0.34	0.69	-0.09	0.02	-0.41
1.07	0.46	0.35	0.65	-0.09	0.02	-0.41
1.08	0.45	0.36	0.63	-0.09	0.02	-0.42
1.10	0.57	0.37	0.61	-0.10	0.02	-0.43
1.12	0.63	0.38	0.59	-0.10	0.02	-0.44
1.13	0.59	0.38	0.57	-0.10	0.02	-0.45
1.15	0.62	0.39	0.56	-0.11	0.02	-0.46
1.17	0.60	0.40	0.54	-0.12	0.02	-0.45
1.18	0.56	0.41	0.53	-0.12	0.02	-0.45
1.20	0.54	0.42	0.52	-0.12	0.02	-0.46
1.22	0.53	0.43	0.51	-0.12	0.02	-0.46
1.23	0.49	0.44	0.50	-0.12	0.02	-0.47
1.25	0.45	0.45	0.49	-0.12	0.02	-0.48
1.27	0.37	0.46	0.49	-0.13	0.02	-0.49
1.28	0.37	0.47	0.48	-0.13	0.02	-0.50
1.30	0.45	0.47	0.48	-0.13	0.02	-0.50
1.32	0.42	0.48	0.47	-0.13	0.02	-0.51
1.33	0.46	0.49	0.47	-0.14	0.02	-0.51
1.35	0.51	0.51	0.47	-0.14	0.02	-0.52
1.37	0.49	0.52	0.46	-0.14	0.02	-0.52
1.38	0.59	0.53	0.46	-0.14	0.02	-0.52
1.40	0.54	0.53	0.46	-0.14	0.02	-0.53
1.42	0.46	0.55	0.46	-0.15	0.02	-0.53
1.43	0.51	0.55	0.46	-0.15	0.02	-0.53
1.45	0.57	0.56	0.46	-0.15	0.02	-0.54
1.47	0.51	0.57	0.46	-0.15	0.02	-0.54
1.48	0.46	0.58	0.46	-0.16	0.02	-0.55
1.50	0.49	0.60	0.46	-0.16	0.02	-0.56
1.52	0.45	0.61	0.47	-0.17	0.02	-0.57
1.53	0.46	0.62	0.47	-0.16	0.02	-0.56
1.55	0.59	0.63	0.47	-0.16	0.02	-0.56
1.57	0.56	0.64	0.48	-0.17	0.02	-0.56
1.58	0.56	0.65	0.48	-0.17	0.02	-0.57
1.60	0.54	0.66	0.48	-0.17	0.02	-0.57
1.62	0.46	0.68	0.49	-0.17	0.02	-0.58
1.63	0.46	0.69	0.50	-0.17	0.02	-0.58
1.65	0.54	0.70	0.50	-0.17	0.02	-0.59
1.67	0.63	0.72	0.51	-0.17	0.02	-0.60
1.68	0.68	0.73	0.51	-0.17	0.02	-0.60
1.70	0.70	0.75	0.52	-0.18	0.02	-0.60

1.72	0.66	0.75	0.53	-0.18	0.02	-0.60
1.73	0.59	0.77	0.53	-0.18	0.02	-0.61
1.75	0.57	0.78	0.54	-0.18	0.02	-0.61
1.77	0.59	0.79	0.55	-0.18	0.02	-0.62
1.78	0.53	0.81	0.55	-0.18	0.02	-0.62
1.80	0.48	0.83	0.56	-0.19	0.02	-0.63
1.82	0.62	0.84	0.57	-0.19	0.02	-0.63
1.83	0.77	0.85	0.58	-0.19	0.02	-0.63
1.85	0.79	0.87	0.59	-0.19	0.02	-0.64
1.87	0.68	0.89	0.60	-0.19	0.02	-0.65
1.88	0.63	0.91	0.61	-0.20	0.02	-0.65
1.90	0.66	0.92	0.62	-0.20	0.02	-0.65
1.92	0.79	0.94	0.63	-0.20	0.02	-0.66
1.93	0.91	0.95	0.64	-0.20	0.02	-0.66
1.95	0.83	0.97	0.64	-0.20	0.02	-0.66
1.97	0.71	0.99	0.65	-0.20	0.02	-0.66
1.98	0.70	1.00	0.66	-0.21	0.02	-0.67
2.00	0.77	1.02	0.67	-0.21	0.02	-0.68
2.02	0.74	1.04	0.69	-0.21	0.02	-0.68
2.03	0.66	1.05	0.70	-0.21	0.02	-0.68
2.05	0.65	1.07	0.71	-0.21	0.02	-0.68
2.07	0.62	1.09	0.72	-0.21	0.02	-0.68
2.08	0.59	1.11	0.73	-0.21	0.02	-0.69
2.10	0.65	1.12	0.74	-0.21	0.02	-0.70
2.12	0.82	1.14	0.76	-0.22	0.02	-0.70
2.13	0.96	1.16	0.77	-0.22	0.02	-0.71
2.15	0.88	1.18	0.78	-0.22	0.02	-0.71
2.17	0.76	1.20	0.79	-0.23	0.02	-0.71
2.18	0.79	1.22	0.81	-0.23	0.02	-0.72
2.20	0.79	1.24	0.82	-0.23	0.02	-0.72
2.22	0.79	1.26	0.83	-0.23	0.02	-0.73
2.23	0.77	1.28	0.84	-0.23	0.02	-0.74
2.25	0.76	1.30	0.86	-0.23	0.02	-0.73
2.27	0.94	1.32	0.87	-0.23	0.02	-0.73
2.28	1.13	1.34	0.89	-0.23	0.02	-0.74
2.30	1.02	1.35	0.90	-0.23	0.02	-0.75
2.32	0.93	1.37	0.91	-0.23	0.02	-0.75
2.33	0.96	1.39	0.93	-0.23	0.02	-0.75
2.35	1.00	1.41	0.94	-0.23	0.02	-0.75
2.37	1.05	1.43	0.95	-0.23	0.02	-0.75
2.38	1.07	1.45	0.97	-0.24	0.02	-0.74
2.40	1.10	1.46	0.98	-0.24	0.02	-0.75
2.42	1.08	1.47	1.00	-0.24	0.02	-0.76
2.43	1.00	1.48	1.01	-0.24	0.02	-0.76
2.45	0.90	1.50	1.03	-0.24	0.02	-0.76
2.47	0.83	1.52	1.04	-0.23	0.02	-0.77
2.48	0.90	1.53	1.06	-0.23	0.02	-0.78
2.50	1.17	1.54	1.07	-0.23	0.02	-0.78
2.52	1.25	1.55	1.09	-0.23	0.02	-0.79
2.53	1.00	1.56	1.11	-0.23	0.02	-0.78
2.55	0.85	1.57	1.12	-0.23	0.02	-0.79
2.57	0.91	1.58	1.13	-0.23	0.02	-0.79
2.58	1.02	1.60	1.15	-0.22	0.02	-0.79
2.60	1.04	1.60	1.17	-0.22	0.02	-0.79
2.62	1.04	1.61	1.19	-0.22	0.02	-0.79
2.63	1.05	1.61	1.20	-0.21	0.02	-0.80
2.65	1.05	1.62	1.22	-0.20	0.02	-0.81
2.67	1.04	1.62	1.23	-0.20	0.02	-0.82
2.68	1.13	1.63	1.25	-0.19	0.02	-0.82
2.70	1.27	1.63	1.27	-0.18	0.02	-0.81
2.72	1.17	1.63	1.29	-0.17	0.02	-0.80
2.73	1.00	1.63	1.30	-0.16	0.02	-0.80
2.75	0.99	1.62	1.32	-0.15	0.02	-0.81
2.77	1.07	1.62	1.34	-0.13	0.02	-0.81
2.78	1.21	1.62	1.36	-0.09	0.02	-0.82
2.80	1.36	1.62	1.37	-0.05	0.02	-0.83
2.82	1.41	1.61	1.39	0.02	0.02	-0.83
2.83	1.33	1.61	1.41	0.11	0.02	-0.83
2.85	1.33	1.60	1.42	0.24	0.02	-0.84

2.87	1.41	1.59	1.44	0.39	0.02	-0.84
2.88	1.34	1.59	1.46	0.57	0.02	-0.84
2.90	1.25	1.57	1.48	0.78	0.02	-0.84
2.92	1.34	1.56	1.50	1.00	0.02	-0.85
2.93	1.45	1.55	1.51	1.23	0.02	-0.85
2.95	1.41	1.53	1.53	1.46	0.02	-0.85
2.97	1.28	1.51	1.55	1.69	0.02	-0.85
2.98	1.36	1.49	1.57	1.93	0.02	-0.86
3.00	1.55	1.47	1.58	2.18	0.02	-0.87
3.02	1.53	1.45	1.60	2.44	0.02	-0.87
3.03	1.50	1.43	1.62	2.73	0.02	-0.87
3.05	1.55	1.40	1.64	3.04	0.02	-0.87
3.07	1.51	1.38	1.65	3.37	0.02	-0.88
3.08	1.47	1.35	1.67	3.72	0.02	-0.88
3.10	1.47	1.33	1.69	4.06	0.02	-0.88
3.12	1.47	1.30	1.71	4.40	0.02	-0.88
3.13	1.50	1.27	1.73	4.71	0.02	-0.89
3.15	1.50	1.23	1.74	5.00	0.02	-0.89
3.17	1.48	1.20	1.76	5.27	0.03	-0.89
3.18	1.51	1.16	1.78	5.52	0.03	-0.89
3.20	1.51	1.13	1.80	5.77	0.02	-0.90
3.22	1.45	1.10	1.81	6.04	0.02	-0.90
3.23	1.48	1.06	1.83	6.34	0.02	-0.90
3.25	1.58	1.03	1.85	6.69	0.02	-0.90
3.27	1.56	0.99	1.86	7.08	0.02	-0.90
3.28	1.47	0.95	1.88	7.52	0.02	-0.91
3.30	1.41	0.92	1.90	8.00	0.02	-0.91
3.32	1.45	0.89	1.91	8.52	0.02	-0.90
3.33	1.61	0.86	1.93	9.08	0.02	-0.91
3.35	1.61	0.82	1.95	9.66	0.02	-0.92
3.37	1.41	0.79	1.96	10.26	0.02	-0.92
3.38	1.41	0.76	1.98	10.88	0.02	-0.92
3.40	1.56	0.73	2.00	11.51	0.02	-0.92
3.42	1.59	0.69	2.01	12.16	0.02	-0.92
3.43	1.56	0.66	2.03	12.82	0.02	-0.92
3.45	1.53	0.64	2.05	13.47	0.02	-0.92
3.47	1.53	0.61	2.06	14.13	0.02	-0.92
3.48	1.56	0.59	2.08	14.79	0.02	-0.93
3.50	1.64	0.56	2.09	15.43	0.02	-0.94
3.52	1.68	0.54	2.11	16.07	0.02	-0.94
3.53	1.62	0.52	2.12	16.69	0.02	-0.94
3.55	1.59	0.50	2.14	17.30	0.02	-0.94
3.57	1.56	0.48	2.15	17.88	0.02	-0.95
3.58	1.58	0.46	2.17	18.44	0.02	-0.95
3.60	1.50	0.45	2.18	18.97	0.02	-0.94
3.62	1.38	0.43	2.20	19.48	0.02	-0.94
3.63	1.42	0.41	2.21	19.96	0.02	-0.95
3.65	1.59	0.39	2.22	20.39	0.02	-0.95
3.67	1.67	0.38	2.24	20.79	0.02	-0.95
3.68	1.72	0.36	2.25	21.15	0.02	-0.95
3.70	1.79	0.35	2.26	21.47	0.02	-0.96
3.72	1.78	0.34	2.27	21.73	0.02	-0.97
3.73	1.76	0.33	2.29	21.95	0.02	-0.97
3.75	1.76	0.32	2.30	22.12	0.02	-0.97
3.77	1.82	0.30	2.31	22.25	0.02	-0.97
3.78	1.85	0.29	2.32	22.32	0.02	-0.97
3.80	1.81	0.28	2.33	22.35	0.02	-0.97
3.82	1.78	0.28	2.34	22.33	0.02	-0.97
3.83	1.73	0.27	2.36	22.27	0.02	-0.97
3.85	1.61	0.26	2.37	22.17	0.02	-0.98
3.87	1.72	0.25	2.38	22.02	0.02	-0.98
3.88	2.01	0.25	2.39	21.83	0.02	-0.99
3.90	2.10	0.24	2.39	21.61	0.02	-1.00
3.92	2.04	0.23	2.40	21.34	0.03	-1.00
3.93	2.06	0.22	2.41	21.05	0.02	-1.00
3.95	2.06	0.22	2.42	20.72	0.02	-1.00
3.97	2.06	0.21	2.43	20.37	0.02	-1.00
3.98	2.04	0.21	2.44	19.99	0.03	-1.01
4.00	2.02	0.20	2.45	19.59	0.03	-1.01

4.02	2.10	0.20	2.45	19.17	0.03	-1.01
4.03	2.12	0.19	2.46	18.73	0.03	-1.01
4.05	2.07	0.19	2.47	18.28	0.03	-1.01
4.07	2.10	0.19	2.47	17.82	0.03	-1.01
4.08	2.23	0.18	2.48	17.35	0.02	-1.01
4.10	2.23	0.18	2.48	16.86	0.02	-1.02
4.12	2.10	0.17	2.49	16.38	0.03	-1.02
4.13	2.15	0.17	2.49	15.89	0.03	-1.02
4.15	2.15	0.17	2.50	15.40	0.03	-1.02
4.17	2.07	0.17	2.50	14.91	0.03	-1.01
4.18	2.12	0.17	2.51	14.42	0.03	-1.02
4.20	2.15	0.16	2.51	13.94	0.02	-1.03
4.22	2.10	0.16	2.51	13.46	0.02	-1.03
4.23	2.07	0.16	2.52	12.99	0.03	-1.03
4.25	2.09	0.15	2.52	12.53	0.03	-1.04
4.27	2.07	0.15	2.52	12.08	0.02	-1.04
4.28	2.09	0.15	2.52	11.64	0.03	-1.04
4.30	2.18	0.15	2.53	11.20	0.03	-1.03
4.32	2.16	0.15	2.53	10.78	0.03	-1.04
4.33	2.09	0.15	2.53	10.37	0.03	-1.04
4.35	2.07	0.15	2.53	9.97	0.03	-1.03
4.37	2.04	0.15	2.53	9.59	0.03	-1.04
4.38	2.09	0.15	2.53	9.21	0.03	-1.04
4.40	2.18	0.14	2.53	8.85	0.03	-1.05
4.42	2.16	0.14	2.53	8.50	0.03	-1.05
4.43	2.07	0.14	2.53	8.16	0.03	-1.04
4.45	2.07	0.14	2.53	7.84	0.03	-1.04
4.47	2.15	0.14	2.53	7.54	0.03	-1.05
4.48	2.07	0.14	2.52	7.24	0.03	-1.05
4.50	2.01	0.14	2.52	6.95	0.02	-1.06
4.52	2.06	0.14	2.52	6.67	0.02	-1.06
4.53	2.06	0.14	2.52	6.40	0.03	-1.06
4.55	2.16	0.14	2.52	6.15	0.03	-1.06
4.57	2.27	0.14	2.51	5.90	0.03	-1.06
4.58	2.16	0.14	2.51	5.67	0.03	-1.06
4.60	2.12	0.14	2.51	5.44	0.03	-1.07
4.62	2.21	0.14	2.51	5.23	0.03	-1.07
4.63	2.32	0.14	2.50	5.02	0.03	-1.07
4.65	2.29	0.14	2.50	4.82	0.03	-1.08
4.67	2.18	0.14	2.49	4.63	0.03	-1.08
4.68	2.19	0.14	2.49	4.44	0.03	-1.08
4.70	2.12	0.14	2.49	4.27	0.03	-1.08
4.72	2.09	0.14	2.48	4.10	0.03	-1.08
4.73	2.15	0.14	2.48	3.93	0.03	-1.08
4.75	2.18	0.14	2.47	3.78	0.03	-1.08
4.77	2.16	0.14	2.47	3.63	0.03	-1.08
4.78	2.19	0.14	2.47	3.48	0.03	-1.08
4.80	2.29	0.14	2.46	3.35	0.03	-1.09
4.82	2.30	0.14	2.46	3.21	0.03	-1.09
4.83	2.24	0.14	2.45	3.09	0.03	-1.09
4.85	2.23	0.14	2.45	2.96	0.03	-1.09
4.87	2.12	0.14	2.44	2.85	0.03	-1.09
4.88	2.01	0.14	2.44	2.73	0.03	-1.10
4.90	2.01	0.13	2.43	2.62	0.03	-1.11
4.92	2.07	0.13	2.42	2.52	0.03	-1.11
4.93	2.15	0.13	2.42	2.41	0.03	-1.10
4.95	2.13	0.13	2.42	2.32	0.03	-1.09
4.97	2.18	0.13	2.41	2.22	0.03	-1.10
4.98	2.36	0.13	2.41	2.14	0.03	-1.11
5.00	2.36	0.13	2.40	2.05	0.03	-1.11
5.02	2.26	0.13	2.40	1.96	0.03	-1.11
5.03	2.27	0.13	2.40	1.88	0.03	-1.12
5.05	2.38	0.13	2.39	1.80	0.03	-1.12
5.07	2.43	0.12	2.39	1.72	0.03	-1.12
5.08	2.35	0.12	2.38	1.65	0.03	-1.12
5.10	2.33	0.12	2.38	1.58	0.03	-1.12
5.12	2.41	0.12	2.38	1.51	0.03	-1.12
5.13	2.35	0.11	2.38	1.44	0.03	-1.12
5.15	2.26	0.11	2.37	1.38	0.03	-1.12

5.17	2.23	0.11	2.37	1.32	0.03	-1.12
5.18	2.26	0.10	2.37	1.26	0.03	-1.12
5.20	2.41	0.10	2.36	1.20	0.03	-1.12
5.22	2.53	0.10	2.36	1.14	0.03	-1.12
5.23	2.46	0.09	2.36	1.09	0.03	-1.12
5.25	2.32	0.08	2.36	1.03	0.03	-1.12
5.27	2.38	0.08	2.36	0.98	0.03	-1.13
5.28	2.47	0.08	2.36	0.94	0.03	-1.13
5.30	2.46	0.07	2.36	0.89	0.03	-1.13
5.32	2.32	0.07	2.36	0.84	0.03	-1.14
5.33	2.24	0.06	2.36	0.80	0.03	-1.15
5.35	2.36	0.05	2.36	0.75	0.03	-1.15
5.37	2.40	0.05	2.37	0.71	0.02	-1.15
5.38	2.33	0.05	2.37	0.67	0.03	-1.14
5.40	2.35	0.04	2.37	0.63	0.03	-1.14
5.42	2.33	0.03	2.37	0.59	0.03	-1.14
5.43	2.30	0.02	2.38	0.55	0.03	-1.14
5.45	2.38	0.02	2.38	0.52	0.03	-1.15
5.47	2.43	0.01	2.38	0.49	0.02	-1.16
5.48	2.46	0.01	2.39	0.45	0.02	-1.16
5.50	2.46	0.01	2.39	0.41	0.03	-1.16
5.52	2.46	0.00	2.40	0.38	0.03	-1.16
5.53	2.50	-0.01	2.40	0.35	0.02	-1.16
5.55	2.41	-0.01	2.41	0.32	0.02	-1.16
5.57	2.35	-0.02	2.42	0.29	0.02	-1.16
5.58	2.43	-0.03	2.42	0.25	0.03	-1.15
5.60	2.52	-0.03	2.43	0.22	0.03	-1.16
5.62	2.58	-0.04	2.44	0.20	0.03	-1.16
5.63	2.58	-0.04	2.44	0.18	0.03	-1.17
5.65	2.60	-0.05	2.45	0.15	0.03	-1.16
5.67	2.55	-0.05	2.46	0.12	0.03	-1.16
5.68	2.60	-0.06	2.47	0.10	0.02	-1.16
5.70	2.69	-0.06	2.48	0.08	0.02	-1.17
5.72	2.63	-0.07	2.49	0.05	0.02	-1.16
5.73	2.58	-0.07	2.50	0.03	0.02	-1.16
5.75	2.49	-0.07	2.51	0.01	0.03	-1.17
5.77	2.38	-0.07	2.52	-0.01	0.03	-1.18
5.78	2.44	-0.07	2.53	-0.03	0.03	-1.18
5.80	2.57	-0.08	2.55	-0.05	0.03	-1.18
5.82	2.55	-0.08	2.56	-0.07	0.03	-1.18
5.83	2.53	-0.09	2.57	-0.09	0.03	-1.18
5.85	2.58	-0.09	2.58	-0.11	0.03	-1.18
5.87	2.55	-0.09	2.59	-0.12	0.02	-1.19
5.88	2.46	-0.10	2.61	-0.13	0.02	-1.19
5.90	2.46	-0.10	2.62	-0.15	0.02	-1.19
5.92	2.57	-0.10	2.63	-0.17	0.02	-1.19
5.93	2.57	-0.11	2.64	-0.19	0.02	-1.19
5.95	2.50	-0.11	2.66	-0.21	0.02	-1.19
5.97	2.55	-0.11	2.68	-0.22	0.02	-1.19
5.98	2.64	-0.11	2.69	-0.23	0.02	-1.19
6.00	2.60	-0.11	2.70	-0.25	0.02	-1.19
6.02	2.58	-0.11	2.72	-0.26	0.02	-1.19
6.03	2.60	-0.12	2.73	-0.27	0.02	-1.19
6.05	2.49	-0.12	2.74	-0.29	0.03	-1.20
6.07	2.60	-0.13	2.76	-0.30	0.03	-1.20
6.08	2.67	-0.12	2.77	-0.31	0.02	-1.20
6.10	2.64	-0.13	2.78	-0.32	0.02	-1.20
6.12	2.60	-0.13	2.80	-0.33	0.02	-1.20
6.13	2.55	-0.14	2.81	-0.34	0.02	-1.20
6.15	2.66	-0.14	2.82	-0.35	0.02	-1.20
6.17	2.78	-0.14	2.83	-0.37	0.02	-1.20
6.18	2.86	-0.14	2.85	-0.37	0.02	-1.20
6.20	2.81	-0.14	2.86	-0.38	0.02	-1.21
6.22	2.69	-0.14	2.87	-0.39	0.02	-1.21
6.23	2.72	-0.14	2.89	-0.39	0.02	-1.20
6.25	2.77	-0.15	2.90	-0.40	0.02	-1.20
6.27	2.69	-0.15	2.91	-0.41	0.02	-1.21
6.28	2.57	-0.15	2.92	-0.42	0.02	-1.21
6.30	2.47	-0.15	2.93	-0.43	0.02	-1.21

6.32	2.52	-0.15	2.94	-0.44	0.02	-1.21
6.33	2.57	-0.16	2.95	-0.45	0.02	-1.22
6.35	2.57	-0.16	2.96	-0.45	0.02	-1.22
6.37	2.49	-0.16	2.97	-0.46	0.02	-1.22
6.38	2.52	-0.16	2.98	-0.46	0.02	-1.22
6.40	2.64	-0.16	2.98	-0.47	0.02	-1.22
6.42	2.60	-0.16	2.99	-0.48	0.02	-1.22
6.43	2.57	-0.16	3.00	-0.49	0.02	-1.22
6.45	2.66	-0.16	3.01	-0.49	0.02	-1.22
6.47	2.74	-0.17	3.01	-0.50	0.03	-1.22
6.48	2.72	-0.17	3.02	-0.50	0.02	-1.23
6.50	2.67	-0.17	3.02	-0.51	0.02	-1.23
6.52	2.67	-0.17	3.02	-0.51	0.02	-1.23
6.53	2.67	-0.17	3.03	-0.51	0.02	-1.23
6.55	2.70	-0.17	3.03	-0.52	0.02	-1.22
6.57	2.67	-0.17	3.04	-0.52	0.02	-1.22
6.58	2.66	-0.17	3.04	-0.53	0.02	-1.22
6.60	2.77	-0.18	3.04	-0.54	0.02	-1.23
6.62	2.83	-0.17	3.04	-0.54	0.03	-1.23
6.63	2.80	-0.18	3.04	-0.55	0.02	-1.23
6.65	2.78	-0.18	3.04	-0.55	0.02	-1.23
6.67	2.78	-0.18	3.03	-0.55	0.02	-1.23
6.68	2.87	-0.18	3.03	-0.56	0.02	-1.23
6.70	2.97	-0.18	3.03	-0.56	0.02	-1.23
6.72	2.84	-0.18	3.03	-0.56	0.02	-1.23
6.73	2.64	-0.18	3.02	-0.57	0.03	-1.24
6.75	2.58	-0.19	3.02	-0.57	0.02	-1.24
6.77	2.72	-0.19	3.01	-0.58	0.02	-1.24
6.78	2.86	-0.19	3.01	-0.58	0.03	-1.24
6.80	2.86	-0.19	3.00	-0.58	0.03	-1.23
6.82	2.86	-0.18	2.99	-0.58	0.02	-1.23
6.83	2.81	-0.18	2.98	-0.59	0.03	-1.24
6.85	2.81	-0.19	2.97	-0.59	0.02	-1.24
6.87	2.75	-0.19	2.96	-0.59	0.03	-1.24
6.88	2.64	-0.19	2.95	-0.60	0.03	-1.25
6.90	2.74	-0.19	2.94	-0.60	0.03	-1.24
6.92	2.80	-0.19	2.93	-0.60	0.03	-1.25
6.93	2.80	-0.19	2.92	-0.60	0.03	-1.25
6.95	2.83	-0.19	2.91	-0.61	0.02	-1.25
6.97	2.77	-0.19	2.89	-0.61	0.02	-1.25
6.98	2.70	-0.19	2.88	-0.61	0.02	-1.25
7.00	2.72	-0.19	2.86	-0.61	0.02	-1.25
7.02	2.67	-0.19	2.84	-0.61	0.02	-1.25
7.03	2.64	-0.19	2.83	-0.62	0.02	-1.26
7.05	2.81	-0.19	2.81	-0.62	0.03	-1.26
7.07	2.92	-0.19	2.80	-0.62	0.03	-1.26
7.08	2.84	-0.19	2.78	-0.63	0.02	-1.26
7.10	2.84	-0.20	2.76	-0.63	0.02	-1.26
7.12	2.87	-0.20	2.74	-0.63	0.02	-1.26
7.13	2.77	-0.20	2.72	-0.63	0.02	-1.27
7.15	2.72	-0.20	2.70	-0.63	0.02	-1.27
7.17	2.72	-0.20	2.68	-0.63	0.03	-1.27
7.18	2.60	-0.20	2.66	-0.64	0.03	-1.27
7.20	2.52	-0.20	2.64	-0.64	0.02	-1.27
7.22	2.57	-0.20	2.62	-0.64	0.02	-1.28
7.23	2.72	-0.20	2.59	-0.64	0.03	-1.28
7.25	2.86	-0.21	2.57	-0.64	0.03	-1.28
7.27	2.81	-0.21	2.55	-0.64	0.03	-1.28
7.28	2.69	-0.21	2.52	-0.65	0.02	-1.27
7.30	2.70	-0.21	2.50	-0.65	0.02	-1.27
7.32	2.67	-0.21	2.48	-0.65	0.03	-1.27
7.33	2.64	-0.21	2.45	-0.65	0.03	-1.28
7.35	2.72	-0.21	2.43	-0.65	0.03	-1.28
7.37	2.87	-0.21	2.40	-0.66	0.03	-1.27
7.38	3.00	-0.21	2.38	-0.67	0.03	-1.27
7.40	3.00	-0.21	2.35	-0.67	0.03	-1.28
7.42	2.95	-0.21	2.32	-0.67	0.03	-1.28
7.43	2.98	-0.21	2.30	-0.67	0.03	-1.29
7.45	3.01	-0.22	2.27	-0.68	0.03	-1.29

7.47	2.89	-0.21	2.24	-0.68	0.03	-1.29
7.48	2.75	-0.21	2.21	-0.68	0.03	-1.28
7.50	2.80	-0.21	2.19	-0.68	0.03	-1.28
7.52	2.92	-0.21	2.16	-0.68	0.03	-1.28
7.53	2.92	-0.21	2.13	-0.69	0.03	-1.28
7.55	2.89	-0.21	2.11	-0.69	0.03	-1.28
7.57	3.01	-0.22	2.08	-0.68	0.02	-1.28
7.58	3.12	-0.21	2.05	-0.69	0.03	-1.29
7.60	3.01	-0.21	2.03	-0.69	0.03	-1.29
7.62	2.83	-0.22	2.00	-0.69	0.03	-1.29
7.63	2.77	-0.22	1.97	-0.69	0.02	-1.30
7.65	2.84	-0.21	1.94	-0.69	0.03	-1.30
7.67	3.01	-0.21	1.91	-0.69	0.03	-1.30
7.68	3.09	-0.21	1.89	-0.70	0.03	-1.30
7.70	3.04	-0.22	1.86	-0.70	0.03	-1.30
7.72	2.98	-0.22	1.83	-0.70	0.03	-1.30
7.73	2.95	-0.22	1.81	-0.69	0.03	-1.30
7.75	3.03	-0.22	1.78	-0.69	0.03	-1.30
7.77	3.09	-0.22	1.75	-0.69	0.03	-1.30
7.78	3.09	-0.22	1.73	-0.69	0.03	-1.31
7.80	2.98	-0.22	1.70	-0.69	0.03	-1.30
7.82	2.87	-0.22	1.67	-0.69	0.02	-1.30
7.83	2.92	-0.22	1.65	-0.69	0.02	-1.31
7.85	2.94	-0.22	1.62	-0.70	0.02	-1.31
7.87	2.94	-0.22	1.60	-0.70	0.03	-1.31
7.88	2.89	-0.22	1.57	-0.70	0.03	-1.31
7.90	2.95	-0.23	1.55	-0.69	0.02	-1.32
7.92	3.00	-0.23	1.52	-0.70	0.02	-1.32
7.93	2.92	-0.23	1.50	-0.70	0.02	-1.32
7.95	3.04	-0.22	1.47	-0.70	0.03	-1.31
7.97	3.20	-0.22	1.45	-0.70	0.03	-1.31
7.98	3.18	-0.22	1.42	-0.70	0.02	-1.31
8.00	3.09	-0.22	1.40	-0.70	0.02	-1.32
8.02	3.03	-0.22	1.38	-0.71	0.02	-1.32
8.03	3.09	-0.22	1.35	-0.71	0.02	-1.32
8.05	3.21	-0.22	1.33	-0.71	0.02	-1.32
8.07	3.21	-0.22	1.31	-0.71	0.02	-1.32
8.08	3.12	-0.22	1.28	-0.71	0.02	-1.33
8.10	3.08	-0.22	1.26	-0.71	0.02	-1.33
8.12	3.04	-0.22	1.24	-0.71	0.02	-1.33
8.13	3.06	-0.22	1.22	-0.71	0.02	-1.33
8.15	3.03	-0.22	1.20	-0.71	0.02	-1.32
8.17	3.01	-0.23	1.18	-0.71	0.02	-1.32
8.18	3.11	-0.23	1.15	-0.72	0.02	-1.33
8.20	3.14	-0.23	1.13	-0.72	0.02	-1.33
8.22	3.11	-0.23	1.11	-0.72	0.02	-1.33
8.23	3.04	-0.23	1.09	-0.72	0.02	-1.33
8.25	2.95	-0.23	1.07	-0.72	0.02	-1.33
8.27	2.89	-0.23	1.06	-0.72	0.02	-1.33
8.28	2.92	-0.23	1.04	-0.72	0.02	-1.33
8.30	3.04	-0.23	1.02	-0.72	0.02	-1.34
8.32	3.14	-0.23	1.00	-0.72	0.02	-1.34
8.33	3.06	-0.23	0.98	-0.72	0.02	-1.34
8.35	2.95	-0.23	0.97	-0.73	0.02	-1.34
8.37	2.87	-0.23	0.95	-0.73	0.02	-1.34
8.38	3.01	-0.23	0.94	-0.73	0.02	-1.34
8.40	3.11	-0.23	0.92	-0.73	0.02	-1.34
8.42	3.06	-0.23	0.90	-0.73	0.02	-1.34
8.43	3.00	-0.23	0.89	-0.73	0.02	-1.34
8.45	2.86	-0.23	0.87	-0.73	0.02	-1.34
8.47	2.86	-0.23	0.85	-0.74	0.02	-1.35
8.48	2.98	-0.23	0.84	-0.74	0.02	-1.35
8.50	2.95	-0.23	0.83	-0.74	0.02	-1.35
8.52	2.86	-0.23	0.81	-0.74	0.02	-1.35
8.53	2.91	-0.23	0.80	-0.74	0.02	-1.35
8.55	2.98	-0.23	0.78	-0.74	0.02	-1.35
8.57	3.01	-0.23	0.77	-0.73	0.02	-1.35
8.58	3.01	-0.23	0.75	-0.74	0.02	-1.35
8.60	2.97	-0.23	0.74	-0.74	0.02	-1.35

8.62	3.03	-0.23	0.73	-0.74	0.02	-1.35
8.63	3.17	-0.23	0.72	-0.74	0.02	-1.35
8.65	3.21	-0.24	0.71	-0.74	0.02	-1.35
8.67	3.18	-0.24	0.69	-0.74	0.02	-1.35
8.68	3.08	-0.24	0.68	-0.74	0.02	-1.36
8.70	2.94	-0.24	0.67	-0.74	0.02	-1.36
8.72	2.97	-0.24	0.66	-0.75	0.02	-1.36
8.73	3.14	-0.24	0.65	-0.75	0.02	-1.37
8.75	3.15	-0.24	0.64	-0.75	0.02	-1.37
8.77	3.21	-0.24	0.63	-0.75	0.02	-1.37
8.78	3.21	-0.24	0.62	-0.75	0.02	-1.35
8.80	3.08	-0.24	0.61	-0.75	0.02	-1.35
8.82	2.97	-0.24	0.60	-0.75	0.02	-1.37
8.83	2.95	-0.24	0.58	-0.75	0.02	-1.37
8.85	2.97	-0.24	0.57	-0.75	0.02	-1.37
8.87	2.92	-0.24	0.56	-0.76	0.02	-1.37
8.88	2.92	-0.24	0.55	-0.76	0.02	-1.37
8.90	3.04	-0.24	0.55	-0.76	0.02	-1.38
8.92	3.15	-0.24	0.54	-0.76	0.02	-1.38
8.93	3.20	-0.25	0.53	-0.76	0.02	-1.37
8.95	3.25	-0.25	0.52	-0.77	0.02	-1.37
8.97	3.32	-0.24	0.51	-0.77	0.02	-1.37
8.98	3.32	-0.24	0.50	-0.77	0.02	-1.38
9.00	3.31	-0.24	0.49	-0.77	0.02	-1.37
9.02	3.32	-0.24	0.49	-0.77	0.02	-1.37
9.03	3.31	-0.24	0.48	-0.77	0.02	-1.38
9.05	3.26	-0.25	0.47	-0.77	0.02	-1.38
9.07	3.17	-0.24	0.46	-0.77	0.02	-1.38
9.08	3.14	-0.24	0.46	-0.77	0.02	-1.38
9.10	3.03	-0.24	0.45	-0.78	0.02	-1.38
9.12	2.92	-0.24	0.44	-0.78	0.02	-1.38
9.13	3.00	-0.24	0.43	-0.78	0.02	-1.38
9.15	3.12	-0.25	0.42	-0.78	0.01	-1.38
9.17	3.08	-0.25	0.42	-0.78	0.02	-1.38
9.18	3.03	-0.25	0.41	-0.77	0.02	-1.38
9.20	3.09	-0.25	0.40	-0.78	0.02	-1.39
9.22	3.09	-0.25	0.40	-0.78	0.02	-1.39
9.23	3.15	-0.25	0.39	-0.78	0.02	-1.39
9.25	3.21	-0.25	0.39	-0.78	0.02	-1.39
9.27	3.21	-0.25	0.38	-0.78	0.02	-1.39
9.28	3.17	-0.25	0.37	-0.79	0.02	-1.39
9.30	3.08	-0.25	0.37	-0.79	0.02	-1.39
9.32	3.12	-0.25	0.36	-0.79	0.02	-1.40
9.33	3.21	-0.25	0.35	-0.79	0.02	-1.40
9.35	3.17	-0.25	0.35	-0.78	0.02	-1.40
9.37	3.17	-0.25	0.34	-0.78	0.02	-1.39
9.38	3.23	-0.25	0.34	-0.79	0.02	-1.39
9.40	3.17	-0.25	0.33	-0.79	0.02	-1.40
9.42	3.06	-0.25	0.33	-0.79	0.02	-1.40
9.43	3.06	-0.25	0.32	-0.79	0.02	-1.41
9.45	3.12	-0.25	0.32	-0.79	0.02	-1.40
9.47	3.20	-0.25	0.31	-0.79	0.02	-1.39
9.48	3.29	-0.25	0.31	-0.79	0.02	-1.40
9.50	3.38	-0.25	0.30	-0.80	0.02	-1.40
9.52	3.37	-0.25	0.29	-0.80	0.02	-1.41
9.53	3.20	-0.25	0.29	-0.80	0.02	-1.41
9.55	3.14	-0.25	0.28	-0.80	0.02	-1.41
9.57	3.21	-0.25	0.28	-0.80	0.02	-1.40
9.58	3.28	-0.26	0.28	-0.80	0.02	-1.40
9.60	3.23	-0.25	0.27	-0.80	0.02	-1.41
9.62	3.20	-0.25	0.27	-0.81	0.02	-1.41
9.63	3.21	-0.25	0.26	-0.81	0.02	-1.41
9.65	3.15	-0.25	0.26	-0.81	0.02	-1.41
9.67	3.06	-0.25	0.26	-0.81	0.02	-1.41
9.68	3.11	-0.25	0.25	-0.81	0.02	-1.41
9.70	3.20	-0.25	0.25	-0.81	0.02	-1.41
9.72	3.23	-0.25	0.24	-0.81	0.02	-1.41
9.73	3.34	-0.25	0.24	-0.82	0.02	-1.42
9.75	3.32	-0.25	0.23	-0.82	0.02	-1.42

9.77	3.11	-0.25	0.23	-0.82	0.02	-1.42
9.78	3.01	-0.26	0.22	-0.81	0.02	-1.42
9.80	3.11	-0.26	0.21	-0.82	0.02	-1.42
9.82	3.21	-0.26	0.21	-0.82	0.02	-1.42
9.83	3.23	-0.26	0.21	-0.83	0.02	-1.42
9.85	3.14	-0.26	0.21	-0.83	0.01	-1.42
9.87	3.15	-0.26	0.21	-0.83	0.01	-1.42
9.88	3.31	-0.26	0.20	-0.83	0.01	-1.43
9.90	3.28	-0.26	0.20	-0.83	0.01	-1.43
9.92	3.17	-0.26	0.20	-0.83	0.01	-1.42
9.93	3.29	-0.26	0.19	-0.83	0.01	-1.42
9.95	3.37	-0.26	0.19	-0.83	0.01	-1.42
9.97	3.34	-0.25	0.19	-0.83	0.01	-1.43
9.98	3.32	-0.25	0.18	-0.84	0.01	-1.43
10.00	3.32	-0.26	0.18	-0.84	0.02	-1.43
10.02	3.25	-0.26	0.18	-0.83	0.02	-1.43
10.03	3.25	-0.26	0.17	-0.84	0.01	-1.44
10.05	3.21	-0.26	0.17	-0.84	0.01	-1.44
10.07	3.17	-0.26	0.17	-0.84	0.01	-1.44
10.08	3.23	-0.26	0.17	-0.84	0.01	-1.44
10.10	3.18	-0.26	0.16	-0.84	0.01	-1.44
10.12	3.17	-0.26	0.16	-0.84	0.02	-1.44
10.13	3.32	-0.26	0.15	-0.85	0.02	-1.44
10.15	3.32	-0.27	0.15	-0.85	0.01	-1.44
10.17	3.14	-0.27	0.15	-0.85	0.01	-1.44
10.18	3.06	-0.26	0.14	-0.85	0.01	-1.44
10.20	3.15	-0.26	0.14	-0.85	0.01	-1.44
10.22	3.31	-0.26	0.14	-0.85	0.01	-1.44
10.23	3.35	-0.26	0.14	-0.86	0.01	-1.44
10.25	3.29	-0.26	0.14	-0.86	0.01	-1.44
10.27	3.25	-0.26	0.13	-0.86	0.01	-1.45
10.28	3.23	-0.26	0.13	-0.86	0.01	-1.44
10.30	3.31	-0.26	0.13	-0.86	0.01	-1.44
10.32	3.37	-0.26	0.13	-0.86	0.01	-1.45
10.33	3.43	-0.26	0.12	-0.87	0.01	-1.45
10.35	3.43	-0.26	0.12	-0.87	0.01	-1.45
10.37	3.34	-0.26	0.12	-0.87	0.01	-1.45
10.38	3.28	-0.26	0.12	-0.87	0.01	-1.45
10.40	3.29	-0.26	0.11	-0.88	0.01	-1.44
10.42	3.31	-0.26	0.11	-0.88	0.01	-1.45
10.43	3.32	-0.27	0.11	-0.88	0.01	-1.45
10.45	3.46	-0.27	0.11	-0.88	0.01	-1.45
10.47	3.43	-0.27	0.11	-0.88	0.01	-1.46
10.48	3.31	-0.27	0.10	-0.88	0.01	-1.46
10.50	3.35	-0.26	0.10	-0.89	0.01	-1.46
10.52	3.32	-0.27	0.10	-0.89	0.01	-1.46
10.53	3.26	-0.27	0.10	-0.89	0.01	-1.45
10.55	3.37	-0.27	0.10	-0.89	0.01	-1.45
10.57	3.48	-0.27	0.09	-0.89	0.01	-1.46
10.58	3.38	-0.27	0.09	-0.89	0.01	-1.46
10.60	3.28	-0.27	0.09	-0.89	0.01	-1.46
10.62	3.38	-0.26	0.08	-0.89	0.01	-1.46
10.63	3.49	-0.26	0.09	-0.90	0.01	-1.46
10.65	3.48	-0.26	0.08	-0.90	0.01	-1.46
10.67	3.48	-0.26	0.08	-0.90	0.01	-1.45
10.68	3.45	-0.27	0.08	-0.90	0.01	-1.45
10.70	3.38	-0.27	0.08	-0.90	0.01	-1.46
10.72	3.42	-0.27	0.07	-0.90	0.01	-1.46
10.73	3.48	-0.27	0.07	-0.91	0.01	-1.46
10.75	3.42	-0.27	0.07	-0.91	0.01	-1.46
10.77	3.35	-0.27	0.07	-0.91	0.01	-1.47
10.78	3.43	-0.27	0.07	-0.91	0.01	-1.47
10.80	3.52	-0.27	0.07	-0.91	0.01	-1.46
10.82	3.46	-0.27	0.07	-0.91	0.01	-1.46
10.83	3.31	-0.27	0.06	-0.91	0.01	-1.46
10.85	3.21	-0.27	0.06	-0.91	0.01	-1.46
10.87	3.25	-0.27	0.06	-0.91	0.01	-1.47
10.88	3.31	-0.27	0.06	-0.91	0.01	-1.47
10.90	3.26	-0.27	0.06	-0.91	0.01	-1.47

10.92	3.17	-0.27	0.06	-0.91	0.01	-1.47
10.93	3.12	-0.27	0.05	-0.91	0.01	-1.47
10.95	3.23	-0.27	0.05	-0.91	0.01	-1.47
10.97	3.26	-0.27	0.05	-0.91	0.01	-1.47
10.98	3.28	-0.28	0.05	-0.91	0.01	-1.48
11.00	3.40	-0.28	0.05	-0.91	0.01	-1.47
11.02	3.45	-0.28	0.05	-0.91	0.01	-1.47
11.03	3.54	-0.27	0.05	-0.91	0.01	-1.48
11.05	3.59	-0.27	0.05	-0.90	0.01	-1.48
11.07	3.51	-0.28	0.04	-0.90	0.01	-1.48
11.08	3.40	-0.28	0.04	-0.89	0.01	-1.48
11.10	3.38	-0.27	0.04	-0.88	0.01	-1.48
11.12	3.32	-0.27	0.04	-0.87	0.01	-1.48
11.13	3.28	-0.27	0.04	-0.86	0.01	-1.48
11.15	3.35	-0.27	0.04	-0.86	0.01	-1.48
11.17	3.37	-0.27	0.04	-0.85	0.01	-1.48
11.18	3.32	-0.27	0.04	-0.83	0.01	-1.48
11.20	3.31	-0.27	0.04	-0.82	0.01	-1.48
11.22	3.29	-0.27	0.03	-0.80	0.01	-1.47
11.23	3.34	-0.28	0.03	-0.79	0.02	-1.48
11.25	3.42	-0.28	0.03	-0.77	0.02	-1.48
11.27	3.49	-0.28	0.03	-0.76	0.01	-1.48
11.28	3.54	-0.28	0.03	-0.75	0.01	-1.48
11.30	3.46	-0.28	0.03	-0.75	0.01	-1.49
11.32	3.37	-0.28	0.03	-0.74	0.01	-1.49
11.33	3.31	-0.28	0.03	-0.73	0.01	-1.48
11.35	3.32	-0.28	0.02	-0.73	0.01	-1.48
11.37	3.37	-0.28	0.02	-0.73	0.01	-1.47
11.38	3.32	-0.28	0.02	-0.73	0.01	-1.47
11.40	3.37	-0.28	0.02	-0.73	0.01	-1.48
11.42	3.42	-0.28	0.02	-0.73	0.01	-1.49
11.43	3.37	-0.28	0.02	-0.73	0.01	-1.49
11.45	3.34	-0.28	0.02	-0.73	0.01	-1.49
11.47	3.38	-0.28	0.02	-0.73	0.01	-1.49
11.48	3.48	-0.28	0.02	-0.73	0.01	-1.49
11.50	3.48	-0.28	0.02	-0.72	0.01	-1.49
11.52	3.45	-0.28	0.02	-0.72	0.01	-1.48
11.53	3.51	-0.28	0.02	-0.73	0.01	-1.49
11.55	3.54	-0.28	0.01	-0.73	0.01	-1.50
11.57	3.51	-0.28	0.01	-0.73	0.01	-1.49
11.58	3.42	-0.28	0.01	-0.74	0.01	-1.49
11.60	3.37	-0.28	0.01	-0.74	0.01	-1.48
11.62	3.42	-0.28	0.01	-0.74	0.01	-1.48
11.63	3.51	-0.28	0.01	-0.75	0.01	-1.48
11.65	3.48	-0.28	0.01	-0.75	0.01	-1.48
11.67	3.49	-0.28	0.01	-0.75	0.01	-1.48
11.68	3.60	-0.28	0.00	-0.75	0.01	-1.49
11.70	3.55	-0.29	0.00	-0.76	0.01	-1.50
11.72	3.43	-0.28	0.01	-0.75	0.01	-1.49
11.73	3.52	-0.28	0.01	-0.76	0.01	-1.49
11.75	3.65	-0.28	0.01	-0.76	0.01	-1.49
11.77	3.55	-0.28	0.01	-0.76	0.01	-1.49
11.78	3.45	-0.28	0.01	-0.76	0.01	-1.50
11.80	3.48	-0.29	0.01	-0.76	0.01	-1.50
11.82	3.54	-0.28	0.01	-0.76	0.01	-1.50
11.83	3.60	-0.28	0.00	-0.76	0.01	-1.49
11.85	3.59	-0.29	0.00	-0.75	0.01	-1.49
11.87	3.54	-0.29	0.00	-0.75	0.01	-1.49
11.88	3.38	-0.28	0.00	-0.75	0.01	-1.50
11.90	3.34	-0.28	0.00	-0.75	0.01	-1.50
11.92	3.51	-0.28	0.00	-0.75	0.01	-1.50
11.93	3.55	-0.28	0.00	-0.75	0.01	-1.51
11.95	3.45	-0.28	0.00	-0.75	0.01	-1.50
11.97	3.45	-0.28	0.00	-0.74	0.01	-1.50
11.98	3.51	-0.28	0.00	-0.74	0.01	-1.50
12.00	3.48	-0.28	0.00	-0.74	0.01	-1.50
12.02	3.38	-0.28	0.00	-0.74	0.01	-1.50
12.03	3.51	-0.28	0.00	-0.74	0.01	-1.50
12.05	3.79	-0.28	0.00	-0.74	0.01	-1.50

12.07	3.88	-0.28	0.00	-0.74	0.01	-1.50
12.08	3.76	-0.29	0.00	-0.75	0.01	-1.50
12.10	3.69	-0.29	0.00	-0.75	0.01	-1.50
12.12	3.62	-0.29	0.00	-0.75	0.01	-1.50
12.13	3.38	-0.28	0.00	-0.75	0.01	-1.50
12.15	3.35	-0.28	0.00	-0.75	0.01	-1.50
12.17	3.55	-0.28	0.00	-0.75	0.01	-1.50
12.18	3.65	-0.28	0.00	-0.75	0.01	-1.50
12.20	3.62	-0.28	0.00	-0.75	0.01	-1.50
12.22	3.62	-0.28	0.00	-0.75	0.01	-1.51
12.23	3.60	-0.28	0.00	-0.76	0.01	-1.51
12.25	3.49	-0.28	0.00	-0.76	0.01	-1.51
12.27	3.43	-0.28	0.00	-0.77	0.01	-1.51
12.28	3.49	-0.28	-0.01	-0.77	0.01	-1.51
12.30	3.59	-0.28	-0.01	-0.77	0.01	-1.51
12.32	3.60	-0.28	-0.01	-0.77	0.01	-1.51
12.33	3.59	-0.28	0.00	-0.78	0.01	-1.51
12.35	3.57	-0.29	0.00	-0.78	0.01	-1.51
12.37	3.51	-0.29	0.00	-0.78	0.01	-1.52
12.38	3.40	-0.29	0.00	-0.79	0.01	-1.52
12.40	3.45	-0.29	0.00	-0.79	0.01	-1.51
12.42	3.65	-0.28	0.00	-0.79	0.01	-1.51
12.43	3.71	-0.28	0.00	-0.80	0.01	-1.51
12.45	3.59	-0.28	0.00	-0.80	0.01	-1.50
12.47	3.51	-0.28	0.00	-0.80	0.01	-1.50
12.48	3.60	-0.29	0.00	-0.80	0.01	-1.50
12.50	3.60	-0.29	0.00	-0.80	0.01	-1.51
12.52	3.57	-0.29	0.00	-0.81	0.01	-1.51
12.53	3.62	-0.29	0.00	-0.81	0.01	-1.51
12.55	3.59	-0.28	0.00	-0.82	0.01	-1.50
12.57	3.49	-0.28	0.00	-0.82	0.01	-1.50
12.58	3.57	-0.29	0.00	-0.81	0.01	-1.51
12.60	3.71	-0.29	0.00	-0.81	0.01	-1.51
12.62	3.69	-0.28	0.00	-0.82	0.01	-1.51
12.63	3.59	-0.29	0.00	-0.82	0.01	-1.50
12.65	3.66	-0.29	0.00	-0.83	0.01	-1.50
12.67	3.82	-0.29	0.00	-0.83	0.01	-1.50
12.68	3.74	-0.28	0.00	-0.83	0.01	-1.51
12.70	3.62	-0.28	0.01	-0.83	0.01	-1.51
12.72	3.65	-0.29	0.01	-0.84	0.01	-1.52
12.73	3.68	-0.29	0.01	-0.84	0.01	-1.51
12.75	3.71	-0.29	0.01	-0.84	0.01	-1.51
12.77	3.76	-0.29	0.01	-0.85	0.01	-1.51
12.78	3.76	-0.28	0.01	-0.85	0.01	-1.51
12.80	3.72	-0.29	0.01	-0.85	0.01	-1.51
12.82	3.72	-0.29	0.01	-0.85	0.01	-1.51
12.83	3.69	-0.29	0.01	-0.86	0.01	-1.51
12.85	3.60	-0.29	0.01	-0.86	0.01	-1.51
12.87	3.51	-0.29	0.01	-0.86	0.01	-1.51
12.88	3.49	-0.29	0.01	-0.86	0.01	-1.51
12.90	3.62	-0.29	0.01	-0.86	0.01	-1.51
12.92	3.68	-0.29	0.01	-0.87	0.01	-1.52
12.93	3.71	-0.29	0.02	-0.87	0.01	-1.52
12.95	3.72	-0.29	0.02	-0.87	0.01	-1.52
12.97	3.63	-0.29	0.02	-0.88	0.01	-1.52
12.98	3.49	-0.29	0.02	-0.88	0.01	-1.51
13.00	3.51	-0.29	0.02	-0.88	0.01	-1.51
13.02	3.69	-0.29	0.02	-0.88	0.01	-1.52
13.03	3.76	-0.29	0.02	-0.89	0.01	-1.52
13.05	3.71	-0.29	0.02	-0.89	0.01	-1.52
13.07	3.69	-0.29	0.03	-0.89	0.01	-1.52
13.08	3.63	-0.29	0.03	-0.89	0.01	-1.51
13.10	3.62	-0.29	0.03	-0.90	0.01	-1.52
13.12	3.65	-0.29	0.03	-0.90	0.01	-1.51
13.13	3.62	-0.29	0.03	-0.91	0.02	-1.51
13.15	3.62	-0.29	0.03	-0.91	0.02	-1.52
13.17	3.72	-0.28	0.03	-0.91	0.02	-1.52
13.18	3.80	-0.29	0.03	-0.92	0.01	-1.52
13.20	3.79	-0.29	0.04	-0.92	0.02	-1.52

13.22	3.79	-0.29	0.04	-0.92	0.01	-1.52
13.23	3.80	-0.29	0.04	-0.92	0.01	-1.52
13.25	3.82	-0.29	0.04	-0.93	0.01	-1.52
13.27	3.74	-0.29	0.04	-0.94	0.01	-1.52
13.28	3.60	-0.29	0.05	-0.94	0.01	-1.52
13.30	3.49	-0.29	0.05	-0.94	0.01	-1.51
13.32	3.65	-0.29	0.05	-0.94	0.01	-1.51
13.33	3.79	-0.29	0.05	-0.95	0.01	-1.52
13.35	3.79	-0.29	0.05	-0.95	0.01	-1.53
13.37	3.82	-0.29	0.05	-0.95	0.01	-1.53
13.38	3.88	-0.29	0.05	-0.95	0.01	-1.52
13.40	3.86	-0.29	0.05	-0.95	0.01	-1.51
13.42	3.79	-0.29	0.06	-0.96	0.01	-1.52
13.43	3.68	-0.29	0.06	-0.96	0.01	-1.52
13.45	3.54	-0.29	0.06	-0.97	0.01	-1.53
13.47	3.54	-0.29	0.06	-0.97	0.01	-1.52
13.48	3.69	-0.29	0.06	-0.97	0.01	-1.52
13.50	3.83	-0.29	0.06	-0.97	0.01	-1.52
13.52	3.85	-0.29	0.06	-0.97	0.01	-1.52
13.53	3.79	-0.29	0.06	-0.98	0.01	-1.52
13.55	3.80	-0.29	0.06	-0.98	0.01	-1.52
13.57	3.94	-0.28	0.07	-0.98	0.01	-1.52
13.58	4.02	-0.29	0.07	-0.97	0.01	-1.53
13.60	4.00	-0.29	0.07	-0.98	0.01	-1.53
13.62	4.02	-0.29	0.07	-0.99	0.01	-1.52
13.63	3.97	-0.29	0.07	-0.99	0.01	-1.53
13.65	3.97	-0.29	0.07	-0.99	0.01	-1.53
13.67	4.02	-0.29	0.07	-0.99	0.01	-1.52
13.68	4.00	-0.29	0.08	-0.99	0.01	-1.52
13.70	3.99	-0.29	0.08	-1.00	0.01	-1.52
13.72	3.91	-0.29	0.08	-1.00	0.01	-1.53
13.73	3.83	-0.30	0.08	-1.00	0.01	-1.52
13.75	3.82	-0.29	0.08	-1.00	0.01	-1.52
13.77	3.82	-0.29	0.08	-1.00	0.01	-1.52
13.78	3.93	-0.29	0.08	-1.00	0.01	-1.52
13.80	4.00	-0.29	0.08	-0.99	0.01	-1.52
13.82	3.94	-0.29	0.09	-1.00	0.01	-1.52
13.83	3.86	-0.29	0.09	-1.00	0.01	-1.52
13.85	3.97	-0.29	0.09	-1.00	0.01	-1.52
13.87	3.99	-0.29	0.09	-1.00	0.01	-1.53
13.88	3.85	-0.29	0.09	-1.00	0.01	-1.53
13.90	3.85	-0.29	0.09	-1.01	0.01	-1.53
13.92	3.86	-0.29	0.09	-1.01	0.01	-1.53
13.93	3.79	-0.29	0.09	-1.01	0.01	-1.53
13.95	3.74	-0.29	0.09	-1.01	0.01	-1.52
13.97	3.83	-0.29	0.09	-1.01	0.01	-1.52
13.98	3.91	-0.29	0.09	-1.01	0.01	-1.52
14.00	4.05	-0.29	0.09	-1.01	0.01	-1.52
14.02	4.06	-0.29	0.09	-1.02	0.01	-1.53
14.03	4.03	-0.29	0.09	-1.01	0.01	-1.52
14.05	4.00	-0.29	0.10	-1.01	0.01	-1.52
14.07	4.03	-0.29	0.10	-1.01	0.01	-1.53
14.08	4.06	-0.30	0.10	-1.02	0.01	-1.54
14.10	4.00	-0.30	0.09	-1.02	0.01	-1.54
14.12	3.88	-0.29	0.10	-1.01	0.01	-1.53
14.13	3.79	-0.29	0.10	-1.02	0.01	-1.52
14.15	3.85	-0.29	0.10	-1.02	0.02	-1.52
14.17	4.00	-0.29	0.10	-1.02	0.01	-1.53
14.18	3.99	-0.29	0.09	-1.02	0.01	-1.54
14.20	3.89	-0.30	0.10	-1.02	0.01	-1.54
14.22	3.94	-0.30	0.10	-1.02	0.01	-1.53
14.23	4.00	-0.30	0.10	-1.02	0.01	-1.53
14.25	3.91	-0.29	0.10	-1.02	0.01	-1.53
14.27	3.99	-0.29	0.10	-1.02	0.01	-1.53
14.28	4.16	-0.29	0.10	-1.02	0.01	-1.53
14.30	4.23	-0.29	0.10	-1.02	0.01	-1.53
14.32	4.23	-0.29	0.10	-1.02	0.01	-1.53
14.33	4.20	-0.29	0.10	-1.02	0.01	-1.53
14.35	4.11	-0.29	0.10	-1.03	0.01	-1.53

14.37	4.05	-0.29	0.10	-1.03	0.01	-1.53
14.38	4.08	-0.29	0.10	-1.03	0.01	-1.53
14.40	4.08	-0.29	0.09	-1.03	0.01	-1.54
14.42	4.05	-0.29	0.09	-1.03	0.01	-1.54
14.43	4.06	-0.29	0.09	-1.02	0.01	-1.53
14.45	4.14	-0.29	0.09	-1.03	0.01	-1.53
14.47	4.17	-0.29	0.09	-1.03	0.01	-1.53
14.48	4.20	-0.29	0.09	-1.03	0.01	-1.53
14.50	4.23	-0.30	0.09	-1.03	0.01	-1.54
14.52	4.30	-0.30	0.09	-1.03	0.01	-1.54
14.53	4.36	-0.30	0.09	-1.03	0.01	-1.53
14.55	4.30	-0.30	0.09	-1.03	0.01	-1.52
14.57	4.16	-0.30	0.09	-1.03	0.01	-1.52
14.58	4.02	-0.29	0.09	-1.03	0.01	-1.53
14.60	4.05	-0.30	0.09	-1.03	0.01	-1.53
14.62	4.16	-0.30	0.09	-1.03	0.01	-1.54
14.63	4.10	-0.30	0.09	-1.03	0.01	-1.55
14.65	3.96	-0.29	0.09	-1.03	0.01	-1.54
14.67	4.02	-0.29	0.09	-1.03	0.01	-1.53
14.68	4.16	-0.30	0.08	-1.03	0.01	-1.53
14.70	4.19	-0.30	0.08	-1.03	0.01	-1.54
14.72	4.25	-0.30	0.08	-1.03	0.01	-1.54
14.73	4.25	-0.30	0.08	-1.03	0.01	-1.54
14.75	4.19	-0.30	0.08	-1.03	0.01	-1.54
14.77	4.19	-0.29	0.08	-1.03	0.01	-1.54
14.78	4.17	-0.29	0.08	-1.03	0.01	-1.54
14.80	4.06	-0.29	0.08	-1.03	0.01	-1.54
14.82	3.99	-0.29	0.08	-1.03	0.01	-1.54
14.83	4.08	-0.30	0.07	-1.03	0.01	-1.54
14.85	4.25	-0.30	0.07	-1.03	0.01	-1.54
14.87	4.31	-0.29	0.07	-1.03	0.01	-1.54
14.88	4.25	-0.29	0.07	-1.03	0.01	-1.54
14.90	4.16	-0.30	0.07	-1.03	0.01	-1.54
14.92	4.11	-0.30	0.07	-1.03	0.01	-1.54
14.93	4.14	-0.29	0.07	-1.03	0.01	-1.54
14.95	4.23	-0.29	0.06	-1.03	0.01	-1.54
14.97	4.34	-0.29	0.06	-1.03	0.01	-1.54
14.98	4.47	-0.30	0.06	-1.03	0.01	-1.54
15.00	4.39	-0.30	0.06	-1.03	0.01	-1.55
15.02	4.28	-0.30	0.06	-1.03	0.01	-1.54
15.03	4.27	-0.30	0.06	-1.03	0.01	-1.54
15.05	4.25	-0.30	0.06	-1.03	0.01	-1.54
15.07	4.33	-0.30	0.06	-1.03	0.01	-1.53
15.08	4.42	-0.30	0.05	-1.03	0.01	-1.53
15.10	4.39	-0.30	0.05	-1.03	0.01	-1.54
15.12	4.40	-0.30	0.05	-1.03	0.01	-1.54
15.13	4.48	-0.30	0.05	-1.03	0.01	-1.54
15.15	4.44	-0.30	0.05	-1.02	0.01	-1.54
15.17	4.31	-0.30	0.05	-1.03	0.01	-1.54
15.18	4.31	-0.30	0.05	-1.02	0.01	-1.54
15.20	4.40	-0.30	0.04	-1.02	0.01	-1.54
15.22	4.47	-0.30	0.04	-1.01	0.01	-1.54
15.23	4.50	-0.30	0.04	-1.01	0.01	-1.55
15.25	4.61	-0.30	0.04	-1.01	0.01	-1.55
15.27	4.65	-0.30	0.04	-1.01	0.01	-1.54
15.28	4.65	-0.30	0.04	-1.01	0.01	-1.54
15.30	4.59	-0.30	0.04	-1.01	0.01	-1.54
15.32	4.47	-0.30	0.04	-1.01	0.01	-1.54
15.33	4.51	-0.30	0.04	-1.01	0.01	-1.55
15.35	4.61	-0.30	0.04	-1.01	0.01	-1.55
15.37	4.62	-0.30	0.04	-1.01	0.01	-1.55
15.38	4.61	-0.30	0.03	-1.01	0.01	-1.55
15.40	4.57	-0.29	0.03	-1.01	0.01	-1.54
15.42	4.56	-0.29	0.03	-1.00	0.01	-1.54
15.43	4.62	-0.29	0.03	-1.00	0.01	-1.54
15.45	4.56	-0.29	0.03	-1.00	0.01	-1.54
15.47	4.42	-0.30	0.03	-1.00	0.01	-1.55
15.48	4.42	-0.30	0.02	-1.00	0.01	-1.54
15.50	4.59	-0.29	0.03	-1.00	0.01	-1.54

15.52	4.70	-0.29	0.02	-1.00	0.01	-1.55
15.53	4.62	-0.30	0.02	-1.00	0.01	-1.54
15.55	4.62	-0.30	0.02	-1.00	0.01	-1.54
15.57	4.62	-0.30	0.02	-1.00	0.01	-1.55
15.58	4.57	-0.30	0.02	-0.99	0.01	-1.55
15.60	4.64	-0.30	0.02	-0.99	0.01	-1.55
15.62	4.70	-0.30	0.02	-0.99	0.01	-1.55
15.63	4.73	-0.30	0.01	-0.99	0.01	-1.55
15.65	4.68	-0.30	0.01	-0.99	0.01	-1.54
15.67	4.64	-0.29	0.02	-0.99	0.01	-1.55
15.68	4.70	-0.29	0.02	-0.99	0.01	-1.55
15.70	4.74	-0.29	0.01	-0.99	0.01	-1.55
15.72	4.70	-0.29	0.01	-0.99	0.01	-1.55
15.73	4.78	-0.30	0.01	-0.99	0.01	-1.55
15.75	4.76	-0.30	0.01	-0.99	0.01	-1.54
15.77	4.70	-0.30	0.01	-0.99	0.01	-1.54
15.78	4.67	-0.30	0.01	-0.99	0.01	-1.55
15.80	4.61	-0.30	0.01	-0.99	0.01	-1.55
15.82	4.68	-0.30	0.01	-0.98	0.01	-1.55
15.83	4.84	-0.30	0.00	-0.99	0.01	-1.55
15.85	4.87	-0.30	0.00	-0.99	0.01	-1.55
15.87	4.82	-0.30	0.00	-0.98	0.01	-1.55
15.88	4.79	-0.30	0.00	-0.98	0.01	-1.55
15.90	4.81	-0.30	0.00	-0.98	0.01	-1.55
15.92	4.87	-0.30	0.00	-0.98	0.01	-1.55
15.93	4.93	-0.29	0.00	-0.98	0.01	-1.55
15.95	4.91	-0.29	0.00	-0.99	0.01	-1.55
15.97	4.88	-0.30	0.00	-0.98	0.01	-1.55
15.98	4.84	-0.30	0.00	-0.98	0.01	-1.54
16.00	4.74	-0.30	-0.01	-0.99	0.01	-1.54
16.02	4.74	-0.30	-0.01	-0.99	0.01	-1.55
16.03	4.82	-0.30	-0.01	-0.99	0.02	-1.56
16.05	4.82	-0.30	-0.01	-0.98	0.01	-1.55
16.07	4.82	-0.30	-0.01	-0.98	0.01	-1.55
16.08	4.82	-0.30	-0.01	-0.98	0.02	-1.56
16.10	4.85	-0.30	-0.01	-0.98	0.02	-1.55
16.12	4.87	-0.29	-0.01	-0.98	0.02	-1.55
16.13	4.79	-0.30	-0.01	-0.98	0.02	-1.55
16.15	4.76	-0.30	-0.01	-0.98	0.02	-1.55
16.17	4.95	-0.30	-0.01	-0.98	0.02	-1.55
16.18	4.99	-0.30	-0.01	-0.98	0.02	-1.56
16.20	4.82	-0.30	-0.02	-0.98	0.02	-1.56
16.22	4.67	-0.30	-0.02	-0.98	0.02	-1.55
16.23	4.70	-0.30	-0.01	-0.98	0.02	-1.55
16.25	4.87	-0.30	-0.02	-0.98	0.02	-1.54
16.27	4.88	-0.30	-0.02	-0.98	0.02	-1.54
16.28	4.84	-0.30	-0.02	-0.98	0.02	-1.55
16.30	4.98	-0.30	-0.02	-0.98	0.02	-1.55
16.32	5.02	-0.30	-0.02	-0.98	0.02	-1.55
16.33	4.96	-0.30	-0.02	-0.99	0.02	-1.55
16.35	5.02	-0.30	-0.02	-0.99	0.02	-1.55
16.37	5.04	-0.30	-0.02	-0.99	0.02	-1.55
16.38	4.91	-0.30	-0.02	-0.99	0.02	-1.55
16.40	4.78	-0.30	-0.02	-0.99	0.02	-1.55
16.42	4.79	-0.30	-0.02	-0.99	0.02	-1.56
16.43	4.88	-0.30	-0.02	-0.99	0.02	-1.56
16.45	4.88	-0.29	-0.02	-0.99	0.02	-1.56
16.47	4.87	-0.29	-0.02	-0.99	0.02	-1.55
16.48	4.85	-0.30	-0.02	-0.99	0.02	-1.55
16.50	4.79	-0.30	-0.02	-0.99	0.02	-1.55
16.52	4.68	-0.30	-0.03	-0.99	0.02	-1.56
16.53	4.70	-0.30	-0.03	-0.99	0.02	-1.56
16.55	4.73	-0.30	-0.03	-1.00	0.02	-1.56
16.57	4.81	-0.30	-0.03	-1.00	0.02	-1.55
16.58	4.99	-0.30	-0.03	-1.00	0.02	-1.55
16.60	5.04	-0.30	-0.03	-1.00	0.02	-1.55
16.62	5.02	-0.30	-0.03	-1.00	0.02	-1.55
16.63	4.98	-0.30	-0.03	-1.00	0.02	-1.55
16.65	4.91	-0.30	-0.03	-1.00	0.02	-1.55

16.67	4.79	-0.30	-0.03	-1.00	0.02	-1.55
16.68	4.71	-0.30	-0.03	-1.00	0.02	-1.55
16.70	4.65	-0.30	-0.03	-1.00	0.02	-1.55
16.72	4.70	-0.30	-0.03	-1.00	0.02	-1.54
16.73	4.84	-0.30	-0.03	-1.00	0.02	-1.54
16.75	4.90	-0.30	-0.03	-1.00	0.02	-1.55
16.77	4.96	-0.30	-0.03	-1.00	0.02	-1.56
16.78	4.98	-0.30	-0.03	-1.00	0.02	-1.55
16.80	4.87	-0.30	-0.03	-1.01	0.02	-1.55
16.82	4.87	-0.30	-0.03	-1.01	0.02	-1.55
16.83	4.90	-0.30	-0.03	-1.01	0.02	-1.55
16.85	4.78	-0.30	-0.03	-1.01	0.02	-1.55
16.87	4.70	-0.30	-0.03	-1.01	0.02	-1.54
16.88	4.81	-0.30	-0.03	-1.01	0.02	-1.55
16.90	4.95	-0.30	-0.04	-1.01	0.02	-1.55
16.92	4.93	-0.30	-0.03	-1.01	0.02	-1.55
16.93	4.81	-0.30	-0.03	-1.01	0.02	-1.55
16.95	4.84	-0.31	-0.04	-1.01	0.02	-1.55
16.97	5.01	-0.30	-0.04	-1.01	0.02	-1.55
16.98	5.13	-0.30	-0.04	-1.01	0.02	-1.55
17.00	5.09	-0.30	-0.04	-1.01	0.02	-1.54
17.02	4.93	-0.30	-0.04	-1.01	0.02	-1.55
17.03	4.87	-0.30	-0.04	-1.01	0.02	-1.55
17.05	4.82	-0.30	-0.04	-1.02	0.02	-1.55
17.07	4.76	-0.30	-0.04	-1.02	0.02	-1.55
17.08	4.79	-0.30	-0.04	-1.02	0.02	-1.55
17.10	4.82	-0.30	-0.04	-1.02	0.02	-1.55
17.12	4.78	-0.30	-0.04	-1.02	0.02	-1.55
17.13	4.76	-0.30	-0.04	-1.03	0.02	-1.56
17.15	4.82	-0.30	-0.04	-1.03	0.02	-1.56
17.17	4.76	-0.30	-0.04	-1.02	0.02	-1.56
17.18	4.73	-0.30	-0.04	-1.03	0.02	-1.55
17.20	4.81	-0.30	-0.04	-1.03	0.02	-1.54
17.22	4.81	-0.30	-0.04	-1.03	0.02	-1.54
17.23	4.85	-0.30	-0.04	-1.04	0.02	-1.54
17.25	4.93	-0.30	-0.04	-1.04	0.02	-1.54
17.27	4.82	-0.30	-0.04	-1.03	0.02	-1.54
17.28	4.74	-0.30	-0.04	-1.03	0.02	-1.54
17.30	4.76	-0.30	-0.04	-1.03	0.02	-1.55
17.32	4.64	-0.30	-0.04	-1.04	0.02	-1.55
17.33	4.61	-0.30	-0.04	-1.04	0.02	-1.55
17.35	4.59	-0.30	-0.05	-1.04	0.02	-1.55
17.37	4.56	-0.30	-0.04	-1.04	0.02	-1.55
17.38	4.59	-0.30	-0.04	-1.04	0.02	-1.55
17.40	4.67	-0.30	-0.04	-1.04	0.02	-1.55
17.42	4.78	-0.30	-0.05	-1.04	0.02	-1.56
17.43	4.82	-0.30	-0.05	-1.05	0.02	-1.56
17.45	4.74	-0.31	-0.05	-1.05	0.02	-1.55
17.47	4.81	-0.31	-0.05	-1.05	0.02	-1.55
17.48	4.85	-0.30	-0.05	-1.05	0.02	-1.54
17.50	4.65	-0.31	-0.05	-1.05	0.02	-1.54
17.52	4.53	-0.31	-0.05	-1.05	0.02	-1.54
17.53	4.61	-0.30	-0.05	-1.05	0.02	-1.55
17.55	4.53	-0.30	-0.05	-1.05	0.02	-1.55
17.57	4.54	-0.30	-0.05	-1.05	0.02	-1.55
17.58	4.68	-0.30	-0.05	-1.05	0.02	-1.55
17.60	4.64	-0.30	-0.05	-1.06	0.02	-1.56
17.62	4.59	-0.30	-0.05	-1.06	0.02	-1.55
17.63	4.50	-0.30	-0.05	-1.06	0.02	-1.55
17.65	4.47	-0.31	-0.05	-1.06	0.02	-1.54
17.67	4.61	-0.31	-0.05	-1.06	0.01	-1.54
17.68	4.65	-0.31	-0.05	-1.06	0.02	-1.54
17.70	4.57	-0.30	-0.05	-1.06	0.02	-1.54
17.72	4.54	-0.30	-0.05	-1.06	0.02	-1.54
17.73	4.56	-0.31	-0.05	-1.06	0.02	-1.54
17.75	4.56	-0.31	-0.05	-1.07	0.02	-1.55
17.77	4.53	-0.31	-0.05	-1.07	0.02	-1.55
17.78	4.45	-0.31	-0.05	-1.07	0.02	-1.54
17.80	4.42	-0.30	-0.05	-1.07	0.02	-1.53

17.82	4.40	-0.30	-0.05	-1.07	0.02	-1.53
17.83	4.48	-0.31	-0.05	-1.07	0.02	-1.53
17.85	4.68	-0.31	-0.05	-1.07	0.02	-1.54
17.87	4.74	-0.31	-0.05	-1.08	0.02	-1.54
17.88	4.65	-0.31	-0.05	-1.08	0.02	-1.54
17.90	4.48	-0.31	-0.05	-1.08	0.02	-1.54
17.92	4.39	-0.30	-0.05	-1.08	0.02	-1.54
17.93	4.48	-0.30	-0.05	-1.08	0.02	-1.54
17.95	4.51	-0.30	-0.05	-1.08	0.02	-1.54
17.97	4.37	-0.30	-0.05	-1.08	0.02	-1.54
17.98	4.33	-0.30	-0.05	-1.09	0.02	-1.54
18.00	4.37	-0.31	-0.05	-1.09	0.02	-1.54
18.02	4.39	-0.31	-0.05	-1.09	0.02	-1.53
18.03	4.39	-0.31	-0.05	-1.09	0.02	-1.53
18.05	4.44	-0.30	-0.05	-1.09	0.02	-1.54
18.07	4.56	-0.30	-0.05	-1.09	0.02	-1.54
18.08	4.65	-0.30	-0.05	-1.09	0.02	-1.54
18.10	4.61	-0.30	-0.05	-1.09	0.02	-1.54
18.12	4.39	-0.30	-0.05	-1.10	0.02	-1.54
18.13	4.22	-0.31	-0.05	-1.09	0.02	-1.53
18.15	4.33	-0.31	-0.05	-1.10	0.02	-1.53
18.17	4.50	-0.30	-0.05	-1.10	0.02	-1.53
18.18	4.44	-0.30	-0.05	-1.10	0.02	-1.53
18.20	4.33	-0.30	-0.05	-1.10	0.02	-1.53
18.22	4.30	-0.30	-0.05	-1.10	0.02	-1.53
18.23	4.36	-0.31	-0.05	-1.10	0.02	-1.53
18.25	4.40	-0.31	-0.05	-1.10	0.02	-1.54
18.27	4.39	-0.31	-0.05	-1.10	0.02	-1.53
18.28	4.44	-0.31	-0.05	-1.10	0.02	-1.53
18.30	4.39	-0.31	-0.05	-1.10	0.02	-1.53
18.32	4.28	-0.31	-0.05	-1.10	0.02	-1.53
18.33	4.23	-0.30	-0.05	-1.10	0.02	-1.53
18.35	4.23	-0.30	-0.05	-1.10	0.02	-1.52
18.37	4.33	-0.30	-0.05	-1.10	0.02	-1.53
18.38	4.50	-0.30	-0.05	-1.11	0.02	-1.53
18.40	4.57	-0.30	-0.05	-1.11	0.02	-1.53
18.42	4.51	-0.30	-0.05	-1.11	0.02	-1.53
18.43	4.45	-0.31	-0.05	-1.11	0.02	-1.53
18.45	4.50	-0.31	-0.05	-1.11	0.02	-1.53
18.47	4.45	-0.31	-0.05	-1.11	0.02	-1.52
18.48	4.37	-0.31	-0.05	-1.11	0.02	-1.53
18.50	4.40	-0.31	-0.05	-1.11	0.02	-1.53
18.52	4.44	-0.31	-0.05	-1.11	0.02	-1.53
18.53	4.47	-0.30	-0.05	-1.12	0.02	-1.53
18.55	4.54	-0.30	-0.05	-1.12	0.02	-1.53
18.57	4.64	-0.30	-0.05	-1.12	0.02	-1.52
18.58	4.56	-0.30	-0.05	-1.12	0.02	-1.52
18.60	4.42	-0.31	-0.05	-1.12	0.02	-1.53
18.62	4.34	-0.31	-0.05	-1.12	0.02	-1.53
18.63	4.37	-0.30	-0.05	-1.12	0.02	-1.53
18.65	4.48	-0.30	-0.05	-1.12	0.02	-1.52
18.67	4.56	-0.31	-0.05	-1.12	0.02	-1.52
18.68	4.65	-0.31	-0.04	-1.12	0.02	-1.52
18.70	4.67	-0.31	-0.05	-1.12	0.02	-1.53
18.72	4.59	-0.31	-0.05	-1.12	0.02	-1.53
18.73	4.50	-0.31	-0.05	-1.12	0.02	-1.53
18.75	4.42	-0.30	-0.05	-1.12	0.02	-1.52
18.77	4.36	-0.30	-0.04	-1.12	0.02	-1.52
18.78	4.33	-0.30	-0.05	-1.13	0.02	-1.52
18.80	4.34	-0.31	-0.05	-1.13	0.02	-1.52
18.82	4.23	-0.31	-0.05	-1.13	0.02	-1.52
18.83	4.14	-0.31	-0.04	-1.13	0.02	-1.52
18.85	4.23	-0.31	-0.04	-1.12	0.02	-1.52
18.87	4.37	-0.30	-0.04	-1.12	0.02	-1.52
18.88	4.48	-0.30	-0.04	-1.13	0.02	-1.51
18.90	4.50	-0.31	-0.04	-1.13	0.02	-1.51
18.92	4.45	-0.31	-0.04	-1.13	0.02	-1.52
18.93	4.45	-0.31	-0.04	-1.13	0.02	-1.51
18.95	4.44	-0.31	-0.04	-1.13	0.02	-1.51

biomedicines MDPI

18.97	4.37	-0.31	-0.04	-1.13	0.02	-1.50
18.98	4.30	-0.31	-0.04	-1.13	0.02	-1.50
19.00	4.28	-0.31	-0.04	-1.13	0.02	-1.50
19.02	4.37	-0.31	-0.04	-1.13	0.02	-1.51
19.03	4.50	-0.31	-0.04	-1.14	0.02	-1.52
19.05	4.51	-0.31	-0.04	-1.14	0.02	-1.52
19.07	4.44	-0.31	-0.04	-1.14	0.02	-1.51
19.08	4.45	-0.31	-0.04	-1.14	0.02	-1.52
19.10	4.57	-0.31	-0.04	-1.14	0.02	-1.51
19.12	4.62	-0.31	-0.04	-1.14	0.02	-1.51
19.13	4.64	-0.31	-0.04	-1.14	0.02	-1.51
19.15	4.57	-0.30	-0.04	-1.14	0.02	-1.51
19.17	4.57	-0.31	-0.04	-1.14	0.01	-1.51
19.18	4.59	-0.31	-0.04	-1.14	0.01	-1.51
19.20	4.45	-0.31	-0.04	-1.14	0.02	-1.51
19.22	4.33	-0.31	-0.04	-1.14	0.02	-1.50
19.23	4.31	-0.31	-0.04	-1.14	0.02	-1.51
19.25	4.37	-0.31	-0.04	-1.14	0.02	-1.52
19.27	4.39	-0.31	-0.04	-1.14	0.02	-1.51
19.28	4.31	-0.31	-0.04	-1.14	0.02	-1.51
19.30	4.27	-0.31	-0.04	-1.15	0.02	-1.50
19.32	4.23	-0.31	-0.04	-1.15	0.02	-1.50
19.33	4.30	-0.31	-0.04	-1.15	0.02	-1.49
19.35	4.44	-0.31	-0.03	-1.14	0.01	-1.50
19.37	4.37	-0.31	-0.03	-1.15	0.01	-1.51
19.38	4.25	-0.31	-0.03	-1.15	0.01	-1.50
19.40	4.25	-0.31	-0.03	-1.15	0.01	-1.50
19.42	4.37	-0.30	-0.03	-1.15	0.01	-1.50
19.43	4.45	-0.30	-0.03	-1.15	0.01	-1.50
19.45	4.51	-0.31	-0.03	-1.15	0.02	-1.50
19.47	4.53	-0.31	-0.03	-1.15	0.02	-1.50
19.48	4.54	-0.31	-0.03	-1.15	0.02	-1.50
19.50	4.61	-0.31	-0.03	-1.15	0.01	-1.50
19.52	4.54	-0.31	-0.03	-1.15	0.01	-1.49
19.53	4.53	-0.31	-0.03	-1.15	0.02	-1.49
19.55	4.50	-0.31	-0.03	-1.15	0.01	-1.49
19.57	4.40	-0.31	-0.03	-1.15	0.01	-1.49
19.58	4.42	-0.31	-0.03	-1.15	0.02	-1.49
19.60	4.48	-0.31	-0.03	-1.15	0.02	-1.50
19.62	4.59	-0.31	-0.03	-1.15	0.02	-1.50
19.63	4.62	-0.31	-0.03	-1.15	0.02	-1.49
19.65	4.42	-0.31	-0.03	-1.16	0.02	-1.49
19.67	4.22	-0.31	-0.03	-1.16	0.01	-1.49
19.68	4.22	-0.31	-0.02	-1.15	0.01	-1.49
19.70	4.39	-0.31	-0.02	-1.15	0.01	-1.49
19.72	4.56	-0.30	-0.02	-1.15	0.01	-1.49
19.73	4.53	-0.31	-0.02	-1.16	0.02	-1.49
19.75	4.44	-0.31	-0.02	-1.16	0.02	-1.49
19.77	4.39	-0.31	-0.02	-1.16	0.02	-1.49
19.78	4.33	-0.30	-0.02	-1.15	0.01	-1.49
19.80	4.36	-0.31	-0.02	-1.16	0.01	-1.48
19.82	4.47	-0.31	-0.02	-1.16	0.01	-1.48
19.83	4.40	-0.31	-0.02	-1.16	0.01	-1.47
19.85	4.31	-0.31	-0.02	-1.16	0.01	-1.48
19.87	4.34	-0.31	-0.02	-1.16	0.01	-1.48
19.88	4.42	-0.31	-0.02	-1.16	0.01	-1.48
19.90	4.51	-0.31	-0.02	-1.16	0.01	-1.48
19.92	4.57	-0.31	-0.02	-1.16	0.02	-1.47
19.93	4.56	-0.31	-0.02	-1.16	0.02	-1.47
19.95	4.48	-0.31	-0.02	-1.16	0.02	-1.47
19.97	4.53	-0.31	-0.02	-1.16	0.02	-1.47
19.98	4.56	-0.31	-0.02	-1.17	0.01	-1.48
20.00	4.47	-0.31	-0.02	-1.17	0.01	-1.47
20.02	4.47	-0.31	-0.02	-1.16	0.01	-1.47
20.03	4.40	-0.31	-0.02	-1.17	0.01	-1.47
20.05	4.36	-0.30	-0.02	-1.17	0.01	-1.47
20.07	4.51	-0.31	-0.02	-1.17	0.01	-1.48
20.08	4.62	-0.31	-0.02	-1.16	0.02	-1.48
20.10	4.67	-0.31	-0.02	-1.16	0.01	-1.48

20.12	4.62	-0.31	-0.02	-1.16	0.01	-1.47
20.13	4.56	-0.31	-0.02	-1.16	0.01	-1.47
20.15	4.54	-0.31	-0.02	-1.17	0.02	-1.47
20.17	4.51	-0.31	-0.02	-1.17	0.01	-1.47
20.18	4.42	-0.31	-0.02	-1.17	0.01	-1.46
20.20	4.42	-0.31	-0.02	-1.17	0.01	-1.47
20.22	4.53	-0.31	-0.02	-1.17	0.01	-1.47
20.23	4.56	-0.31	-0.02	-1.17	0.01	-1.47
20.25	4.59	-0.31	-0.02	-1.17	0.01	-1.47
20.27	4.57	-0.31	-0.02	-1.17	0.01	-1.47
20.28	4.45	-0.31	-0.02	-1.17	0.01	-1.47
20.30	4.40	-0.31	-0.02	-1.17	0.01	-1.46
20.32	4.42	-0.31	-0.02	-1.16	0.01	-1.46
20.33	4.40	-0.31	-0.02	-1.16	0.02	-1.46
20.35	4.39	-0.31	-0.02	-1.16	0.02	-1.46
20.37	4.42	-0.31	-0.02	-1.17	0.02	-1.47
20.38	4.42	-0.31	-0.02	-1.17	0.01	-1.47
20.40	4.39	-0.31	-0.02	-1.17	0.01	-1.46
20.42	4.45	-0.31	-0.02	-1.17	0.01	-1.46
20.43	4.57	-0.31	-0.02	-1.17	0.01	-1.46
20.45	4.74	-0.31	-0.02	-1.17	0.01	-1.46
20.47	4.84	-0.31	-0.02	-1.17	0.01	-1.46
20.48	4.73	-0.31	-0.02	-1.18	0.01	-1.45
20.50	4.62	-0.31	-0.02	-1.17	0.01	-1.45
20.52	4.61	-0.31	-0.02	-1.17	0.02	-1.45
20.53	4.56	-0.31	-0.02	-1.17	0.02	-1.46
20.55	4.56	-0.31	-0.02	-1.17	0.02	-1.46
20.57	4.54	-0.31	-0.02	-1.17	0.01	-1.45
20.58	4.45	-0.31	-0.02	-1.17	0.01	-1.45
20.60	4.48	-0.31	-0.02	-1.17	0.02	-1.45
20.62	4.50	-0.31	-0.03	-1.17	0.02	-1.45
20.63	4.42	-0.31	-0.03	-1.17	0.02	-1.45
20.65	4.44	-0.31	-0.03	-1.17	0.02	-1.45
20.67	4.54	-0.31	-0.02	-1.17	0.02	-1.45
20.68	4.62	-0.31	-0.02	-1.17	0.02	-1.45
20.70	4.54	-0.31	-0.02	-1.17	0.03	-1.44
20.72	4.44	-0.31	-0.02	-1.17	0.03	-1.44
20.73	4.45	-0.30	-0.03	-1.17	0.03	-1.44
20.75	4.40	-0.30	-0.03	-1.17	0.03	-1.43
20.77	4.36	-0.31	-0.03	-1.17	0.04	-1.43
20.78	4.47	-0.31	-0.02	-1.18	0.04	-1.43
20.80	4.50	-0.30	-0.03	-1.17	0.05	-1.43
20.82	4.39	-0.30	-0.03	-1.17	0.06	-1.43
20.83	4.39	-0.30	-0.03	-1.17	0.06	-1.43
20.85	4.47	-0.30	-0.03	-1.17	0.07	-1.43
20.87	4.54	-0.31	-0.03	-1.17	0.08	-1.43
20.88	4.62	-0.31	-0.03	-1.18	0.09	-1.43
20.90	4.61	-0.31	-0.03	-1.18	0.10	-1.43
20.92	4.54	-0.31	-0.03	-1.18	0.11	-1.43
20.93	4.48	-0.31	-0.03	-1.17	0.12	-1.43
20.95	4.45	-0.31	-0.03	-1.17	0.14	-1.42
20.97	4.48	-0.31	-0.03	-1.17	0.16	-1.42
20.98	4.51	-0.31	-0.03	-1.18	0.18	-1.42
21.00	4.51	-0.31	-0.03	-1.18	0.20	-1.43
21.02	4.48	-0.31	-0.03	-1.18	0.23	-1.43
21.03	4.47	-0.31	-0.03	-1.18	0.26	-1.43
21.05	4.54	-0.31	-0.03	-1.18	0.29	-1.42
21.07	4.57	-0.31	-0.03	-1.18	0.33	-1.42
21.08	4.48	-0.31	-0.03	-1.18	0.37	-1.42
21.10	4.47	-0.31	-0.03	-1.18	0.42	-1.42
21.12	4.54	-0.31	-0.03	-1.17	0.48	-1.42
21.13	4.59	-0.31	-0.03	-1.17	0.54	-1.42
21.15	4.44	-0.31	-0.04	-1.17	0.61	-1.41
21.17	4.39	-0.31	-0.04	-1.17	0.69	-1.40
21.18	4.53	-0.31	-0.04	-1.18	0.77	-1.40
21.20	4.59	-0.31	-0.04	-1.18	0.87	-1.41
21.22	4.51	-0.31	-0.04	-1.18	0.98	-1.41
21.23	4.51	-0.31	-0.04	-1.17	1.10	-1.41
21.25	4.62	-0.31	-0.04	-1.17	1.23	-1.41

21.27	4.68	-0.31	-0.04	-1.17	1.38	-1.41
21.28	4.54	-0.31	-0.04	-1.17	1.54	-1.41
21.30	4.37	-0.31	-0.04	-1.17	1.72	-1.40
21.32	4.44	-0.31	-0.04	-1.17	1.92	-1.40
21.33	4.53	-0.31	-0.04	-1.17	2.13	-1.40
21.35	4.57	-0.31	-0.04	-1.17	2.36	-1.40
21.37	4.64	-0.31	-0.04	-1.18	2.62	-1.40
21.38	4.68	-0.31	-0.04	-1.18	2.89	-1.40
21.40	4.65	-0.31	-0.04	-1.18	3.18	-1.40
21.42	4.59	-0.31	-0.04	-1.17	3.50	-1.40
21.43	4.51	-0.31	-0.04	-1.17	3.84	-1.40
21.45	4.50	-0.31	-0.05	-1.17	4.20	-1.39
21.47	4.54	-0.31	-0.04	-1.17	4.59	-1.39
21.48	4.47	-0.31	-0.04	-1.17	5.01	-1.40
21.50	4.37	-0.31	-0.04	-1.17	5.45	-1.40
21.52	4.48	-0.31	-0.04	-1.17	5.92	-1.39
21.53	4.50	-0.31	-0.04	-1.17	6.41	-1.39
21.55	4.53	-0.31	-0.05	-1.17	6.92	-1.38
21.57	4.62	-0.31	-0.05	-1.17	7.46	-1.39
21.58	4.70	-0.31	-0.05	-1.17	8.03	-1.39
21.60	4.78	-0.31	-0.05	-1.17	8.63	-1.39
21.62	4.81	-0.32	-0.04	-1.17	9.25	-1.38
21.63	4.70	-0.31	-0.05	-1.17	9.90	-1.38
21.65	4.59	-0.31	-0.05	-1.17	10.58	-1.38
21.67	4.64	-0.31	-0.05	-1.17	11.27	-1.38
21.68	4.61	-0.31	-0.05	-1.17	11.99	-1.37
21.70	4.51	-0.31	-0.05	-1.17	12.74	-1.37
21.72	4.54	-0.31	-0.05	-1.17	13.51	-1.38
21.73	4.62	-0.31	-0.05	-1.17	14.32	-1.38
21.75	4.67	-0.31	-0.05	-1.17	15.15	-1.37
21.77	4.71	-0.31	-0.05	-1.16	16.00	-1.37
21.78	4.68	-0.31	-0.05	-1.16	16.88	-1.38
21.80	4.57	-0.31	-0.05	-1.16	17.78	-1.37
21.82	4.45	-0.31	-0.05	-1.16	18.70	-1.37
21.83	4.39	-0.31	-0.05	-1.16	19.64	-1.37
21.85	4.47	-0.31	-0.05	-1.17	20.59	-1.36
21.87	4.54	-0.30	-0.05	-1.17	21.57	-1.37
21.88	4.54	-0.31	-0.05	-1.17	22.57	-1.37
21.90	4.57	-0.31	-0.05	-1.16	23.59	-1.37
21.92	4.64	-0.31	-0.05	-1.16	24.63	-1.37
21.93	4.61	-0.31	-0.05	-1.16	25.69	-1.37
21.95	4.62	-0.31	-0.05	-1.16	26.76	-1.36
21.97	4.71	-0.31	-0.05	-1.17	27.85	-1.36
21.98	4.70	-0.31	-0.05	-1.17	28.94	-1.35
22.00	4.59	-0.31	-0.05	-1.17	30.05	-1.35
22.02	4.56	-0.31	-0.05	-1.17	31.16	-1.35
22.03	4.59	-0.31	-0.05	-1.16	32.28	-1.34
22.05	4.64	-0.31	-0.05	-1.16	33.41	-1.35
22.07	4.64	-0.31	-0.05	-1.16	34.56	-1.35
22.08	4.64	-0.31	-0.05	-1.17	35.71	-1.35
22.10	4.62	-0.31	-0.05	-1.16	36.86	-1.35
22.12	4.61	-0.31	-0.05	-1.16	38.02	-1.34
22.13	4.65	-0.31	-0.05	-1.16	39.16	-1.35
22.15	4.59	-0.31	-0.05	-1.16	40.31	-1.34
22.17	4.53	-0.31	-0.04	-1.16	41.46	-1.34
22.18	4.57	-0.31	-0.04	-1.15	42.61	-1.34
22.20	4.59	-0.30	-0.04	-1.16	43.76	-1.34
22.22	4.59	-0.30	-0.04	-1.16	44.92	-1.34
22.23	4.68	-0.31	-0.04	-1.16	46.07	-1.34
22.25	4.73	-0.30	-0.04	-1.16	47.22	-1.34
22.27	4.71	-0.30	-0.04	-1.15	48.37	-1.34
22.28	4.68	-0.31	-0.04	-1.15	49.51	-1.34
22.30	4.62	-0.30	-0.04	-1.15	50.64	-1.33
22.32	4.51	-0.30	-0.04	-1.15	51.77	-1.33
22.33	4.56	-0.30	-0.04	-1.15	52.90	-1.33
22.35	4.71	-0.30	-0.03	-1.15	54.02	-1.32
22.37	4.84	-0.30	-0.03	-1.16	55.15	-1.32
22.38	4.88	-0.30	-0.03	-1.15	56.26	-1.31
22.40	4.82	-0.30	-0.03	-1.15	57.37	-1.32

22.42	4.73	-0.30	-0.03	-1.14	58.48	-1.32
22.43	4.61	-0.30	-0.03	-1.14	59.58	-1.32
22.45	4.62	-0.30	-0.03	-1.14	60.67	-1.32
22.47	4.70	-0.30	-0.02	-1.14	61.75	-1.32
22.48	4.62	-0.30	-0.02	-1.14	62.82	-1.32
22.50	4.64	-0.30	-0.02	-1.14	63.89	-1.31
22.52	4.78	-0.30	-0.02	-1.13	64.96	-1.30
22.53	4.88	-0.30	-0.02	-1.13	66.01	-1.31
22.55	4.91	-0.30	-0.01	-1.13	67.07	-1.31
22.57	4.90	-0.30	-0.01	-1.13	68.11	-1.31
22.58	4.85	-0.29	-0.01	-1.13	69.15	-1.31
22.60	4.65	-0.29	0.00	-1.13	70.17	-1.30
22.62	4.51	-0.29	0.00	-1.12	71.18	-1.30
22.63	4.56	-0.29	0.01	-1.12	72.19	-1.29
22.65	4.67	-0.29	0.01	-1.11	73.18	-1.30
22.67	4.81	-0.29	0.02	-1.11	74.16	-1.30
22.68	4.90	-0.28	0.02	-1.11	75.14	-1.30
22.70	4.88	-0.28	0.03	-1.10	76.11	-1.30
22.72	4.88	-0.28	0.03	-1.10	77.06	-1.30
22.73	4.90	-0.28	0.04	-1.09	78.00	-1.30
22.75	4.78	-0.27	0.04	-1.08	78.94	-1.30
22.77	4.65	-0.27	0.05	-1.07	79.85	-1.29
22.78	4.70	-0.26	0.06	-1.07	80.75	-1.29
22.80	4.81	-0.26	0.07	-1.06	81.64	-1.29
22.82	4.88	-0.25	0.08	-1.05	82.51	-1.28
22.83	4.93	-0.24	0.09	-1.04	83.37	-1.27
22.85	4.98	-0.23	0.10	-1.03	84.21	-1.27
22.87	4.98	-0.21	0.12	-1.02	85.04	-1.28
22.88	4.99	-0.20	0.13	-1.01	85.84	-1.29
22.90	5.12	-0.19	0.14	-1.00	86.62	-1.29
22.92	5.13	-0.18	0.16	-0.98	87.39	-1.28
22.93	5.16	-0.16	0.17	-0.97	88.13	-1.27
22.95	5.29	-0.14	0.19	-0.95	88.84	-1.27
22.97	5.32	-0.12	0.21	-0.94	89.54	-1.27
22.98	5.36	-0.09	0.23	-0.91	90.21	-1.27
23.00	5.49	-0.07	0.25	-0.89	90.86	-1.27
23.02	5.61	-0.04	0.27	-0.86	91.48	-1.27
23.03	5.72	-0.01	0.30	-0.84	92.08	-1.26
23.05	5.87	0.03	0.32	-0.80	92.66	-1.26
23.07	5.95	0.07	0.35	-0.77	93.22	-1.26
23.08	5.98	0.12	0.38	-0.74	93.75	-1.26
23.10	6.20	0.17	0.42	-0.71	94.26	-1.26
23.12	6.41	0.22	0.45	-0.66	94.74	-1.25
23.13	6.65	0.28	0.49	-0.62	95.20	-1.25
23.15	6.77	0.35	0.52	-0.57	95.64	-1.25
23.17	6.85	0.42	0.56	-0.52	96.05	-1.25
23.18	6.96	0.49	0.61	-0.46	96.44	-1.24
23.20	7.11	0.58	0.65	-0.40	96.81	-1.24
23.22	7.33	0.67	0.70	-0.33	97.17	-1.24
23.23	7.56	0.76	0.75	-0.26	97.51	-1.24
23.25	7.90	0.86	0.80	-0.18	97.83	-1.24
23.27	8.16	0.97	0.86	-0.10	98.12	-1.24
23.28	8.44	1.09	0.91	0.00	98.40	-1.24
23.30	8.69	1.21	0.98	0.09	98.66	-1.24
23.32	8.92	1.35	1.04	0.20	98.90	-1.24
23.33	9.34	1.49	1.11	0.32	99.11	-1.24
23.35	9.83	1.64	1.19	0.44	99.31	-1.23
23.37	10.26	1.80	1.27	0.57	99.48	-1.22
23.38	10.71	1.97	1.35	0.72	99.64	-1.22
23.40	11.11	2.15	1.43	0.87	99.77	-1.22
23.42	11.44	2.34	1.53	1.03	99.87	-1.22
23.43	11.84	2.54	1.62	1.20	99.95	-1.22
23.45	12.36	2.74	1.72	1.38	99.99	-1.22
23.47	12.78	2.96	1.82	1.57	100.00	-1.21
23.48	13.29	3.19	1.93	1.77	99.97	-1.22
23.50	13.93	3.42	2.04	1.99	99.89	-1.22
23.52	14.56	3.68	2.16	2.22	99.78	-1.22
23.53	15.16	3.94	2.28	2.47	99.62	-1.21
23.55	15.67	4.22	2.41	2.72	99.43	-1.20

23.57	16.37	4.50	2.55	2.98	99.18	-1.20
23.58	17.17	4.79	2.69	3.26	98.89	-1.20
23.60	17.90	5.09	2.83	3.55	98.55	-1.20
23.62	18.50	5.41	2.99	3.84	98.16	-1.20
23.63	19.07	5.73	3.14	4.16	97.70	-1.20
23.65	19.83	6.07	3.31	4.49	97.18	-1.19
23.67	20.68	6.42	3.47	4.83	96.60	-1.19
23.68	21.50	6.77	3.65	5.18	95.98	-1.18
23.70	22.29	7.14	3.83	5.56	95.30	-1.18
23.72	23.12	7.52	4.02	5.94	94.57	-1.18
23.73	23.96	7.91	4.22	6.34	93.79	-1.19
23.75	24.76	8.31	4.42	6.75	92.95	-1.19
23.77	25.78	8.72	4.64	7.17	92.07	-1.18
23.78	26.82	9.13	4.86	7.60	91.14	-1.18
23.80	27.82	9.56	5.08	8.04	90.15	-1.18
23.82	28.75	9.99	5.31	8.50	89.12	-1.19
23.83	29.54	10.44	5.55	8.97	88.04	-1.19
23.85	30.54	10.90	5.80	9.45	86.94	-1.19
23.87	31.73	11.36	6.05	9.95	85.82	-1.18
23.88	32.91	11.83	6.31	10.46	84.66	-1.16
23.90	33.93	12.30	6.58	10.98	83.47	-1.16
23.92	34.95	12.78	6.86	11.50	82.27	-1.16
23.93	36.03	13.28	7.15	12.04	81.04	-1.16
23.95	37.22	13.78	7.45	12.59	79.81	-1.16
23.97	38.44	14.28	7.76	13.13	78.57	-1.16
23.98	39.49	14.79	8.07	13.70	77.33	-1.15
24.00	40.68	15.31	8.39	14.28	76.11	-1.15
24.02	42.02	15.83	8.72	14.87	74.89	-1.16
24.03	43.21	16.36	9.06	15.46	73.67	-1.16
24.05	44.31	16.91	9.41	16.07	72.46	-1.15
24.07	45.53	17.45	9.77	16.67	71.26	-1.15
24.08	46.79	18.00	10.13	17.28	70.07	-1.14
24.10	47.93	18.54	10.51	17.91	68.89	-1.14
24.12	49.07	19.11	10.90	18.53	67.71	-1.14
24.13	50.32	19.68	11.29	19.17	66.55	-1.15
24.15	51.62	20.25	11.70	19.82	65.42	-1.14
24.17	52.92	20.83	12.11	20.47	64.31	-1.13
24.18	54.14	21.41	12.53	21.14	63.23	-1.13
24.20	55.39	21.99	12.97	21.81	62.18	-1.13
24.22	56.65	22.58	13.42	22.48	61.15	-1.13
24.23	57.84	23.17	13.87	23.15	60.15	-1.13
24.25	59.07	23.77	14.34	23.82	59.16	-1.13
24.27	60.25	24.37	14.82	24.50	58.18	-1.12
24.28	61.36	24.97	15.31	25.19	57.23	-1.12
24.30	62.55	25.59	15.80	25.88	56.30	-1.13
24.32	63.76	26.21	16.32	26.58	55.39	-1.12
24.33	64.90	26.83	16.84	27.29	54.52	-1.11
24.35	66.09	27.45	17.37	28.00	53.66	-1.11
24.37	67.37	28.08	17.91	28.72	52.82	-1.12
24.38	68.62	28.71	18.47	29.44	52.00	-1.11
24.40	69.75	29.35	19.04	30.16	51.20	-1.11
24.42	70.90	29.99	19.62	30.88	50.40	-1.12
24.43	72.04	30.64	20.21	31.60	49.62	-1.11
24.45	73.14	31.29	20.81	32.33	48.85	-1.10
24.47	74.19	31.95	21.43	33.07	48.10	-1.10
24.48	75.29	32.61	22.05	33.82	47.37	-1.10
24.50	76.38	33.27	22.69	34.57	46.65	-1.10
24.52	77.43	33.93	23.35	35.34	45.95	-1.10
24.53	78.47	34.61	24.01	36.09	45.26	-1.10
24.55	79.44	35.28	24.69	36.85	44.58	-1.09
24.57	80.45	35.97	25.38	37.61	43.91	-1.09
24.58	81.48	36.65	26.08	38.37	43.24	-1.09
24.60	82.46	37.35	26.79	39.14	42.58	-1.09
24.62	83.38	38.05	27.52	39.90	41.94	-1.09
24.63	84.30	38.76	28.26	40.68	41.31	-1.09
24.65	85.19	39.47	29.01	41.46	40.70	-1.09
24.67	86.11	40.18	29.77	42.26	40.10	-1.08
24.68	86.96	40.89	30.55	43.06	39.52	-1.07
24.70	87.74	41.62	31.33	43.85	38.94	-1.07

24.72	88.58	42.35	32.13	44.65	38.38	-1.07
24.73	89.44	43.08	32.94	45.46	37.82	-1.08
24.75	90.17	43.82	33.76	46.25	37.27	-1.07
24.77	90.77	44.57	34.59	47.06	36.73	-1.07
24.78	91.50	45.33	35.43	47.86	36.20	-1.07
24.80	92.13	46.10	36.28	48.69	35.69	-1.07
24.82	92.70	46.87	37.14	49.52	35.19	-1.07
24.83	93.31	47.63	38.02	50.36	34.70	-1.07
24.85	93.86	48.41	38.90	51.20	34.22	-1.07
24.87	94.45	49.19	39.79	52.04	33.75	-1.07
24.88	95.02	49.99	40.69	52.87	33.29	-1.06
24.90	95.55	50.79	41.61	53.71	32.83	-1.06
24.92	96.06	51.59	42.52	54.56	32.39	-1.06
24.93	96.52	52.41	43.45	55.41	31.95	-1.06
24.95	96.91	53.24	44.38	56.28	31.52	-1.05
24.97	97.28	54.06	45.33	57.15	31.11	-1.05
24.98	97.70	54.90	46.27	58.03	30.71	-1.05
25.00	98.04	55.74	47.22	58.92	30.32	-1.05
25.02	98.25	56.59	48.19	59.80	29.93	-1.05
25.03	98.50	57.44	49.15	60.68	29.55	-1.05
25.05	98.79	58.30	50.12	61.56	29.18	-1.05
25.07	98.96	59.17	51.10	62.44	28.80	-1.04
25.08	99.09	60.05	52.08	63.34	28.44	-1.03
25.10	99.32	60.94	53.07	64.24	28.08	-1.03
25.12	99.57	61.84	54.05	65.16	27.74	-1.03
25.13	99.66	62.74	55.04	66.08	27.41	-1.04
25.15	99.85	63.64	56.04	67.01	27.09	-1.03
25.17	100.00	64.55	57.03	67.93	26.77	-1.03
25.18	99.95	65.46	58.03	68.84	26.46	-1.03
25.20	99.83	66.39	59.03	69.75	26.15	-1.02
25.22	99.75	67.31	60.02	70.66	25.85	-1.02
25.23	99.78	68.25	61.02	71.57	25.55	-1.02
25.25	99.75	69.19	62.02	72.47	25.25	-1.02
25.27	99.74	70.14	63.01	73.39	24.97	-1.01
25.28	99.69	71.09	64.02	74.31	24.69	-1.00
25.30	99.60	72.04	65.01	75.24	24.42	-1.00
25.32	99.44	72.98	66.00	76.17	24.15	-1.01
25.33	99.15	73.93	66.99	77.09	23.88	-1.01
25.35	98.96	74.88	67.98	78.01	23.63	-1.00
25.37	98.72	75.83	68.96	78.91	23.37	-1.00
25.38	98.41	76.77	69.94	79.81	23.12	-1.00
25.40	98.22	77.72	70.91	80.70	22.87	-0.99
25.42	98.04	78.67	71.88	81.58	22.62	-0.99
25.43	97.68	79.62	72.85	82.45	22.38	-0.99
25.45	97.22	80.57	73.80	83.32	22.15	-0.98
25.47	96.88	81.49	74.75	84.19	21.92	-0.98
25.48	96.48	82.41	75.70	85.06	21.69	-0.98
25.50	95.89	83.33	76.63	85.90	21.47	-0.97
25.52	95.32	84.23	77.56	86.72	21.26	-0.97
25.53	94.84	85.12	78.47	87.53	21.04	-0.97
25.55	94.51	86.01	79.38	88.33	20.83	-0.97
25.57	94.05	86.88	80.28	89.10	20.62	-0.96
25.58	93.57	87.74	81.16	89.87	20.41	-0.95
25.60	93.06	88.58	82.04	90.63	20.22	-0.95
25.62	92.41	89.40	82.90	91.36	20.02	-0.95
25.63	91.84	90.20	83.75	92.07	19.83	-0.95
25.65	91.31	90.98	84.58	92.76	19.64	-0.94
25.67	90.83	91.74	85.40	93.42	19.46	-0.94
25.68	90.20	92.47	86.20	94.05	19.27	-0.94
25.70	89.47	93.17	86.99	94.65	19.09	-0.93
25.72	88.76	93.85	87.77	95.23	18.91	-0.93
25.73	88.02	94.50	88.53	95.78	18.74	-0.92
25.75	87.26	95.13	89.27	96.31	18.57	-0.92
25.77	86.58	95.73	89.99	96.81	18.40	-0.92
25.78	85.97	96.29	90.69	97.28	18.23	-0.91
25.80	85.26	96.82	91.37	97.71	18.07	-0.91
25.82	84.40	97.30	92.03	98.11	17.92	-0.90
25.83	83.68	97.74	92.67	98.48	17.76	-0.90
25.85	82.97	98.15	93.29	98.81	17.60	-0.89

25.87	82.12	98.52	93.88	99.09	17.44	-0.88
25.88	81.31	98.86	94.45	99.33	17.29	-0.88
25.90	80.56	99.17	95.00	99.54	17.14	-0.88
25.92	79.68	99.44	95.52	99.72	17.00	-0.88
25.93	78.84	99.65	96.02	99.85	16.85	-0.87
25.95	77.96	99.81	96.49	99.95	16.72	-0.87
25.97	77.08	99.93	96.93	100.00	16.58	-0.86
25.98	76.15	99.99	97.34	100.00	16.44	-0.85
26.00	75.21	100.00	97.73	99.95	16.30	-0.84
26.02	74.44	99.96	98.10	99.85	16.17	-0.83
26.03	73.57	99.87	98.43	99.68	16.03	-0.83
26.05	72.44	99.74	98.73	99.48	15.90	-0.82
26.07	71.39	99.56	99.00	99.23	15.77	-0.81
26.08	70.42	99.32	99.24	98.94	15.64	-0.80
26.10	69.37	99.02	99.45	98.59	15.52	-0.80
26.12	68.41	98.66	99.62	98.18	15.40	-0.80
26.13	67.40	98.24	99.76	97.73	15.27	-0.79
26.15	66.26	97.77	99.87	97.22	15.15	-0.79
26.17	65.13	97.24	99.94	96.66	15.03	-0.78
26.18	64.06	96.64	99.99	96.04	14.91	-0.77
26.20	62.97	95.99	100.00	95.37	14.80	-0.76
26.22	61.89	95.28	99.98	94.66	14.68	-0.74
26.23	60.71	94.52	99.93	93.91	14.57	-0.73
26.25	59.43	93.70	99.84	93.11	14.46	-0.72
26.27	58.25	92.82	99.71	92.27	14.35	-0.72
26.28	57.13	91.88	99.55	91.39	14.24	-0.71
26.30	55.95	90.89	99.36	90.46	14.13	-0.71
26.32	54.82	89.83	99.12	89.50	14.03	-0.70
26.33	53.83	88.73	98.86	88.51	13.92	-0.69
26.35	52.70	87.59	98.57	87.48	13.81	-0.68
26.37	51.50	86.41	98.23	86.43	13.71	-0.67
26.38	50.32	85.19	97.87	85.36	13.61	-0.66
26.40	49.07	83.94	97.47	84.26	13.51	-0.65
26.42	47.85	82.66	97.04	83.14	13.41	-0.64
26.43	46.68	81.33	96.57	82.01	13.32	-0.62
26.45	45.58	79.98	96.08	80.87	13.22	-0.61
26.47	44.45	78.60	95.55	79.73	13.12	-0.61
26.48	43.21	77.20	94.99	78.57	13.03	-0.59
26.50	42.09	75.78	94.41	77.41	12.93	-0.59
26.52	41.10	74.36	93.80	76.23	12.84	-0.57
26.53	40.19	72.93	93.16	75.06	12.75	-0.56
26.55	39.18	71.50	92.50	73.89	12.66	-0.55
26.57	38.15	70.07	91.81	72.74	12.57	-0.54
26.58	37.25	68.64	91.09	71.60	12.49	-0.53
26.60	36.34	67.21	90.35	70.47	12.40	-0.52
26.62	35.41	65.78	89.59	69.36	12.32	-0.51
26.63	34.48	64.37	88.82	68.25	12.23	-0.50
26.65	33.68	62.98	88.03	67.15	12.15	-0.48
26.67	32.95	61.60	87.23	66.06	12.06	-0.46
26.68	32.16	60.25	86.42	64.99	11.98	-0.45
26.70	31.38	58.93	85.59	63.93	11.90	-0.44
26.72	30.76	57.63	84.75	62.90	11.82	-0.43
26.73	30.19	56.35	83.90	61.88	11.74	-0.42
26.75	29.54	55.10	83.05	60.89	11.66	-0.40
26.77	28.92	53.86	82.19	59.92	11.59	-0.38
26.78	28.32	52.66	81.33	58.96	11.51	-0.37
26.80	27.65	51.48	80.47	58.02	11.44	-0.36
26.82	27.06	50.33	79.62	57.10	11.36	-0.34
26.83	26.65	49.21	78.76	56.18	11.29	-0.33
26.85	26.09	48.13	77.92	55.30	11.21	-0.31
26.87	25.41	47.08	77.07	54.43	11.14	-0.30
26.88	24.87	46.06	76.23	53.57	11.07	-0.29
26.90	24.54	45.07	75.39	52.75	11.00	-0.27
26.92	24.23	44.10	74.56	51.94	10.93	-0.26
26.93	23.83	43.16	73.74	51.15	10.86	-0.24
26.95	23.37	42.25	72.93	50.38	10.79	-0.22
26.97	22.86	41.36	72.13	49.61	10.73	-0.21
26.98	22.41	40.49	71.34	48.86	10.66	-0.19
27.00	22.04	39.65	70.56	48.13	10.60	-0.18

27.02	21.70	38.84	69.78	47.42	10.54	-0.16
27.03	21.42	38.05	69.02	46.71	10.48	-0.14
27.05	21.21	37.29	68.26	46.03	10.42	-0.13
27.07	20.91	36.56	67.52	45.37	10.35	-0.12
27.08	20.48	35.84	66.78	44.72	10.29	-0.11
27.10	20.15	35.14	66.05	44.08	10.23	-0.08
27.12	19.94	34.45	65.34	43.46	10.17	-0.06
27.13	19.78	33.78	64.64	42.84	10.10	-0.05
27.15	19.61	33.14	63.94	42.24	10.04	-0.03
27.17	19.40	32.51	63.25	41.64	9.98	-0.01
27.18	19.07	31.90	62.57	41.05	9.92	0.00
27.20	18.79	31.31	61.90	40.49	9.86	0.01
27.22	18.64	30.74	61.24	39.94	9.81	0.02
27.23	18.45	30.18	60.58	39.40	9.75	0.04
27.25	18.27	29.63	59.93	38.87	9.70	0.06
27.27	18.01	29.10	59.29	38.35	9.64	0.07
27.28	17.67	28.58	58.66	37.83	9.59	0.09
27.30	17.45	28.08	58.04	37.32	9.53	0.11
27.32	17.20	27.59	57.42	36.82	9.48	0.13
27.33	16.99	27.11	56.81	36.34	9.43	0.14
27.35	16.89	26.65	56.21	35.86	9.38	0.16
27.37	16.72	26.19	55.61	35.38	9.33	0.17
27.38	16.55	25.75	55.02	34.93	9.28	0.19
27.40	16.43	25.32	54.43	34.48	9.23	0.21
27.42	16.29	24.89	53.85	34.04	9.18	0.22
27.43	16.11	24.48	53.27	33.60	9.13	0.23
27.45	15.94	24.08	52.70	33.16	9.08	0.25
27.47	15.80	23.68	52.14	32.73	9.03	0.26
27.48	15.75	23.29	51.58	32.29	8.98	0.28
27.50	15.81	22.92	51.03	31.88	8.94	0.30
27.52	15.77	22.56	50.48	31.48	8.89	0.32
27.53	15.52	22.20	49.94	31.09	8.85	0.33
27.55	15.27	21.85	49.40	30.71	8.80	0.34
27.57	15.15	21.51	48.87	30.34	8.76	0.36
27.58	15.05	21.18	48.34	29.96	8.71	0.38
27.60	14.93	20.85	47.82	29.60	8.67	0.39
27.62	14.87	20.53	47.30	29.23	8.63	0.41
27.63	14.74	20.22	46.79	28.87	8.58	0.42
27.65	14.59	19.91	46.28	28.51	8.54	0.44
27.67	14.48	19.62	45.78	28.16	8.50	0.44
27.68	14.44	19.34	45.28	27.82	8.46	0.45
27.70	14.39	19.06	44.78	27.48	8.42	0.47
27.72	14.28	18.78	44.29	27.15	8.38	0.48
27.73	14.17	18.50	43.80	26.83	8.33	0.50
27.75	14.14	18.24	43.32	26.52	8.30	0.51
27.77	14.05	17.98	42.84	26.20	8.26	0.52
27.78	13.91	17.73	42.37	25.88	8.21	0.53
27.80	13.80	17.48	41.90	25.56	8.17	0.55
27.82	14.17	17.23	41.43	25.25	8.14	0.56
27.83	18.62	17.00	40.97	24.95	8.10	0.57
27.85	24.27	16.76	40.51	24.65	8.06	0.58
27.87	21.17	16.54	40.05	24.36	8.02	0.59
27.88	16.20	16.32	39.61	24.07	7.98	0.61
27.90	14.88	16.11	39.16	23.79	7.95	0.62
27.92	14.54	15.90	38.72	23.52	7.91	0.62
27.93	14.45	15.68	38.29	23.24	7.87	0.64
27.95	14.45	15.47	37.86	22.96	7.83	0.66
27.97	14.28	15.27	37.43	22.69	7.80	0.66
27.98	14.11	15.08	37.01	22.41	7.76	0.66
28.00	14.00	14.89	36.59	22.15	7.73	0.67
28.02	13.96	14.71	36.17	21.89	7.69	0.68
28.03	13.96	14.53	35.76	21.65	7.66	0.70
28.05	13.96	14.35	35.35	21.40	7.63	0.70
28.07	13.97	14.17	34.95	21.15	7.59	0.71
28.08	13.91	13.99	34.56	20.90	7.55	0.72
28.10	13.80	13.82	34.16	20.65	7.52	0.73
28.12	13.66	13.66	33.77	20.41	7.49	0.73
28.13	13.48	13.50	33.39	20.17	7.46	0.74
28.15	13.40	13.34	33.00	19.94	7.42	0.74

28.17	13.31	13.18	32.62	19.71	7.39	0.75
28.18	13.18	13.03	32.25	19.48	7.36	0.75
28.20	13.18	12.88	31.88	19.27	7.33	0.76
28.22	13.21	12.74	31.51	19.05	7.30	0.76
28.23	13.23	12.59	31.15	18.84	7.26	0.77
28.25	13.15	12.45	30.79	18.63	7.23	0.77
28.27	13.08	12.31	30.44	18.41	7.20	0.77
28.28	13.01	12.17	30.09	18.20	7.17	0.77
28.30	12.91	12.04	29.74	17.99	7.14	0.77
28.32	12.87	11.92	29.39	17.79	7.11	0.78
28.33	12.97	11.79	29.05	17.59	7.08	0.78
28.35	12.95	11.67	28.72	17.40	7.06	0.78
28.37	12.86	11.54	28.39	17.21	7.03	0.78
28.38	12.87	11.42	28.06	17.02	7.00	0.78
28.40	12.86	11.30	27.73	16.83	6.97	0.79
28.42	12.69	11.19	27.42	16.64	6.94	0.80
28.43	12.61	11.07	27.10	16.46	6.91	0.80
28.45	12.86	10.96	26.79	16.27	6.88	0.79
28.47	12.97	10.85	26.48	16.09	6.86	0.79
28.48	12.74	10.74	26.17	15.92	6.83	0.79
28.50	12.60	10.64	25.87	15.75	6.81	0.79
28.52	12.61	10.54	25.57	15.58	6.78	0.80
28.53	12.57	10.44	25.28	15.41	6.75	0.80
28.55	12.44	10.33	24.99	15.24	6.73	0.80
28.57	12.49	10.23	24.70	15.08	6.70	0.79
28.58	12.53	10.13	24.42	14.91	6.68	0.79
28.60	12.52	10.04	24.14	14.75	6.65	0.78
28.62	12.43	9.95	23.86	14.59	6.62	0.78
28.63	12.38	9.85	23.59	14.44	6.59	0.78
28.65	12.44	9.76	23.32	14.28	6.57	0.78
28.67	12.47	9.68	23.05	14.13	6.54	0.78
28.68	12.24	9.59	22.79	13.98	6.52	0.78
28.70	11.75	9.51	22.53	13.84	6.50	0.77
28.72	11.55	9.42	22.27	13.69	6.47	0.77
28.73	11.53	9.33	22.02	13.54	6.44	0.77
28.75	11.42	9.25	21.77	13.40	6.42	0.76
28.77	11.36	9.17	21.52	13.25	6.40	0.75
28.78	11.28	9.09	21.28	13.11	6.37	0.74
28.80	11.14	9.01	21.03	12.98	6.35	0.74
28.82	11.16	8.94	20.79	12.85	6.33	0.74
28.83	11.25	8.86	20.56	12.72	6.30	0.73
28.85	11.25	8.79	20.32	12.59	6.28	0.73
28.87	11.13	8.72	20.09	12.46	6.25	0.73
28.88	11.08	8.65	19.87	12.33	6.23	0.72
28.90	11.16	8.57	19.65	12.20	6.21	0.73
28.92	11.13	8.51	19.42	12.08	6.18	0.72
28.93	11.00	8.44	19.21	11.95	6.16	0.71
28.95	10.94	8.37	18.99	11.83	6.14	0.70
28.97	10.96	8.31	18.78	11.71	6.11	0.70
28.98	10.96	8.24	18.57	11.59	6.09	0.70
29.00	10.83	8.18	18.36	11.48	6.07	0.69
29.02	10.68	8.11	18.15	11.37	6.05	0.68
29.03	10.68	8.05	17.95	11.26	6.03	0.68
29.05	10.79	7.98	17.75	11.15	6.01	0.67
29.07	10.88	7.92	17.56	11.03	5.99	0.66
29.08	10.85	7.86	17.36	10.92	5.97	0.66
29.10	10.80	7.80	17.17	10.82	5.95	0.65
29.12	10.74	7.74	16.98	10.71	5.93	0.64
29.13	10.65	7.68	16.79	10.60	5.91	0.64
29.15	10.59	7.62	16.61	10.50	5.89	0.63
29.17	10.62	7.57	16.43	10.40	5.87	0.63
29.18	10.68	7.52	16.25	10.30	5.85	0.63
29.20	10.63	7.46	16.07	10.20	5.83	0.63
29.22	10.57	7.41	15.89	10.10	5.81	0.62
29.23	10.53	7.35	15.72	10.00	5.80	0.61
29.25	10.51	7.30	15.55	9.90	5.78	0.61
29.27	10.60	7.25	15.38	9.80	5.76	0.60
29.28	10.68	7.19	15.22	9.71	5.75	0.60
29.30	10.60	7.14	15.05	9.62	5.73	0.59

29.32	10.49	7.09	14.89	9.53	5.71	0.58
29.33	10.46	7.04	14.73	9.45	5.69	0.58
29.35	10.37	6.99	14.57	9.36	5.68	0.57
29.37	10.28	6.95	14.42	9.27	5.66	0.56
29.38	10.28	6.90	14.27	9.18	5.64	0.56
29.40	10.34	6.85	14.11	9.10	5.62	0.55
29.42	10.32	6.81	13.96	9.01	5.60	0.55
29.43	10.17	6.75	13.81	8.92	5.58	0.55
29.45	10.09	6.71	13.66	8.85	5.57	0.54
29.47	10.17	6.66	13.52	8.76	5.55	0.54
29.48	10.26	6.62	13.38	8.68	5.53	0.53
29.50	10.26	6.58	13.23	8.60	5.51	0.53
29.52	10.22	6.53	13.09	8.52	5.49	0.53
29.53	10.23	6.48	12.96	8.45	5.47	0.52
29.55	10.20	6.44	12.83	8.37	5.46	0.52
29.57	10.17	6.40	12.69	8.29	5.44	0.51
29.58	10.28	6.36	12.56	8.22	5.42	0.51
29.60	10.28	6.32	12.43	8.15	5.41	0.50
29.62	10.11	6.28	12.30	8.08	5.39	0.49
29.63	9.95	6.24	12.17	8.01	5.37	0.50
29.65	9.94	6.20	12.04	7.94	5.35	0.50
29.67	10.02	6.16	11.92	7.87	5.34	0.49
29.68	10.00	6.12	11.79	7.80	5.33	0.49
29.70	9.94	6.08	11.68	7.73	5.31	0.48
29.72	9.94	6.04	11.56	7.66	5.29	0.48
29.73	9.94	6.00	11.44	7.60	5.28	0.48
29.75	9.94	5.96	11.32	7.53	5.26	0.48
29.77	10.02	5.93	11.20	7.46	5.24	0.48
29.78	10.03	5.89	11.09	7.40	5.23	0.48
29.80	10.03	5.85	10.97	7.34	5.22	0.48
29.82	9.98	5.82	10.86	7.28	5.20	0.48
29.83	9.88	5.78	10.75	7.21	5.18	0.48
29.85	9.88	5.75	10.65	7.15	5.17	0.47
29.87	9.95	5.71	10.54	7.10	5.15	0.47
29.88	9.95	5.67	10.43	7.03	5.14	0.47
29.90	9.77	5.64	10.33	6.97	5.13	0.46
29.92	9.80	5.60	10.22	6.91	5.11	0.46
29.93	9.92	5.57	10.12	6.86	5.10	0.46
29.95	9.89	5.53	10.02	6.80	5.08	0.46
29.97	9.85	5.50	9.92	6.75	5.07	0.46
29.98	9.85	5.47	9.82	6.69	5.06	0.46
30.00	9.77	5.43	9.72	6.63	5.04	0.46
30.02	9.63	5.40	9.62	6.58	5.03	0.45
30.03	9.60	5.37	9.53	6.52	5.02	0.46
30.05	9.64	5.34	9.44	6.47	5.00	0.46
30.07	9.72	5.31	9.34	6.42	4.99	0.46
30.08	9.72	5.28	9.25	6.37	4.98	0.45
30.10	9.57	5.24	9.16	6.32	4.96	0.45
30.12	9.49	5.21	9.07	6.27	4.94	0.44
30.13	9.55	5.18	8.98	6.22	4.93	0.45
30.15	9.60	5.15	8.89	6.18	4.92	0.45
30.17	9.51	5.12	8.81	6.13	4.91	0.45
30.18	9.46	5.09	8.72	6.08	4.90	0.45
30.20	9.54	5.06	8.64	6.03	4.88	0.45
30.22	9.58	5.03	8.55	5.98	4.87	0.45
30.23	9.52	5.01	8.47	5.93	4.86	0.45
30.25	9.47	4.98	8.38	5.89	4.84	0.46
30.27	9.41	4.95	8.30	5.85	4.83	0.46
30.28	9.40	4.92	8.22	5.80	4.82	0.46
30.30	9.44	4.89	8.14	5.75	4.81	0.46
30.32	9.46	4.86	8.06	5.71	4.80	0.46
30.33	9.49	4.83	7.99	5.67	4.79	0.46
30.35	9.60	4.80	7.91	5.63	4.78	0.46
30.37	9.60	4.78	7.84	5.58	4.76	0.46
30.38	9.47	4.75	7.76	5.54	4.75	0.47
30.40	9.47	4.73	7.69	5.50	4.74	0.47
30.42	9.41	4.70	7.61	5.46	4.73	0.47
30.43	9.30	4.67	7.54	5.42	4.72	0.47
30.45	9.27	4.64	7.47	5.38	4.71	0.47

30.47	9.29	4.62	7.40	5.34	4.69	0.48
30.48	9.27	4.59	7.33	5.30	4.68	0.48
30.50	9.32	4.57	7.26	5.26	4.67	0.48
30.52	9.32	4.55	7.19	5.22	4.66	0.48
30.53	9.21	4.52	7.13	5.18	4.65	0.47
30.55	9.17	4.50	7.06	5.15	4.64	0.47
30.57	9.23	4.47	6.99	5.11	4.63	0.47
30.58	9.32	4.44	6.92	5.08	4.62	0.47
30.60	9.24	4.43	6.86	5.04	4.61	0.48
30.62	9.23	4.41	6.80	5.01	4.60	0.48
30.63	9.37	4.38	6.74	4.97	4.59	0.49
30.65	9.40	4.36	6.67	4.93	4.58	0.48
30.67	9.23	4.34	6.61	4.90	4.57	0.48
30.68	9.03	4.31	6.55	4.87	4.56	0.49
30.70	8.95	4.28	6.49	4.83	4.55	0.49
30.72	8.95	4.26	6.43	4.80	4.54	0.48
30.73	9.03	4.24	6.37	4.76	4.53	0.49
30.75	9.18	4.22	6.31	4.73	4.52	0.50
30.77	9.18	4.20	6.25	4.70	4.51	0.49
30.78	9.03	4.18	6.20	4.67	4.50	0.49
30.80	8.98	4.16	6.14	4.64	4.49	0.49
30.82	9.09	4.14	6.08	4.61	4.48	0.50
30.83	9.07	4.11	6.03	4.58	4.47	0.51
30.85	9.03	4.09	5.98	4.54	4.46	0.51
30.87	9.10	4.07	5.92	4.51	4.45	0.51
30.88	9.07	4.05	5.87	4.48	4.44	0.51
30.90	9.01	4.03	5.82	4.45	4.43	0.52
30.92	9.04	4.02	5.77	4.42	4.43	0.52
30.93	9.12	4.00	5.71	4.40	4.42	0.52
30.95	9.15	3.97	5.66	4.37	4.41	0.52
30.97	9.00	3.95	5.61	4.34	4.40	0.52
30.98	8.78	3.93	5.56	4.31	4.39	0.52
31.00	8.78	3.91	5.51	4.28	4.39	0.53
31.02	8.86	3.90	5.46	4.25	4.38	0.53
31.03	8.89	3.88	5.41	4.22	4.37	0.53
31.05	8.95	3.86	5.37	4.20	4.36	0.53
31.07	8.96	3.84	5.32	4.18	4.35	0.53
31.08	8.95	3.82	5.28	4.14	4.34	0.54
31.10	8.98	3.80	5.23	4.12	4.33	0.55
31.12	9.00	3.79	5.18	4.09	4.32	0.54
31.13	8.96	3.77	5.14	4.06	4.32	0.54
31.15	8.92	3.75	5.10	4.04	4.31	0.55
31.17	8.79	3.73	5.05	4.02	4.30	0.56
31.18	8.64	3.71	5.01	3.99	4.30	0.56
31.20	8.70	3.70	4.97	3.97	4.29	0.56
31.22	8.81	3.68	4.92	3.94	4.28	0.56
31.23	8.79	3.66	4.88	3.92	4.28	0.56
31.25	8.79	3.64	4.84	3.90	4.27	0.56
31.27	8.92	3.63	4.80	3.87	4.26	0.56
31.28	9.01	3.61	4.76	3.85	4.25	0.56
31.30	9.00	3.60	4.72	3.83	4.24	0.57
31.32	8.92	3.58	4.68	3.80	4.24	0.58
31.33	8.84	3.56	4.64	3.78	4.23	0.58
31.35	8.75	3.55	4.60	3.76	4.22	0.58
31.37	8.64	3.54	4.56	3.74	4.22	0.58
31.38	8.59	3.52	4.52	3.71	4.21	0.58
31.40	8.59	3.50	4.49	3.68	4.20	0.59
31.42	8.61	3.48	4.45	3.66	4.20	0.59
31.43	8.70	3.47	4.42	3.64	4.19	0.59
31.45	8.79	3.45	4.38	3.62	4.19	0.59
31.47	8.81	3.44	4.34	3.60	4.18	0.59
31.48	8.66	3.43	4.31	3.58	4.17	0.60
31.50	8.61	3.41	4.28	3.56	4.17	0.61
31.52	8.64	3.40	4.24	3.54	4.16	0.61
31.53	8.61	3.39	4.20	3.52	4.15	0.61
31.55	8.53	3.37	4.17	3.50	4.15	0.61
31.57	8.53	3.35	4.14	3.48	4.14	0.62
31.58	8.64	3.34	4.11	3.46	4.13	0.62
31.60	8.62	3.32	4.07	3.44	4.12	0.62

31.62	8.61	3.31	4.04	3.42	4.12	0.62
31.63	8.59	3.29	4.01	3.40	4.11	0.63
31.65	8.55	3.28	3.98	3.38	4.11	0.64
31.67	8.59	3.27	3.95	3.36	4.10	0.63
31.68	8.62	3.26	3.92	3.34	4.09	0.63
31.70	8.49	3.24	3.89	3.32	4.09	0.63
31.72	8.42	3.22	3.85	3.30	4.09	0.64
31.73	8.58	3.21	3.82	3.29	4.08	0.65
31.75	8.66	3.20	3.79	3.27	4.08	0.65
31.77	8.61	3.18	3.77	3.25	4.08	0.65
31.78	8.53	3.17	3.74	3.23	4.07	0.66
31.80	8.53	3.16	3.71	3.22	4.07	0.66
31.82	8.52	3.15	3.68	3.20	4.06	0.66
31.83	8.52	3.13	3.65	3.18	4.06	0.67
31.85	8.55	3.12	3.63	3.16	4.05	0.67
31.87	8.59	3.11	3.60	3.14	4.05	0.67
31.88	8.62	3.09	3.58	3.13	4.05	0.67
31.90	8.58	3.08	3.55	3.11	4.04	0.67
31.92	8.55	3.07	3.52	3.09	4.04	0.67
31.93	8.55	3.06	3.50	3.08	4.04	0.68
31.95	8.49	3.05	3.47	3.06	4.04	0.68
31.97	8.42	3.04	3.44	3.04	4.03	0.69
31.98	8.39	3.02	3.42	3.03	4.03	0.69
32.00	8.44	3.01	3.40	3.02	4.02	0.69
32.02	8.55	3.00	3.37	3.00	4.02	0.69
32.03	8.59	2.99	3.35	2.98	4.02	0.70
32.05	8.66	2.98	3.32	2.97	4.01	0.70
32.07	8.58	2.96	3.30	2.95	4.01	0.70
32.08	8.44	2.95	3.28	2.93	4.00	0.71
32.10	8.41	2.94	3.25	2.92	4.00	0.71
32.12	8.45	2.93	3.23	2.91	4.00	0.71
32.13	8.53	2.92	3.21	2.89	4.00	0.71
32.15	8.52	2.91	3.19	2.87	4.00	0.71
32.17	8.44	2.90	3.16	2.86	4.00	0.72
32.18	8.39	2.89	3.14	2.85	3.99	0.72
32.20	8.33	2.88	3.12	2.83	3.99	0.72
32.22	8.35	2.86	3.10	2.82	3.98	0.73
32.23	8.38	2.86	3.08	2.80	3.98	0.73
32.25	8.36	2.85	3.06	2.79	3.98	0.73
32.27	8.49	2.84	3.03	2.77	3.98	0.73
32.28	8.50	2.82	3.01	2.76	3.97	0.73
32.30	8.33	2.81	2.99	2.74	3.97	0.74
32.32	8.22	2.80	2.97	2.73	3.97	0.75
32.33	8.25	2.79	2.95	2.72	3.97	0.75
32.35	8.27	2.78	2.93	2.71	3.96	0.75
32.37	8.18	2.77	2.91	2.69	3.96	0.76
32.38	8.22	2.76	2.90	2.68	3.96	0.76
32.40	8.42	2.76	2.88	2.66	3.96	0.77
32.42	8.49	2.75	2.86	2.65	3.95	0.77
32.43	8.47	2.74	2.84	2.64	3.95	0.77
32.45	8.42	2.73	2.82	2.63	3.95	0.77
32.47	8.39	2.71	2.81	2.61	3.95	0.77
32.48	8.36	2.70	2.79	2.60	3.95	0.78
32.50	8.28	2.70	2.77	2.58	3.94	0.78
32.52	8.22	2.69	2.75	2.57	3.94	0.78
32.53	8.28	2.68	2.73	2.56	3.94	0.78
32.55	8.33	2.67	2.72	2.55	3.94	0.79
32.57	8.30	2.66	2.70	2.53	3.93	0.78
32.58	8.27	2.65	2.68	2.52	3.93	0.79
32.60	8.16	2.64	2.66	2.51	3.93	0.79
32.62	8.10	2.63	2.65	2.50	3.93	0.80
32.63	8.10	2.62	2.63	2.48	3.93	0.81
32.65	8.08	2.61	2.61	2.47	3.93	0.81
32.67	8.19	2.60	2.60	2.46	3.92	0.81
32.68	8.18	2.59	2.58	2.45	3.92	0.81
32.70	8.04	2.59	2.57	2.44	3.92	0.81
32.72	8.01	2.58	2.55	2.43	3.92	0.82
32.73	7.99	2.57	2.54	2.41	3.92	0.82
32.75	8.01	2.56	2.52	2.40	3.92	0.82

32.77	8.05	2.55	2.50	2.39	3.92	0.83
32.78	8.11	2.54	2.49	2.38	3.92	0.84
32.80	8.15	2.54	2.47	2.37	3.92	0.84
32.82	8.10	2.53	2.46	2.36	3.92	0.85
32.83	8.10	2.52	2.45	2.34	3.92	0.86
32.85	8.04	2.51	2.43	2.33	3.92	0.86
32.87	7.98	2.50	2.42	2.32	3.92	0.86
32.88	8.05	2.50	2.40	2.31	3.91	0.86
32.90	8.16	2.49	2.39	2.30	3.91	0.86
32.92	8.08	2.48	2.37	2.29	3.91	0.87
32.93	8.01	2.47	2.36	2.28	3.91	0.88
32.95	8.02	2.46	2.35	2.27	3.91	0.88
32.97	8.01	2.45	2.33	2.25	3.91	0.89
32.98	8.01	2.45	2.32	2.24	3.91	0.89
33.00	8.05	2.44	2.31	2.23	3.91	0.89
33.02	8.07	2.43	2.29	2.22	3.92	0.89
33.03	7.94	2.42	2.28	2.21	3.91	0.90
33.05	7.84	2.41	2.27	2.20	3.91	0.90
33.07	7.91	2.41	2.25	2.19	3.91	0.91
33.08	8.10	2.40	2.24	2.18	3.91	0.91
33.10	8.13	2.39	2.23	2.17	3.91	0.91
33.12	8.11	2.38	2.22	2.16	3.92	0.92
33.13	8.07	2.37	2.20	2.15	3.92	0.92
33.15	7.93	2.37	2.19	2.14	3.92	0.93
33.17	8.01	2.36	2.18	2.13	3.92	0.93
33.18	8.18	2.36	2.17	2.12	3.92	0.94
33.20	8.19	2.35	2.16	2.11	3.92	0.95
33.22	8.08	2.34	2.14	2.10	3.92	0.96
33.23	7.96	2.33	2.13	2.09	3.92	0.96
33.25	8.01	2.33	2.12	2.08	3.92	0.96
33.27	8.08	2.32	2.11	2.07	3.93	0.96
33.28	8.08	2.31	2.10	2.06	3.93	0.97
33.30	8.11	2.30	2.09	2.05	3.93	0.98
33.32	8.13	2.30	2.07	2.04	3.93	0.99
33.33	8.08	2.29	2.06	2.03	3.93	1.00
33.35	8.08	2.29	2.05	2.02	3.93	1.00
33.37	8.05	2.28	2.04	2.01	3.94	1.01
33.38	7.94	2.27	2.03	2.00	3.94	1.01
33.40	7.94	2.27	2.02	1.99	3.94	1.02
33.42	8.01	2.26	2.01	1.98	3.94	1.03
33.43	7.94	2.25	2.00	1.97	3.94	1.04
33.45	7.91	2.25	1.99	1.96	3.94	1.04
33.47	7.88	2.24	1.98	1.96	3.94	1.05
33.48	7.90	2.23	1.97	1.95	3.95	1.06
33.50	7.98	2.23	1.96	1.94	3.95	1.06
33.52	7.93	2.22	1.95	1.93	3.95	1.06
33.53	7.88	2.21	1.94	1.92	3.95	1.08
33.55	8.10	2.21	1.93	1.91	3.95	1.08
33.57	8.28	2.20	1.92	1.90	3.95	1.09
33.58	8.27	2.20	1.91	1.89	3.96	1.10
33.60	8.11	2.19	1.90	1.89	3.96	1.11
33.62	7.93	2.18	1.89	1.88	3.96	1.13
33.63	7.87	2.18	1.88	1.87	3.96	1.14
33.65	7.85	2.17	1.87	1.86	3.96	1.15
33.67	7.79	2.16	1.86	1.85	3.97	1.17
33.68	7.81	2.16	1.85	1.85	3.97	1.18
33.70	7.87	2.15	1.84	1.84	3.97	1.20
33.72	7.93	2.14	1.84	1.83	3.97	1.21
33.73	7.99	2.14	1.83	1.82	3.98	1.22
33.75	8.10	2.14	1.82	1.81	3.98	1.23
33.77	8.05	2.13	1.81	1.80	3.98	1.23
33.78	8.07	2.12	1.80	1.79	3.98	1.25
33.80	8.15	2.11	1.79	1.79	3.98	1.27
33.82	8.13	2.11	1.78	1.78	3.99	1.28
33.83	8.02	2.10	1.77	1.77	3.99	1.30
33.85	7.96	2.10	1.76	1.76	3.99	1.31
33.87	7.98	2.10	1.76	1.75	3.99	1.33
33.88	8.02	2.09	1.75	1.74	3.99	1.35
33.90	8.18	2.08	1.74	1.74	3.99	1.37

33.92	8.13	2.08	1.73	1.73	4.00	1.39
33.93	7.98	2.08	1.72	1.72	4.00	1.42
33.95	7.98	2.07	1.72	1.72	4.00	1.45
33.97	7.91	2.06	1.71	1.71	4.00	1.47
33.98	7.93	2.06	1.70	1.70	4.00	1.49
34.00	8.07	2.05	1.69	1.69	4.01	1.51
34.02	8.02	2.05	1.69	1.68	4.01	1.54
34.03	7.96	2.04	1.68	1.67	4.01	1.57
34.05	8.05	2.04	1.67	1.67	4.02	1.60
34.07	8.13	2.03	1.66	1.66	4.02	1.62
34.08	8.02	2.03	1.65	1.66	4.02	1.65
34.10	7.94	2.02	1.64	1.65	4.02	1.69
34.12	7.98	2.02	1.63	1.64	4.02	1.73
34.13	7.94	2.01	1.63	1.64	4.02	1.76
34.15	7.88	2.00	1.62	1.63	4.03	1.80
34.17	7.88	2.00	1.62	1.62	4.03	1.84
34.18	7.94	2.00	1.61	1.61	4.03	1.88
34.20	7.94	2.00	1.60	1.61	4.03	1.92
34.22	7.84	1.99	1.59	1.60	4.03	1.97
34.23	7.81	1.99	1.58	1.59	4.04	2.01
34.25	7.87	1.99	1.58	1.58	4.04	2.06
34.27	7.93	1.98	1.57	1.58	4.04	2.11
34.28	7.90	1.97	1.56	1.58	4.05	2.16
34.30	7.77	1.97	1.56	1.58	4.05	2.22
34.32	7.74	1.96	1.55	1.57	4.05	2.28
34.33	7.87	1.96	1.54	1.56	4.05	2.35
34.35	7.99	1.96	1.54	1.55	4.05	2.41
34.37	7.88	1.96	1.53	1.54	4.05	2.47
34.38	7.82	1.95	1.52	1.54	4.06	2.54
34.40	7.94	1.95	1.52	1.53	4.06	2.62
34.42	7.98	1.95	1.51	1.52	4.06	2.69
34.43	7.90	1.94	1.50	1.52	4.06	2.76
34.45	7.88	1.94	1.50	1.51	4.06	2.85
34.47	7.91	1.93	1.49	1.51	4.07	2.93
34.48	7.90	1.93	1.49	1.50	4.07	3.01
34.50	7.87	1.92	1.48	1.49	4.07	3.11
34.52	7.91	1.92	1.47	1.48	4.07	3.20
34.53	7.84	1.92	1.46	1.48	4.07	3.30
34.55	7.74	1.92	1.46	1.48	4.07	3.40
34.57	7.79	1.91	1.45	1.47	4.07	3.51
34.58	7.82	1.91	1.45	1.46	4.08	3.62
34.60	7.88	1.91	1.44	1.46	4.08	3.73
34.62	8.01	1.90	1.43	1.45	4.08	3.85
34.63	8.01	1.89	1.43	1.44	4.08	3.97
34.65	7.98	1.89	1.42	1.44	4.08	4.09
34.67	7.93	1.89	1.42	1.43	4.08	4.22
34.68	7.73	1.89	1.41	1.43	4.08	4.36
34.70	7.67	1.89	1.40	1.43	4.08	4.50
34.72	7.85	1.89	1.39	1.43	4.08	4.64
34.73	7.96	1.88	1.39	1.42	4.07	4.79
34.75	7.99	1.88	1.39	1.41	4.07	4.95
34.77	7.88	1.88	1.38	1.40	4.07	5.11
34.78	7.73	1.87	1.38	1.39	4.07	5.28
34.80	7.79	1.87	1.37	1.39	4.07	5.46
34.82	7.84	1.86	1.37	1.39	4.06	5.64
34.83	7.87	1.86	1.36	1.38	4.06	5.82
34.85	7.99	1.86	1.35	1.38	4.06	6.02
34.87	8.04	1.86	1.35	1.37	4.05	6.21
34.88	7.98	1.85	1.34	1.37	4.05	6.42
34.90	7.98	1.85	1.34	1.36	4.04	6.63
34.92	7.99	1.84	1.33	1.36	4.04	6.84
34.93	7.90	1.84	1.33	1.35	4.03	7.06
34.95	7.76	1.84	1.32	1.35	4.02	7.30
34.97	7.73	1.84	1.32	1.34	4.02	7.54
34.98	7.91	1.84	1.31	1.33	4.01	7.78
35.00	7.96	1.84	1.30	1.33	4.01	8.03
35.02	7.81	1.84	1.30	1.33	4.00	8.29
35.03	7.76	1.83	1.29	1.32	3.98	8.56
35.05	7.81	1.83	1.29	1.32	3.98	8.83

35.07	7.96	1.82	1.28	1.31	3.96	9.11
35.08	8.15	1.82	1.28	1.31	3.95	9.40
35.10	8.07	1.82	1.27	1.31	3.94	9.70
35.12	7.84	1.82	1.27	1.30	3.93	10.00
35.13	7.79	1.82	1.27	1.29	3.92	10.31
35.15	7.85	1.81	1.26	1.29	3.90	10.63
35.17	7.91	1.81	1.26	1.28	3.89	10.96
35.18	7.91	1.81	1.25	1.28	3.88	11.30
35.20	7.87	1.80	1.25	1.27	3.86	11.64
35.22	7.82	1.80	1.24	1.27	3.85	12.00
35.23	7.82	1.80	1.24	1.27	3.83	12.36
35.25	7.77	1.80	1.23	1.26	3.82	12.74
35.27	7.77	1.79	1.23	1.25	3.80	13.12
35.28	7.94	1.79	1.22	1.25	3.79	13.50
35.30	8.04	1.79	1.22	1.25	3.77	13.89
35.32	7.90	1.78	1.21	1.24	3.76	14.30
35.33	7.74	1.78	1.21	1.24	3.74	14.72
35.35	7.81	1.78	1.21	1.23	3.72	15.14
35.37	7.90	1.78	1.21	1.23	3.70	15.58
35.38	7.90	1.77	1.20	1.23	3.69	16.02
35.40	7.90	1.77	1.20	1.22	3.67	16.47
35.42	7.85	1.76	1.19	1.22	3.66	16.92
35.43	7.87	1.76	1.19	1.21	3.64	17.39
35.45	7.98	1.76	1.18	1.21	3.63	17.86
35.47	7.93	1.76	1.18	1.20	3.61	18.35
35.48	7.91	1.75	1.18	1.20	3.59	18.84
35.50	7.96	1.75	1.17	1.19	3.58	19.34
35.52	7.98	1.75	1.17	1.19	3.56	19.85
35.53	7.91	1.74	1.16	1.19	3.55	20.37
35.55	7.82	1.74	1.16	1.18	3.54	20.89
35.57	7.84	1.74	1.15	1.18	3.52	21.43
35.58	7.85	1.74	1.15	1.17	3.50	21.99
35.60	7.81	1.73	1.15	1.17	3.49	22.55
35.62	7.88	1.73	1.14	1.17	3.47	23.11
35.63	7.88	1.73	1.14	1.16	3.46	23.69
35.65	7.81	1.72	1.14	1.16	3.45	24.27
35.67	7.84	1.72	1.13	1.16	3.43	24.84
35.68	7.85	1.71	1.13	1.15	3.42	25.43
35.70	7.74	1.71	1.13	1.15	3.40	26.03
35.72	7.70	1.71	1.12	1.14	3.39	26.64
35.73	7.84	1.70	1.12	1.14	3.38	27.25
35.75	7.93	1.70	1.12	1.14	3.37	27.88
35.77	7.91	1.70	1.11	1.13	3.36	28.51
35.78	7.77	1.70	1.11	1.13	3.34	29.16
35.80	7.67	1.69	1.10	1.13	3.33	29.82
35.82	7.76	1.68	1.10	1.12	3.32	30.47
35.83	7.94	1.68	1.10	1.12	3.31	31.13
35.85	8.04	1.68	1.09	1.11	3.30	31.79
35.87	7.99	1.68	1.09	1.11	3.29	32.46
35.88	7.90	1.67	1.09	1.11	3.28	33.14
35.90	7.79	1.67	1.09	1.10	3.27	33.83
35.92	7.76	1.67	1.08	1.10	3.26	34.52
35.93	7.76	1.66	1.08	1.09	3.25	35.22
35.95	7.87	1.66	1.07	1.09	3.24	35.92
35.97	8.04	1.65	1.07	1.09	3.23	36.63
35.98	8.05	1.65	1.07	1.09	3.22	37.34
36.00	8.02	1.65	1.06	1.08	3.21	38.06
36.02	7.93	1.64	1.06	1.08	3.20	38.78
36.03	7.91	1.64	1.06	1.07	3.19	39.49
36.05	7.96	1.64	1.06	1.07	3.18	40.22
36.07	7.91	1.64	1.05	1.07	3.17	40.96
36.08	7.85	1.63	1.05	1.06	3.17	41.70
36.10	7.81	1.63	1.05	1.06	3.16	42.43
36.12	7.81	1.62	1.05	1.06	3.15	43.18
36.13	7.77	1.62	1.04	1.05	3.14	43.92
36.15	7.70	1.62	1.04	1.05	3.14	44.67
36.17	7.76	1.61	1.04	1.04	3.13	45.42
36.18	7.73	1.61	1.03	1.04	3.12	46.17
36.20	7.65	1.60	1.03	1.04	3.11	46.92

36.22	7.67	1.60	1.03	1.04	3.10	47.68
36.23	7.65	1.59	1.02	1.03	3.10	48.44
36.25	7.59	1.59	1.02	1.03	3.09	49.20
36.27	7.60	1.59	1.02	1.02	3.08	49.95
36.28	7.56	1.59	1.02	1.02	3.07	50.71
36.30	7.59	1.58	1.02	1.01	3.07	51.48
36.32	7.68	1.57	1.01	1.01	3.06	52.24
36.33	7.70	1.57	1.01	1.01	3.06	52.99
36.35	7.68	1.57	1.00	1.01	3.05	53.75
36.37	7.74	1.56	1.00	1.00	3.05	54.51
36.38	7.76	1.56	1.00	1.00	3.04	55.27
36.40	7.65	1.56	1.00	1.00	3.03	56.03
36.42	7.62	1.56	1.00	0.99	3.03	56.78
36.43	7.68	1.55	0.99	0.99	3.02	57.54
36.45	7.62	1.55	0.99	0.99	3.02	58.30
36.47	7.56	1.55	0.99	0.98	3.01	59.05
36.48	7.60	1.55	0.98	0.98	3.00	59.80
36.50	7.64	1.54	0.98	0.98	3.00	60.55
36.52	7.65	1.54	0.98	0.97	2.99	61.28
36.53	7.67	1.53	0.98	0.97	2.99	62.02
36.55	7.64	1.53	0.98	0.96	2.98	62.76
36.57	7.57	1.53	0.98	0.96	2.97	63.49
36.58	7.60	1.53	0.97	0.96	2.97	64.21
36.60	7.73	1.52	0.97	0.96	2.96	64.93
36.62	7.71	1.52	0.97	0.96	2.96	65.65
36.63	7.57	1.52	0.96	0.95	2.95	66.36
36.65	7.60	1.51	0.96	0.95	2.95	67.08
36.67	7.70	1.51	0.96	0.95	2.94	67.79
36.68	7.68	1.51	0.96	0.94	2.94	68.49
36.70	7.64	1.50	0.96	0.94	2.94	69.18
36.72	7.51	1.50	0.96	0.93	2.93	69.87
36.73	7.59	1.50	0.95	0.93	2.92	70.56
36.75	7.67	1.49	0.95	0.93	2.92	71.24
36.77	7.60	1.49	0.95	0.92	2.91	71.91
36.78	7.57	1.49	0.95	0.92	2.91	72.58
36.80	7.57	1.49	0.94	0.92	2.90	73.23
36.82	7.56	1.48	0.94	0.92	2.90	73.88
36.83	7.53	1.47	0.94	0.91	2.90	74.53
36.85	7.45	1.47	0.94	0.91	2.89	75.18
36.87	7.51	1.48	0.94	0.90	2.89	75.81
36.88	7.64	1.48	0.94	0.90	2.88	76.44
36.90	7.68	1.47	0.93	0.90	2.88	77.05
36.92	7.64	1.47	0.93	0.89	2.87	77.66
36.93	7.56	1.47	0.93	0.89	2.87	78.27
36.95	7.53	1.46	0.93	0.89	2.87	78.86
36.97	7.53	1.46	0.93	0.88	2.86	79.45
36.98	7.51	1.46	0.92	0.88	2.86	80.02
37.00	7.53	1.45	0.92	0.88	2.86	80.60
37.02	7.62	1.45	0.92	0.88	2.85	81.17
37.03	7.67	1.45	0.92	0.87	2.85	81.72
37.05	7.68	1.44	0.92	0.87	2.84	82.26
37.07	7.68	1.44	0.92	0.87	2.84	82.80
37.08	7.54	1.43	0.91	0.86	2.83	83.34
37.10	7.45	1.43	0.91	0.86	2.83	83.86
37.12	7.45	1.43	0.91	0.86	2.83	84.37
37.13	7.43	1.43	0.91	0.86	2.82	84.87
37.15	7.48	1.43	0.91	0.85	2.82	85.36
37.17	7.57	1.42	0.90	0.85	2.81	85.85
37.18	7.68	1.42	0.90	0.84	2.80	86.33
37.20	7.68	1.42	0.90	0.84	2.80	86.81
37.22	7.48	1.41	0.90	0.84	2.80	87.27
37.23	7.31	1.41	0.90	0.83	2.79	87.72
37.25	7.33	1.41	0.90	0.83	2.79	88.16
37.27	7.36	1.41	0.89	0.83	2.79	88.59
37.28	7.39	1.41	0.89	0.83	2.78	89.02
37.30	7.53	1.40	0.89	0.83	2.78	89.43
37.32	7.65	1.40	0.89	0.82	2.77	89.84
37.33	7.68	1.40	0.89	0.82	2.77	90.23
37.35	7.70	1.40	0.89	0.81	2.76	90.63

biomedicines

37.37	7.71	1.39	0.89	0.81	2.76	91.01
37.38	7.71	1.39	0.88	0.81	2.76	91.38
37.40	7.64	1.39	0.88	0.80	2.75	91.74
37.42	7.53	1.38	0.88	0.80	2.75	92.10
37.43	7.48	1.38	0.88	0.80	2.75	92.45
37.45	7.47	1.38	0.88	0.80	2.74	92.78
37.47	7.54	1.38	0.88	0.79	2.74	93.10
37.48	7.59	1.38	0.88	0.79	2.73	93.42
37.50	7.53	1.38	0.88	0.79	2.73	93.74
37.52	7.47	1.37	0.88	0.78	2.72	94.04
37.53	7.40	1.37	0.87	0.78	2.72	94.33
37.55	7.42	1.37	0.87	0.78	2.72	94.61
37.57	7.42	1.36	0.87	0.77	2.71	94.89
37.58	7.47	1.36	0.87	0.77	2.71	95.16
37.60	7.59	1.36	0.87	0.76	2.70	95.42
37.62	7.59	1.36	0.87	0.76	2.70	95.67
37.63	7.59	1.35	0.87	0.76	2.69	95.92
37.65	7.64	1.35	0.86	0.75	2.69	96.17
37.67	7.50	1.34	0.86	0.75	2.69	96.40
37.68	7.48	1.35	0.86	0.75	2.68	96.61
37.70	7.54	1.34	0.86	0.74	2.68	96.82
37.72	7.48	1.34	0.86	0.74	2.67	97.02
37.73	7.50	1.34	0.86	0.73	2.67	97.21
37.75	7.59	1.34	0.86	0.73	2.67	97.40
37.77	7.71	1.34	0.86	0.73	2.66	97.58
37.78	7.76	1.33	0.86	0.73	2.65	97.76
37.80	7.59	1.33	0.86	0.72	2.65	97.94
37.82	7.57	1.32	0.86	0.72	2.64	98.10
37.83	7.67	1.32	0.86	0.71	2.64	98.24
37.85	7.54	1.32	0.85	0.71	2.64	98.38
37.87	7.37	1.32	0.85	0.71	2.64	98.52
37.88	7.37	1.32	0.85	0.71	2.63	98.65
37.90	7.48	1.31	0.85	0.70	2.63	98.78
37.92	7.54	1.31	0.85	0.70	2.62	98.90
37.93	7.56	1.31	0.85	0.70	2.62	99.01
37.95	7.65	1.31	0.85	0.70	2.61	99.12
37.97	7.60	1.30	0.85	0.69	2.61	99.23
37.98	7.54	1.30	0.84	0.69	2.60	99.32
38.00	7.53	1.30	0.84	0.69	2.60	99.41
38.02	7.54	1.30	0.84	0.68	2.60	99.49
38.03	7.59	1.30	0.84	0.68	2.59	99.55
38.05	7.64	1.29	0.84	0.68	2.59	99.61
38.07	7.71	1.29	0.84	0.67	2.58	99.67
38.08	7.71	1.28	0.84	0.67	2.58	99.72
38.10	7.65	1.28	0.84	0.67	2.57	99.78
38.12	7.56	1.28	0.84	0.67	2.57	99.83
38.13	7.59	1.27	0.84	0.66	2.56	99.87
38.15	7.70	1.27	0.84	0.66	2.56	99.90
38.17	7.68	1.27	0.84	0.66	2.56	99.93
38.18	7.64	1.27	0.84	0.66	2.55	99.96
38.20	7.73	1.27	0.84	0.65	2.55	99.98
38.22	7.73	1.27	0.84	0.65	2.55	99.99
38.23	7.62	1.27	0.83	0.64	2.54	100.00
38.25	7.67	1.26	0.83	0.64	2.54	100.00
38.27	7.68	1.26	0.83	0.64	2.53	99.99
38.28	7.62	1.26	0.83	0.64	2.53	99.98
38.30	7.57	1.25	0.83	0.63	2.52	99.98
38.32	7.54	1.25	0.83	0.63	2.52	99.96
38.33	7.51	1.25	0.83	0.63	2.51	99.94
38.35	7.54	1.25	0.83	0.63	2.51	99.91
38.37	7.59	1.25	0.82	0.62	2.51	99.88
38.38	7.48	1.24	0.82	0.62	2.50	99.85
38.40	7.33	1.24	0.82	0.61	2.50	99.81
38.42	7.31	1.24	0.82	0.61	2.49	99.76
38.43	7.40	1.24	0.82	0.61	2.49	99.72
38.45	7.56	1.23	0.82	0.60	2.48	99.67
38.47	7.65	1.23	0.82	0.60	2.48	99.62
38.48	7.70	1.23	0.82	0.60	2.47	99.56
38.50	7.64	1.23	0.82	0.60	2.47	99.50

38.52	7.59	1.22	0.82	0.59	2.47	99.44
38.53	7.62	1.22	0.82	0.59	2.46	99.37
38.55	7.59	1.22	0.82	0.59	2.46	99.30
38.57	7.56	1.22	0.82	0.58	2.45	99.21
38.58	7.53	1.22	0.82	0.58	2.45	99.13
38.60	7.62	1.22	0.82	0.58	2.45	99.06
38.62	7.74	1.21	0.82	0.57	2.44	98.97
38.63	7.79	1.21	0.81	0.57	2.43	98.88
38.65	7.77	1.21	0.81	0.57	2.43	98.79
38.67	7.74	1.21	0.81	0.57	2.42	98.69
38.68	7.68	1.20	0.81	0.57	2.42	98.59
38.70	7.62	1.20	0.81	0.56	2.42	98.49
38.72	7.62	1.20	0.81	0.56	2.41	98.38
38.73	7.59	1.20	0.81	0.56	2.41	98.28
38.75	7.60	1.20	0.81	0.56	2.40	98.17
38.77	7.67	1.19	0.81	0.55	2.40	98.05
38.78	7.51	1.19	0.81	0.55	2.39	97.94
38.80	7.39	1.19	0.81	0.55	2.39	97.82
38.82	7.50	1.19	0.80	0.55	2.38	97.70
38.83	7.57	1.19	0.80	0.54	2.38	97.57
38.85	7.60	1.19	0.80	0.54	2.37	97.44
38.87	7.59	1.19	0.80	0.54	2.37	97.31
38.88	7.59	1.18	0.80	0.54	2.36	97.17
38.90	7.67	1.18	0.80	0.53	2.36	97.04
38.92	7.71	1.17	0.80	0.53	2.35	96.90
38.93	7.60	1.17	0.80	0.53	2.35	96.77
38.95	7.59	1.17	0.80	0.52	2.35	96.63
38.97	7.57	1.17	0.80	0.52	2.34	96.48
38.98	7.47	1.16	0.79	0.52	2.34	96.33
39.00	7.54	1.16	0.79	0.52	2.34	96.18
39.02	7.59	1.16	0.79	0.51	2.33	96.03
39.03	7.62	1.16	0.79	0.51	2.33	95.88
39.05	7.65	1.16	0.79	0.51	2.32	95.73
39.07	7.64	1.15	0.79	0.51	2.32	95.58
39.08	7.65	1.15	0.79	0.50	2.31	95.41
39.10	7.64	1.15	0.79	0.50	2.31	95.25
39.12	7.59	1.15	0.79	0.50	2.30	95.09
39.13	7.59	1.15	0.79	0.50	2.30	94.93
39.15	7.57	1.14	0.79	0.49	2.30	94.77
39.17	7.59	1.15	0.78	0.49	2.29	94.60
39.18	7.68	1.15	0.78	0.49	2.28	94.42
39.20	7.71	1.14	0.78	0.49	2.28	94.26
39.22	7.71	1.14	0.78	0.48	2.28	94.10
39.23	7.64	1.14	0.78	0.48	2.27	93.92
39.25	7.54	1.13	0.78	0.48	2.27	93.74
39.27	7.57	1.13	0.78	0.48	2.27	93.57
39.28	7.50	1.13	0.78	0.48	2.26	93.40
39.30	7.37	1.13	0.78	0.48	2.25	93.22
39.32	7.57	1.13	0.77	0.47	2.25	93.05
39.33	7.79	1.12	0.77	0.47	2.25	92.87
39.35	7.76	1.12	0.77	0.47	2.24	92.69
39.37	7.64	1.12	0.77	0.46	2.24	92.50
39.38	7.57	1.11	0.77	0.46	2.23	92.32
39.40	7.65	1.11	0.77	0.46	2.23	92.13
39.42	7.70	1.12	0.77	0.46	2.22	91.94
39.43	7.60	1.11	0.77	0.45	2.22	91.75
39.45	7.45	1.11	0.77	0.45	2.22	91.56
39.47	7.43	1.11	0.77	0.45	2.21	91.36
39.48	7.50	1.11	0.77	0.45	2.21	91.17
39.50	7.45	1.11	0.76	0.44	2.21	90.97
39.52	7.43	1.11	0.76	0.44	2.20	90.77
39.53	7.51	1.10	0.76	0.43	2.20	90.57
39.55	7.51	1.10	0.76	0.43	2.19	90.37
39.57	7.51	1.10	0.76	0.43	2.18	90.18
39.58	7.48	1.09	0.76	0.43	2.18	89.99
39.60	7.50	1.09	0.76	0.43	2.18	89.79
39.62	7.48	1.09	0.76	0.43	2.17	89.59
39.63	7.50	1.09	0.75	0.42	2.17	89.39
39.65	7.48	1.09	0.75	0.42	2.17	89.18

39.67	7.40	1.09	0.75	0.42	2.16	88.98
39.68	7.43	1.08	0.75	0.42	2.16	88.78
39.70	7.57	1.08	0.75	0.42	2.15	88.57
39.72	7.59	1.08	0.75	0.41	2.15	88.37
39.73	7.57	1.08	0.75	0.41	2.14	88.17
39.75	7.57	1.08	0.75	0.41	2.14	87.97
39.77	7.59	1.08	0.75	0.41	2.14	87.75
39.78	7.62	1.07	0.74	0.40	2.13	87.55
39.80	7.62	1.07	0.75	0.40	2.13	87.34
39.82	7.59	1.07	0.75	0.40	2.12	87.13
39.83	7.57	1.07	0.74	0.39	2.12	86.92
39.85	7.56	1.07	0.74	0.39	2.11	86.70
39.87	7.54	1.07	0.74	0.39	2.11	86.48
39.88	7.62	1.07	0.74	0.39	2.11	86.27
39.90	7.70	1.06	0.74	0.39	2.10	86.06
39.92	7.73	1.06	0.74	0.39	2.10	85.85
39.93	7.73	1.06	0.74	0.39	2.09	85.64
39.95	7.68	1.06	0.73	0.38	2.09	85.44
39.97	7.67	1.05	0.73	0.38	2.08	85.23
39.98	7.68	1.05	0.73	0.38	2.08	85.02
40.00	7.67	1.05	0.73	0.37	2.07	84.81
40.02	7.56	1.05	0.73	0.37	2.07	84.59
40.03	7.53	1.05	0.73	0.37	2.07	84.37
40.05	7.56	1.05	0.73	0.37	2.06	84.16
40.07	7.54	1.05	0.73	0.37	2.06	83.95
40.08	7.50	1.05	0.72	0.36	2.06	83.73
40.10	7.48	1.04	0.72	0.36	2.06	83.51
40.12	7.47	1.04	0.72	0.36	2.05	83.30
40.13	7.47	1.04	0.72	0.36	2.04	83.08
40.15	7.56	1.04	0.72	0.35	2.04	82.86
40.17	7.60	1.04	0.72	0.35	2.03	82.64
40.18	7.70	1.04	0.72	0.35	2.03	82.43
40.20	7.77	1.04	0.72	0.35	2.03	82.21
40.22	7.73	1.04	0.72	0.35	2.03	81.99
40.23	7.65	1.03	0.72	0.34	2.02	81.77
40.25	7.50	1.03	0.71	0.34	2.02	81.55
40.27	7.37	1.03	0.71	0.34	2.02	81.33
40.28	7.36	1.02	0.71	0.33	2.01	81.12
40.30	7.43	1.02	0.71	0.33	2.01	80.91
40.32	7.62	1.02	0.71	0.33	2.00	80.70
40.33	7.68	1.02	0.71	0.33	2.00	80.47
40.35	7.57	1.02	0.71	0.32	1.99	80.25
40.37	7.57	1.02	0.71	0.32	1.99	80.04
40.38	7.59	1.02	0.70	0.32	1.99	79.83
40.40	7.43	1.02	0.70	0.32	1.98	79.62
40.42	7.47	1.02	0.70	0.32	1.98	79.39
40.43	7.59	1.02	0.70	0.32	1.98	79.18
40.45	7.54	1.01	0.70	0.31	1.97	78.96
40.47	7.39	1.01	0.70	0.31	1.97	78.74
40.48	7.30	1.01	0.70	0.31	1.96	78.51
40.50	7.33	1.01	0.70	0.31	1.96	78.29
40.52	7.34	1.00	0.69	0.31	1.96	78.07
40.53	7.45	1.00	0.70	0.31	1.95	77.86
40.55	7.62	1.00	0.70	0.31	1.95	77.64
40.57	7.67	1.00	0.69	0.30	1.95	77.43
40.58	7.53	1.00	0.69	0.30	1.94	77.21
40.60	7.47	1.00	0.69	0.29	1.94	76.99
40.62	7.50	0.99	0.69	0.29	1.93	76.78
40.63	7.50	0.99	0.69	0.29	1.93	76.57
40.65	7.59	0.99	0.69	0.29	1.93	76.36
40.67	7.65	0.99	0.68	0.29	1.92	76.14
40.68	7.62	0.99	0.68	0.28	1.92	75.92
40.70	7.57	0.99	0.68	0.28	1.92	75.70
40.72	7.53	0.99	0.68	0.28	1.91	75.49
40.73	7.45	0.99	0.68	0.28	1.91	75.28
40.75	7.48	0.99	0.68	0.27	1.90	75.06
40.77	7.59	0.98	0.68	0.27	1.90	74.84
40.78	7.68	0.99	0.68	0.26	1.90	74.62
40.80	7.68	0.99	0.68	0.26	1.90	74.40

40.82	7.62	0.99	0.68	0.26	1.89	74.19
40.83	7.51	0.98	0.67	0.26	1.89	73.98
40.85	7.50	0.98	0.67	0.26	1.89	73.76
40.87	7.73	0.98	0.67	0.26	1.88	73.55
40.88	7.84	0.98	0.67	0.25	1.88	73.34
40.90	7.68	0.98	0.67	0.25	1.88	73.13
40.92	7.64	0.98	0.67	0.25	1.87	72.92
40.93	7.73	0.98	0.67	0.25	1.87	72.71
40.95	7.74	0.97	0.67	0.25	1.86	72.49
40.97	7.62	0.97	0.67	0.24	1.86	72.28
40.98	7.57	0.97	0.67	0.24	1.86	72.06
41.00	7.59	0.97	0.67	0.24	1.85	71.85
41.02	7.45	0.97	0.66	0.23	1.85	71.63
41.03	7.31	0.97	0.66	0.23	1.85	71.42
41.05	7.42	0.97	0.66	0.23	1.85	71.20
41.07	7.56	0.97	0.66	0.23	1.84	70.98
41.08	7.60	0.97	0.66	0.23	1.84	70.77
41.10	7.54	0.96	0.66	0.23	1.84	70.57
41.12	7.47	0.96	0.65	0.22	1.83	70.36
41.13	7.56	0.96	0.65	0.22	1.83	70.15
41.15	7.60	0.96	0.65	0.22	1.83	69.95
41.17	7.47	0.96	0.65	0.21	1.82	69.74
41.18	7.36	0.96	0.65	0.21	1.82	69.54
41.20	7.36	0.96	0.65	0.21	1.82	69.33
41.22	7.43	0.96	0.65	0.21	1.81	69.12
41.23	7.48	0.95	0.64	0.20	1.81	68.92
41.25	7.47	0.95	0.64	0.20	1.81	68.71
41.27	7.39	0.95	0.64	0.20	1.80	68.51
41.28	7.50	0.95	0.64	0.20	1.80	68.30
41.30	7.64	0.95	0.64	0.20	1.80	68.10
41.32	7.59	0.95	0.64	0.19	1.80	67.89
41.33	7.48	0.95	0.64	0.19	1.80	67.69
41.35	7.53	0.95	0.64	0.19	1.79	67.50
41.37	7.67	0.95	0.64	0.19	1.79	67.30
41.38	7.71	0.94	0.64	0.18	1.79	67.10
41.40	7.67	0.94	0.64	0.18	1.78	66.89
41.42	7.59	0.94	0.63	0.18	1.78	66.68
41.43	7.59	0.94	0.63	0.18	1.77	66.48
41.45	7.60	0.94	0.63	0.17	1.77	66.27
41.47	7.60	0.94	0.63	0.17	1.77	66.08
41.48	7.60	0.94	0.63	0.17	1.77	65.88
41.50	7.60	0.94	0.63	0.17	1.77	65.68
41.52	7.59	0.94	0.63	0.17	1.76	65.48
41.53	7.59	0.94	0.63	0.17	1.76	65.28
41.55	7.76	0.93	0.63	0.16	1.76	65.08
41.57	7.85	0.93	0.63	0.16	1.76	64.88
41.58	7.81	0.93	0.63	0.16	1.75	64.68
41.60	7.65	0.93	0.63	0.16	1.75	64.49
41.62	7.43	0.93	0.63	0.15	1.75	64.29
41.63	7.36	0.93	0.63	0.15	1.74	64.10
41.65	7.42	0.92	0.62	0.15	1.74	63.91
41.67	7.48	0.92	0.62	0.14	1.74	63.71
41.68	7.57	0.92	0.62	0.14	1.74	63.52
41.70	7.56	0.92	0.62	0.14	1.73	63.32
41.72	7.60	0.91	0.62	0.14	1.73	63.13
41.73	7.65	0.91	0.62	0.14	1.73	62.94
41.75	7.54	0.91	0.62	0.14	1.73	62.75
41.77	7.50	0.91	0.62	0.13	1.73	62.55
41.78	7.56	0.91	0.61	0.13	1.73	62.36
41.80	7.59	0.91	0.61	0.13	1.72	62.18
41.82	7.51	0.91	0.61	0.13	1.72	62.00
41.83	7.53	0.91	0.61	0.13	1.72	61.80
41.85	7.73	0.91	0.61	0.13	1.71	61.62
41.87	7.74	0.90	0.61	0.12	1.71	61.43
41.88	7.67	0.90	0.61	0.11	1.71	61.24
41.90	7.59	0.90	0.61	0.11	1.71	61.05
41.92	7.54	0.90	0.61	0.11	1.70	60.87
41.93	7.60	0.90	0.61	0.11	1.70	60.69
41.95	7.65	0.90	0.61	0.11	1.70	60.51

41.97	7.65	0.90	0.60	0.11	1.70	60.33
41.98	7.71	0.90	0.60	0.11	1.70	60.15
42.00	7.76	0.89	0.60	0.11	1.70	59.97
42.02	7.71	0.89	0.60	0.10	1.69	59.78
42.03	7.51	0.89	0.60	0.10	1.69	59.60
42.05	7.42	0.89	0.60	0.10	1.69	59.41
42.07	7.47	0.89	0.60	0.09	1.69	59.22
42.08	7.47	0.89	0.60	0.09	1.69	59.04
42.10	7.53	0.88	0.60	0.09	1.68	58.86
42.12	7.68	0.88	0.60	0.09	1.68	58.67
42.13	7.71	0.88	0.60	0.08	1.67	58.49
42.15	7.57	0.88	0.59	0.08	1.67	58.32
42.17	7.51	0.88	0.59	0.08	1.67	58.15
42.18	7.54	0.87	0.59	0.08	1.67	57.97
42.20	7.65	0.87	0.59	0.08	1.67	57.79
42.22	7.74	0.87	0.59	0.08	1.66	57.61
42.23	7.77	0.87	0.59	0.08	1.66	57.44
42.25	7.81	0.86	0.59	0.07	1.66	57.27
42.27	7.68	0.86	0.59	0.07	1.66	57.10
42.28	7.48	0.86	0.59	0.07	1.66	56.92
42.30	7.47	0.86	0.59	0.07	1.65	56.75
42.32	7.57	0.86	0.58	0.07	1.65	56.58
42.33	7.71	0.86	0.58	0.06	1.65	56.41
42.35	7.71	0.86	0.58	0.06	1.65	56.24
42.37	7.62	0.86	0.58	0.06	1.65	56.07
42.38	7.60	0.86	0.58	0.06	1.65	55.89
42.40	7.64	0.85	0.58	0.05	1.65	55.72
42.42	7.70	0.85	0.58	0.05	1.65	55.55
42.43	7.79	0.85	0.58	0.05	1.64	55.38
42.45	7.84	0.85	0.58	0.05	1.64	55.21
42.47	7.76	0.85	0.58	0.05	1.64	55.05
42.48	7.62	0.85	0.58	0.05	1.64	54.88
42.50	7.60	0.84	0.58	0.04	1.64	54.71
42.52	7.74	0.84	0.58	0.04	1.63	54.54
42.53	7.84	0.84	0.57	0.04	1.63	54.37
42.55	7.84	0.84	0.57	0.04	1.63	54.21
42.57	7.84	0.83	0.57	0.04	1.63	54.05
42.58	7.79	0.83	0.57	0.04	1.63	53.89
42.60	7.74	0.83	0.57	0.03	1.62	53.73
42.62	7.73	0.83	0.57	0.03	1.62	53.57
42.63	7.81	0.83	0.57	0.02	1.62	53.41
42.65	7.90	0.83	0.57	0.02	1.62	53.26
42.67	7.91	0.83	0.57	0.03	1.62	53.10
42.68	7.88	0.83	0.57	0.03	1.62	52.94
42.70	7.82	0.82	0.57	0.02	1.62	52.78
42.72	7.74	0.82	0.57	0.02	1.62	52.62
42.73	7.74	0.82	0.56	0.01	1.61	52.46
42.75	7.71	0.82	0.56	0.01	1.61	52.31
42.77	7.68	0.82	0.56	0.02	1.61	52.15
42.78	7.71	0.82	0.56	0.01	1.61	52.00
42.80	7.73	0.81	0.56	0.01	1.60	51.84
42.82	7.73	0.81	0.56	0.01	1.60	51.69
42.83	7.77	0.81	0.56	0.01	1.60	51.53
42.85	7.82	0.80	0.56	0.01	1.60	51.36
42.87	7.79	0.80	0.56	0.01	1.60	51.21
42.88	7.76	0.80	0.56	0.00	1.60	51.06
42.90	7.74	0.80	0.56	0.00	1.59	50.91
42.92	7.73	0.80	0.56	0.00	1.59	50.76
42.93	7.81	0.80	0.56	0.00	1.59	50.61
42.95	7.84	0.80	0.55	-0.01	1.59	50.46
42.97	7.81	0.79	0.55	-0.01	1.59	50.31
42.98	7.74	0.79	0.55	-0.01	1.59	50.16
43.00	7.76	0.79	0.55	-0.01	1.58	50.01
43.02	7.74	0.79	0.55	-0.01	1.58	49.86
43.03	7.74	0.79	0.55	-0.01	1.58	49.73
43.05	7.79	0.79	0.55	-0.01	1.58	49.58
43.07	7.70	0.79	0.55	-0.01	1.58	49.44
43.08	7.60	0.79	0.55	-0.02	1.58	49.29
43.10	7.64	0.79	0.54	-0.02	1.58	49.15

43.12	7.68	0.79	0.54	-0.02	1.58	49.00
43.13	7.77	0.78	0.54	-0.02	1.58	48.85
43.15	7.88	0.78	0.54	-0.03	1.57	48.71
43.17	7.94	0.78	0.54	-0.03	1.57	48.57
43.18	7.85	0.78	0.54	-0.03	1.57	48.43
43.20	7.76	0.78	0.54	-0.03	1.57	48.29
43.22	7.82	0.77	0.54	-0.03	1.57	48.14
43.23	7.88	0.77	0.54	-0.03	1.57	48.00
43.25	7.82	0.77	0.54	-0.03	1.56	47.87
43.27	7.71	0.77	0.54	-0.04	1.56	47.74
43.28	7.76	0.77	0.54	-0.04	1.56	47.60
43.30	7.77	0.77	0.54	-0.04	1.57	47.46
43.32	7.65	0.77	0.54	-0.04	1.56	47.33
43.33	7.54	0.77	0.53	-0.04	1.56	47.20
43.35	7.65	0.76	0.53	-0.05	1.56	47.06
43.37	7.84	0.76	0.53	-0.05	1.56	46.92
43.38	7.87	0.76	0.53	-0.05	1.56	46.79
43.40	7.82	0.76	0.53	-0.05	1.56	46.65
43.42	7.79	0.76	0.53	-0.05	1.55	46.51
43.43	7.82	0.76	0.53	-0.05	1.56	46.38
43.45	7.85	0.76	0.53	-0.05	1.55	46.25
43.47	7.85	0.76	0.53	-0.06	1.55	46.11
43.48	7.93	0.75	0.53	-0.06	1.55	45.98
43.50	7.94	0.75	0.52	-0.06	1.55	45.85
43.52	7.90	0.75	0.52	-0.06	1.55	45.73
43.53	7.90	0.75	0.52	-0.06	1.55	45.60
43.55	7.84	0.75	0.52	-0.06	1.55	45.47
43.57	7.74	0.75	0.52	-0.07	1.55	45.35
43.58	7.73	0.75	0.52	-0.07	1.54	45.22
43.60	7.76	0.75	0.52	-0.08	1.54	45.10
43.62	7.73	0.75	0.52	-0.08	1.54	44.97
43.63	7.67	0.75	0.52	-0.08	1.54	44.84
43.65	7.67	0.74	0.52	-0.08	1.54	44.71
43.67	7.67	0.74	0.52	-0.08	1.54	44.59
43.68	7.73	0.74	0.52	-0.08	1.54	44.47
43.70	7.76	0.74	0.52	-0.09	1.54	44.35
43.72	7.79	0.74	0.52	-0.08	1.54	44.22
43.73	7.91	0.74	0.51	-0.08	1.53	44.10
43.75	8.01	0.74	0.51	-0.09	1.53	43.97
43.77	7.98	0.74	0.51	-0.08	1.53	43.85
43.78	7.84	0.73	0.51	-0.09	1.53	43.72
43.80	7.68	0.73	0.51	-0.09	1.53	43.60
43.82	7.62	0.73	0.51	-0.09	1.53	43.48
43.83	7.81	0.73	0.51	-0.09	1.53	43.36
43.85	7.99	0.73	0.51	-0.09	1.53	43.24
43.87	7.99	0.73	0.51	-0.09	1.53	43.13
43.88	7.81	0.72	0.51	-0.10	1.53	43.01
43.90	7.59	0.72	0.50	-0.10	1.53	42.89
43.92	7.70	0.73	0.50	-0.10	1.53	42.78
43.93	7.91	0.73	0.50	-0.10	1.52	42.66
43.95	7.91	0.72	0.50	-0.10	1.52	42.54
43.97	7.84	0.72	0.50	-0.10	1.52	42.43
43.98	7.88	0.72	0.50	-0.10	1.52	42.31
44.00	7.91	0.72	0.50	-0.11	1.52	42.20
44.02	7.93	0.72	0.50	-0.11	1.52	42.09
44.03	7.84	0.72	0.50	-0.11	1.52	41.98
44.05	7.70	0.72	0.49	-0.11	1.52	41.87
44.07	7.76	0.71	0.49	-0.12	1.52	41.75
44.08	7.85	0.71	0.49	-0.12	1.52	41.64
44.10	7.84	0.71	0.49	-0.12	1.52	41.52
44.12	7.82	0.71	0.49	-0.12	1.51	41.40
44.13	7.84	0.71	0.49	-0.12	1.51	41.30
44.15	7.84	0.71	0.49	-0.13	1.51	41.19
44.17	7.87	0.71	0.49	-0.13	1.51	41.09
44.18	7.90	0.71	0.49	-0.13	1.51	40.98
44.20	7.96	0.71	0.49	-0.13	1.51	40.87
44.22	7.99	0.71	0.49	-0.13	1.51	40.76
44.23	8.01	0.70	0.49	-0.13	1.51	40.66
44.25	8.04	0.70	0.49	-0.13	1.51	40.54

44.27	7.99	0.70	0.48	-0.13	1.51	40.43
44.28	8.01	0.70	0.48	-0.13	1.51	40.33
44.30	8.04	0.70	0.48	-0.14	1.51	40.23
44.32	7.98	0.70	0.48	-0.14	1.51	40.12
44.33	7.93	0.70	0.48	-0.14	1.51	40.00
44.35	7.93	0.70	0.48	-0.14	1.51	39.89
44.37	7.93	0.70	0.48	-0.14	1.51	39.79
44.38	7.98	0.69	0.48	-0.14	1.50	39.69
44.40	7.91	0.69	0.48	-0.14	1.50	39.59
44.42	7.81	0.69	0.48	-0.15	1.50	39.48
44.43	7.73	0.69	0.48	-0.15	1.50	39.38
44.45	7.77	0.69	0.48	-0.15	1.50	39.28
44.47	7.93	0.69	0.48	-0.15	1.50	39.18
44.48	7.99	0.68	0.47	-0.15	1.50	39.08
44.50	7.81	0.68	0.47	-0.15	1.50	38.98
44.52	7.76	0.68	0.47	-0.16	1.50	38.87
44.53	7.88	0.68	0.47	-0.16	1.50	38.78
44.55	7.88	0.68	0.47	-0.16	1.49	38.69
44.57	7.90	0.68	0.47	-0.16	1.49	38.59
44.58	8.01	0.68	0.47	-0.16	1.49	38.50
44.60	7.99	0.68	0.47	-0.16	1.49	38.40
44.62	7.90	0.68	0.47	-0.16	1.50	38.30
44.63	7.91	0.68	0.47	-0.17	1.49	38.20
44.65	8.02	0.68	0.47	-0.17	1.49	38.10
44.67	7.88	0.67	0.47	-0.17	1.49	37.99
44.68	7.73	0.67	0.46	-0.17	1.49	37.90
44.70	7.87	0.67	0.46	-0.17	1.49	37.80
44.72	7.91	0.68	0.46	-0.17	1.49	37.71
44.73	7.93	0.68	0.47	-0.18	1.49	37.61
44.75	8.01	0.68	0.46	-0.18	1.49	37.52
44.77	7.94	0.67	0.46	-0.18	1.49	37.43
44.78	7.84	0.67	0.46	-0.18	1.49	37.34
44.80	7.91	0.67	0.46	-0.18	1.48	37.25
44.82	8.01	0.67	0.46	-0.18	1.48	37.15
44.83	7.96	0.67	0.46	-0.18	1.48	37.07
44.85	7.99	0.67	0.46	-0.18	1.48	36.98
44.87	8.01	0.67	0.46	-0.18	1.48	36.88
44.88	7.90	0.66	0.46	-0.18	1.48	36.78
44.90	7.85	0.66	0.46	-0.18	1.48	36.69
44.92	7.91	0.66	0.46	-0.18	1.48	36.60
44.93	7.93	0.66	0.46	-0.18	1.48	36.50
44.95	7.94	0.66	0.46	-0.19	1.48	36.41
44.97	7.94	0.66	0.46	-0.19	1.48	36.33
44.98	7.87	0.66	0.46	-0.19	1.48	36.24
45.00	7.81	0.66	0.46	-0.19	1.48	36.14
45.02	7.88	0.66	0.46	-0.19	1.48	36.06
45.03	7.99	0.65	0.46	-0.20	1.48	35.97
45.05	8.02	0.65	0.46	-0.20	1.48	35.89
45.07	7.96	0.65	0.46	-0.20	1.48	35.80
45.08	7.87	0.65	0.46	-0.20	1.48	35.71
45.10	7.94	0.65	0.46	-0.20	1.47	35.62
45.12	8.11	0.65	0.46	-0.20	1.48	35.53
45.13	8.19	0.65	0.46	-0.21	1.48	35.44
45.15	8.15	0.65	0.46	-0.21	1.48	35.36
45.17	8.10	0.65	0.46	-0.21	1.48	35.27
45.18	8.05	0.65	0.46	-0.21	1.47	35.18
45.20	8.04	0.65	0.46	-0.21	1.47	35.10
45.22	8.02	0.65	0.46	-0.21	1.47	35.02
45.23	8.04	0.64	0.46	-0.21	1.47	34.94
45.25	8.11	0.64	0.46	-0.22	1.47	34.86
45.27	8.08	0.64	0.46	-0.22	1.47	34.77
45.28	8.01	0.64	0.46	-0.22	1.47	34.68
45.30	8.05	0.64	0.46	-0.22	1.47	34.60
45.32	8.16	0.64	0.46	-0.22	1.47	34.52
45.33	8.11	0.63	0.45	-0.22	1.47	34.44
45.35	8.04	0.63	0.46	-0.22	1.47	34.36
45.37	8.07	0.63	0.46	-0.22	1.46	34.27
45.38	8.18	0.63	0.46	-0.22	1.47	34.19
45.40	8.22	0.63	0.46	-0.22	1.47	34.11

45.42	8.21	0.63	0.46	-0.23	1.47	34.03
45.43	8.16	0.63	0.46	-0.23	1.46	33.96
45.45	8.18	0.63	0.46	-0.24	1.46	33.88
45.47	8.16	0.63	0.46	-0.24	1.46	33.80
45.48	8.13	0.63	0.46	-0.24	1.46	33.72
45.50	8.15	0.63	0.46	-0.24	1.46	33.64
45.52	8.13	0.62	0.46	-0.24	1.46	33.55
45.53	8.15	0.62	0.46	-0.24	1.46	33.47
45.55	8.18	0.62	0.46	-0.24	1.46	33.39
45.57	8.19	0.62	0.46	-0.24	1.46	33.32
45.58	8.16	0.62	0.46	-0.24	1.46	33.23
45.60	8.07	0.62	0.46	-0.24	1.46	33.16
45.62	8.07	0.62	0.46	-0.24	1.46	33.09
45.63	8.07	0.62	0.46	-0.24	1.46	33.01
45.65	7.98	0.62	0.47	-0.24	1.46	32.93
45.67	7.98	0.61	0.47	-0.25	1.46	32.86
45.68	8.11	0.61	0.47	-0.25	1.45	32.77
45.70	8.22	0.61	0.47	-0.25	1.45	32.70
45.72	8.19	0.61	0.47	-0.25	1.45	32.62
45.73	8.13	0.61	0.47	-0.25	1.45	32.55
45.75	8.18	0.61	0.47	-0.25	1.45	32.48
45.77	8.19	0.61	0.47	-0.25	1.45	32.41
45.78	8.13	0.61	0.47	-0.26	1.45	32.34
45.80	8.19	0.61	0.47	-0.26	1.45	32.27
45.82	8.25	0.61	0.48	-0.26	1.45	32.19
45.83	8.19	0.61	0.48	-0.26	1.45	32.11
45.85	8.11	0.61	0.48	-0.26	1.45	32.03
45.87	8.10	0.60	0.48	-0.26	1.45	31.96
45.88	8.07	0.60	0.48	-0.26	1.45	31.89
45.90	8.04	0.60	0.48	-0.27	1.45	31.81
45.92	8.05	0.61	0.48	-0.27	1.45	31.74
45.93	8.15	0.60	0.48	-0.27	1.44	31.67
45.95	8.35	0.60	0.49	-0.27	1.44	31.61
45.97	8.38	0.60	0.49	-0.27	1.44	31.54
45.98	8.21	0.60	0.49	-0.27	1.44	31.47
46.00	8.13	0.60	0.49	-0.27	1.44	31.40
46.02	8.19	0.60	0.49	-0.27	1.44	31.33
46.03	8.15	0.60	0.49	-0.27	1.44	31.27
46.05	7.94	0.60	0.49	-0.27	1.44	31.20
46.07	7.85	0.59	0.49	-0.27	1.44	31.13
46.08	7.90	0.59	0.49	-0.27	1.44	31.07
46.10	8.07	0.59	0.49	-0.28	1.44	31.00
46.12	8.22	0.59	0.50	-0.28	1.44	30.92
46.13	8.15	0.59	0.50	-0.28	1.44	30.84
46.15	7.99	0.59	0.50	-0.28	1.43	30.77
46.17	7.93	0.59	0.50	-0.28	1.43	30.71
46.18	7.88	0.58	0.51	-0.28	1.43	30.64
46.20	8.02	0.59	0.51	-0.28	1.43	30.58
46.22	8.16	0.59	0.51	-0.28	1.43	30.51
46.23	8.13	0.59	0.51	-0.29	1.43	30.44
46.25	8.08	0.59	0.51	-0.29	1.43	30.38
46.27	8.07	0.58	0.52	-0.29	1.43	30.32
46.28	8.01	0.58	0.52	-0.29	1.43	30.25
46.30	8.04	0.58	0.52	-0.29	1.43	30.19
46.32	8.13	0.58	0.52	-0.29	1.43	30.12
46.33	8.18	0.58	0.52	-0.29	1.43	30.05
46.35	8.15	0.58	0.52	-0.29	1.43	29.98
46.37	8.13	0.58	0.53	-0.29	1.43	29.92
46.38	8.32	0.58	0.53	-0.29	1.43	29.86
46.40	8.32	0.58	0.53	-0.30	1.43	29.79
46.42	8.16	0.58	0.53	-0.30	1.42	29.72
46.43	8.11	0.58	0.54	-0.30	1.42	29.67
46.45	8.07	0.58	0.54	-0.30	1.42	29.61
46.47	8.13	0.57	0.54	-0.30	1.42	29.54
46.48	8.25	0.57	0.54	-0.30	1.42	29.47
46.50	8.27	0.57	0.54	-0.31	1.42	29.41
46.52	8.15	0.57	0.54	-0.31	1.42	29.35
46.53	8.07	0.57	0.55	-0.31	1.42	29.28
46.55	8.07	0.57	0.55	-0.31	1.42	29.22

46.57	8.08	0.57	0.55	-0.31	1.42	29.16
46.58	8.10	0.56	0.55	-0.31	1.42	29.10
46.60	8.10	0.56	0.55	-0.31	1.42	29.03
46.62	8.19	0.56	0.56	-0.31	1.41	28.97
46.63	8.13	0.56	0.56	-0.31	1.41	28.91
46.65	8.02	0.56	0.56	-0.31	1.41	28.84
46.67	8.01	0.56	0.56	-0.32	1.41	28.79
46.68	7.99	0.56	0.57	-0.32	1.41	28.72
46.70	7.87	0.56	0.57	-0.32	1.41	28.66
46.72	7.87	0.56	0.57	-0.32	1.41	28.59
46.73	7.93	0.56	0.57	-0.32	1.41	28.54
46.75	7.94	0.56	0.57	-0.32	1.41	28.48
46.77	7.94	0.56	0.58	-0.32	1.41	28.42
46.78	8.10	0.56	0.58	-0.32	1.41	28.37
46.80	8.21	0.56	0.58	-0.32	1.41	28.31
46.82	8.13	0.56	0.58	-0.32	1.40	28.24
46.83	8.11	0.56	0.58	-0.32	1.40	28.18
46.85	8.18	0.56	0.59	-0.33	1.40	28.12
46.87	8.13	0.55	0.59	-0.33	1.40	28.07
46.88	8.01	0.55	0.59	-0.33	1.40	28.02
46.90	8.02	0.55	0.59	-0.33	1.40	27.96
46.92	8.08	0.55	0.59	-0.33	1.40	27.91
46.93	8.02	0.54	0.59	-0.33	1.40	27.85
46.95	7.90	0.54	0.60	-0.33	1.39	27.79
46.97	7.90	0.54	0.60	-0.33	1.39	27.73
46.98	7.91	0.54	0.60	-0.33	1.39	27.67
47.00	7.87	0.54	0.60	-0.33	1.39	27.61
47.02	7.88	0.54	0.60	-0.34	1.39	27.56
47.03	8.01	0.54	0.61	-0.34	1.39	27.51
47.05	8.04	0.54	0.61	-0.34	1.39	27.45
47.07	7.84	0.54	0.61	-0.34	1.39	27.39
47.08	7.71	0.54	0.61	-0.34	1.39	27.33
47.10	7.79	0.54	0.62	-0.34	1.39	27.28
47.12	7.87	0.54	0.62	-0.34	1.39	27.22
47.13	7.84	0.53	0.62	-0.34	1.39	27.17
47.15	7.82	0.53	0.62	-0.34	1.39	27.11
47.17	7.87	0.54	0.62	-0.34	1.39	27.06
47.18	7.93	0.54	0.62	-0.34	1.39	27.01
47.20	7.99	0.54	0.62	-0.34	1.38	26.95
47.22	8.02	0.54	0.62	-0.35	1.38	26.90
47.23	8.01	0.54	0.62	-0.35	1.38	26.84
47.25	7.91	0.53	0.63	-0.35	1.38	26.79
47.27	7.94	0.53	0.63	-0.35	1.38	26.73
47.28	8.10	0.53	0.63	-0.35	1.38	26.68
47.30	8.15	0.53	0.63	-0.35	1.38	26.63
47.32	7.96	0.53	0.63	-0.35	1.38	26.57
47.33	7.84	0.53	0.63	-0.35	1.38	26.52
47.35	7.99	0.53	0.63	-0.35	1.37	26.47
47.37	8.22	0.53	0.63	-0.35	1.37	26.41
47.38	8.21	0.53	0.64	-0.35	1.37	26.36
47.40	8.10	0.53	0.64	-0.35	1.37	26.32
47.42	8.08	0.52	0.64	-0.36	1.37	26.26
47.43	7.99	0.52	0.64	-0.36	1.37	26.21
47.45	7.94	0.52	0.64	-0.36	1.37	26.15
47.47	8.02	0.52	0.64	-0.36	1.37	26.10
47.48	7.98	0.52	0.64	-0.36	1.37	26.04
47.50	7.93	0.52	0.64	-0.36	1.37	25.99
47.52	7.98	0.52	0.65	-0.36	1.37	25.94
47.53	8.02	0.52	0.65	-0.36	1.37	25.89
47.55	8.02	0.52	0.65	-0.36	1.37	25.84
47.57	7.91	0.52	0.65	-0.36	1.37	25.79
47.58	7.88	0.52	0.65	-0.36	1.37	25.74
47.60	7.99	0.52	0.65	-0.36	1.37	25.69
47.62	8.07	0.52	0.65	-0.37	1.36	25.64
47.63	7.99	0.52	0.65	-0.37	1.37	25.59
47.65	7.88	0.51	0.65	-0.37	1.37	25.53
47.67	7.99	0.51	0.65	-0.37	1.37	25.48
47.68	8.10	0.51	0.65	-0.37	1.36	25.43
47.70	8.07	0.51	0.65	-0.38	1.36	25.38

47.72	8.05	0.51	0.65	-0.37	1.36	25.33
47.73	8.02	0.51	0.65	-0.37	1.36	25.29
47.75	7.96	0.51	0.65	-0.37	1.36	25.25
47.77	7.94	0.51	0.65	-0.37	1.36	25.19
47.78	7.99	0.50	0.65	-0.38	1.36	25.14
47.80	8.08	0.50	0.65	-0.38	1.36	25.09
47.82	8.08	0.50	0.65	-0.38	1.36	25.05
47.83	8.01	0.50	0.65	-0.38	1.36	25.00
47.85	7.99	0.50	0.65	-0.38	1.36	24.95
47.87	7.98	0.50	0.65	-0.38	1.36	24.91
47.88	7.84	0.50	0.65	-0.38	1.36	24.85
47.90	7.76	0.50	0.65	-0.38	1.36	24.81
47.92	7.90	0.50	0.65	-0.38	1.36	24.76
47.93	8.04	0.50	0.65	-0.38	1.36	24.71
47.95	8.07	0.50	0.65	-0.38	1.36	24.65
47.97	7.94	0.50	0.65	-0.38	1.36	24.61
47.98	7.81	0.50	0.65	-0.38	1.36	24.57
48.00	7.88	0.50	0.65	-0.38	1.36	24.53
48.02	7.99	0.50	0.65	-0.38	1.36	24.49
48.03	8.04	0.49	0.65	-0.39	1.36	24.44
48.05	8.05	0.49	0.65	-0.39	1.36	24.39
48.07	7.96	0.49	0.65	-0.39	1.36	24.35
48.08	7.96	0.49	0.65	-0.39	1.36	24.29
48.10	8.10	0.49	0.65	-0.39	1.36	24.24
48.12	8.08	0.49	0.65	-0.39	1.36	24.20
48.13	7.91	0.49	0.64	-0.40	1.36	24.16
48.15	7.79	0.49	0.64	-0.39	1.36	24.12
48.17	7.77	0.48	0.64	-0.39	1.36	24.07
48.18	7.87	0.49	0.65	-0.40	1.36	24.03
48.20	7.94	0.49	0.65	-0.40	1.36	23.98
48.22	7.94	0.48	0.64	-0.40	1.37	23.94
48.23	7.93	0.48	0.64	-0.39	1.37	23.89
48.25	7.94	0.48	0.64	-0.40	1.37	23.84
48.27	7.91	0.49	0.64	-0.40	1.36	23.80
48.28	7.87	0.48	0.64	-0.40	1.36	23.76
48.30	7.84	0.48	0.64	-0.41	1.36	23.72
48.32	7.76	0.48	0.64	-0.41	1.37	23.67
48.33	7.73	0.48	0.64	-0.41	1.37	23.63
48.35	7.88	0.48	0.64	-0.41	1.37	23.59
48.37	8.05	0.48	0.63	-0.41	1.37	23.55
48.38	8.01	0.47	0.63	-0.41	1.36	23.50
48.40	7.93	0.47	0.63	-0.41	1.36	23.45
48.42	8.02	0.48	0.63	-0.41	1.36	23.40
48.43	8.05	0.48	0.63	-0.41	1.36	23.36
48.45	8.02	0.48	0.63	-0.41	1.36	23.32
48.47	8.02	0.47	0.63	-0.41	1.36	23.28
48.48	7.94	0.47	0.63	-0.41	1.36	23.24
48.50	7.76	0.47	0.62	-0.41	1.36	23.20
48.52	7.68	0.47	0.62	-0.41	1.36	23.16
48.53	7.84	0.47	0.62	-0.42	1.36	23.11
48.55	7.93	0.47	0.62	-0.42	1.36	23.07
48.57	7.94	0.47	0.62	-0.42	1.36	23.03
48.58	7.91	0.47	0.61	-0.42	1.36	22.99
48.60	7.90	0.47	0.61	-0.42	1.36	22.94
48.62	7.90	0.47	0.61	-0.42	1.36	22.89
48.63	7.90	0.47	0.61	-0.42	1.36	22.85
48.65	7.88	0.46	0.61	-0.42	1.36	22.82
48.67	7.82	0.46	0.61	-0.42	1.36	22.78
48.68	7.81	0.46	0.61	-0.42	1.36	22.73
48.70	7.93	0.46	0.61	-0.42	1.36	22.69
48.72	8.01	0.46	0.60	-0.42	1.36	22.65
48.73	8.02	0.46	0.60	-0.42	1.36	22.60
48.75	7.93	0.46	0.60	-0.43	1.36	22.55
48.77	7.65	0.46	0.60	-0.43	1.36	22.52
48.78	7.53	0.46	0.60	-0.43	1.36	22.49
48.80	7.65	0.46	0.59	-0.43	1.36	22.45
48.82	7.88	0.46	0.59	-0.43	1.36	22.41
48.83	7.98	0.46	0.59	-0.43	1.36	22.37
48.85	7.93	0.46	0.59	-0.43	1.36	22.33

48.87	7.85	0.46	0.59	-0.43	1.36	22.28
48.88	7.74	0.46	0.58	-0.43	1.36	22.23
48.90	7.81	0.45	0.58	-0.43	1.36	22.19
48.92	7.84	0.45	0.58	-0.44	1.36	22.16
48.93	7.90	0.46	0.58	-0.44	1.36	22.12
48.95	7.98	0.45	0.58	-0.44	1.36	22.08
48.97	7.87	0.45	0.58	-0.44	1.36	22.04
48.98	7.77	0.45	0.57	-0.44	1.36	22.00
49.00	7.81	0.45	0.57	-0.45	1.36	21.96
49.02	7.82	0.45	0.57	-0.45	1.36	21.92
49.03	7.88	0.45	0.57	-0.45	1.36	21.88
49.05	7.96	0.45	0.56	-0.45	1.36	21.83
49.07	7.98	0.45	0.56	-0.45	1.36	21.80
49.08	7.88	0.44	0.56	-0.45	1.36	21.77
49.10	7.81	0.44	0.56	-0.45	1.36	21.73
49.12	7.77	0.44	0.55	-0.45	1.36	21.69
49.13	7.84	0.44	0.55	-0.45	1.36	21.66
49.15	7.88	0.44	0.55	-0.45	1.36	21.63
49.17	7.84	0.44	0.55	-0.45	1.36	21.58
49.18	7.84	0.44	0.54	-0.45	1.36	21.54
49.20	7.90	0.43	0.54	-0.46	1.36	21.50
49.22	7.98	0.43	0.54	-0.46	1.36	21.47
49.23	7.90	0.43	0.54	-0.46	1.36	21.43
49.25	7.79	0.43	0.54	-0.46	1.36	21.39
49.27	7.88	0.43	0.53	-0.46	1.36	21.34
49.28	8.02	0.43	0.53	-0.46	1.36	21.31
49.30	8.02	0.43	0.53	-0.46	1.36	21.28
49.32	8.04	0.43	0.52	-0.46	1.36	21.25
49.33	8.08	0.42	0.52	-0.47	1.36	21.21
49.35	7.94	0.42	0.52	-0.47	1.36	21.17
49.37	7.85	0.42	0.52	-0.47	1.36	21.14
49.38	7.93	0.42	0.52	-0.47	1.36	21.10
49.40	7.87	0.42	0.52	-0.47	1.36	21.07
49.42	7.74	0.41	0.51	-0.47	1.36	21.03
49.43	7.81	0.42	0.51	-0.47	1.36	20.99
49.45	7.90	0.42	0.51	-0.47	1.36	20.95
49.47	7.91	0.41	0.50	-0.47	1.36	20.92
49.48	7.90	0.41	0.50	-0.47	1.36	20.89
49.50	7.90	0.41	0.50	-0.48	1.36	20.86
49.52	7.88	0.41	0.50	-0.48	1.36	20.82
49.53	7.77	0.41	0.49	-0.47	1.36	20.77
49.55	7.64	0.41	0.49	-0.48	1.36	20.74
49.57	7.68	0.41	0.49	-0.48	1.36	20.70
49.58	7.77	0.40	0.49	-0.48	1.36	20.66
49.60	7.76	0.40	0.49	-0.48	1.36	20.62
49.62	7.84	0.40	0.48	-0.48	1.36	20.58
49.63	7.94	0.40	0.48	-0.48	1.36	20.56
49.65	7.99	0.40	0.48	-0.48	1.36	20.53
49.67	7.96	0.40	0.48	-0.49	1.36	20.49
49.68	7.90	0.40	0.48	-0.49	1.36	20.45
49.70	7.81	0.40	0.47	-0.49	1.36	20.41
49.72	7.79	0.40	0.47	-0.49	1.36	20.38
49.73	7.82	0.40	0.47	-0.49	1.36	20.35
49.75	7.81	0.40	0.46	-0.49	1.36	20.31
49.77	7.81	0.40	0.46	-0.49	1.36	20.28
49.78	7.87	0.40	0.46	-0.49	1.36	20.25
49.80	7.93	0.39	0.46	-0.49	1.36	20.21
49.82	8.01	0.39	0.46	-0.49	1.36	20.17
49.83	8.08	0.39	0.45	-0.49	1.36	20.14
49.85	8.04	0.39	0.45	-0.49	1.36	20.11
49.87	7.99	0.39	0.45	-0.49	1.36	20.07
49.88	7.99	0.39	0.45	-0.50	1.36	20.03
49.90	8.02	0.39	0.44	-0.50	1.36	20.00
49.92	8.05	0.39	0.44	-0.50	1.36	19.96
49.93	8.04	0.39	0.44	-0.49	1.36	19.93
49.95	8.01	0.39	0.44	-0.49	1.36	19.90
49.97	8.02	0.39	0.43	-0.50	1.36	19.87
49.98	7.93	0.39	0.43	-0.50	1.36	19.84
50.00	7.82	0.39	0.43	-0.50	1.36	19.80

50.02	7.93	0.38	0.43	-0.50	1.36	19.77
50.03	8.10	0.38	0.43	-0.50	1.36	19.74
50.05	8.01	0.38	0.42	-0.50	1.36	19.71
50.07	7.76	0.38	0.42	-0.50	1.36	19.67
50.08	7.73	0.38	0.42	-0.50	1.36	19.63
50.10	7.87	0.38	0.42	-0.51	1.36	19.60
50.12	7.90	0.38	0.42	-0.51	1.36	19.57
50.13	7.99	0.38	0.41	-0.51	1.36	19.53
50.15	8.07	0.38	0.41	-0.51	1.36	19.50
50.17	8.05	0.38	0.41	-0.51	1.36	19.47
50.18	8.05	0.38	0.41	-0.51	1.36	19.44
50.20	8.01	0.37	0.40	-0.51	1.36	19.40
50.22	7.88	0.38	0.40	-0.51	1.36	19.36
50.23	7.84	0.38	0.40	-0.51	1.36	19.33
50.25	7.84	0.38	0.40	-0.51	1.36	19.30
50.27	7.82	0.38	0.40	-0.51	1.36	19.27
50.28	7.82	0.38	0.39	-0.51	1.36	19.23
50.30	7.85	0.38	0.39	-0.51	1.36	19.20
50.32	7.82	0.38	0.39	-0.51	1.36	19.17
50.33	7.88	0.37	0.39	-0.51	1.36	19.14
50.35	7.98	0.38	0.39	-0.51	1.36	19.11
50.37	7.91	0.38	0.38	-0.52	1.36	19.09
50.38	7.77	0.37	0.38	-0.52	1.36	19.05
50.40	7.67	0.37	0.38	-0.52	1.36	19.03
50.42	7.73	0.37	0.38	-0.52	1.36	18.99
50.43	7.88	0.37	0.38	-0.52	1.36	18.96
50.45	7.93	0.37	0.37	-0.52	1.36	18.93
50.47	7.85	0.37	0.37	-0.52	1.36	18.89
50.48	7.79	0.37	0.37	-0.52	1.36	18.85
50.50	7.82	0.37	0.37	-0.52	1.36	18.82
50.52	7.90	0.37	0.37	-0.52	1.36	18.79
50.53	7.94	0.37	0.37	-0.52	1.36	18.76
50.55	8.02	0.37	0.37	-0.52	1.36	18.74
50.57	8.04	0.37	0.36	-0.52	1.36	18.71
50.58	7.93	0.37	0.36	-0.52	1.36	18.67
50.60	7.77	0.37	0.36	-0.53	1.36	18.64
50.62	7.74	0.37	0.36	-0.53	1.36	18.61
50.63	7.73	0.37	0.36	-0.53	1.36	18.58
50.65	7.62	0.37	0.35	-0.53	1.36	18.55
50.67	7.60	0.37	0.35	-0.53	1.36	18.51
50.68	7.60	0.37	0.35	-0.53	1.36	18.48
50.70	7.67	0.37	0.35	-0.53	1.36	18.44
50.72	7.81	0.37	0.35	-0.53	1.36	18.41
50.73	7.84	0.37	0.35	-0.53	1.36	18.39
50.75	7.82	0.36	0.34	-0.53	1.36	18.37
50.77	7.88	0.36	0.34	-0.53	1.36	18.34
50.78	7.94	0.36	0.34	-0.53	1.36	18.30
50.80	7.99	0.36	0.34	-0.53	1.36	18.26
50.82	7.99	0.36	0.33	-0.53	1.36	18.23
50.83	7.96	0.36	0.33	-0.54	1.36	18.21
50.85	8.02	0.37	0.33	-0.54	1.36	18.18
50.87	8.04	0.36	0.33	-0.54	1.36	18.15
50.88	7.85	0.36	0.33	-0.54	1.36	18.11
50.90	7.79	0.36	0.33	-0.54	1.36	18.08
50.92	7.96	0.36	0.33	-0.54	1.36	18.05
50.93	7.99	0.36	0.32	-0.54	1.36	18.03
50.95	7.87	0.36	0.32	-0.54	1.36	18.00
50.97	7.76	0.36	0.32	-0.54	1.36	17.97
50.98	7.81	0.36	0.32	-0.54	1.36	17.94
51.00	7.94	0.36	0.32	-0.54	1.36	17.91
51.02	7.99	0.36	0.32	-0.55	1.36	17.88
51.03	7.91	0.36	0.31	-0.54	1.36	17.85
51.05	7.93	0.36	0.31	-0.54	1.36	17.82
51.07	8.01	0.36	0.31	-0.55	1.36	17.78
51.08	8.02	0.36	0.31	-0.55	1.36	17.76
51.10	8.01	0.35	0.31	-0.55	1.36	17.73
51.12	7.93	0.35	0.31	-0.55	1.36	17.71
51.13	7.88	0.36	0.31	-0.55	1.36	17.68
51.15	7.81	0.36	0.31	-0.55	1.36	17.65

51.17	7.64	0.36	0.30	-0.55	1.36	17.62
51.18	7.56	0.36	0.30	-0.55	1.36	17.59
51.20	7.56	0.36	0.30	-0.55	1.36	17.56
51.22	7.60	0.36	0.30	-0.55	1.36	17.54
51.23	7.68	0.35	0.30	-0.55	1.36	17.51
51.25	7.59	0.35	0.30	-0.55	1.36	17.48
51.27	7.60	0.35	0.30	-0.56	1.36	17.45
51.28	7.90	0.35	0.29	-0.55	1.36	17.42
51.30	8.13	0.36	0.29	-0.55	1.36	17.39
51.32	7.99	0.36	0.29	-0.55	1.36	17.36
51.33	7.81	0.35	0.29	-0.55	1.36	17.33
51.35	7.90	0.35	0.29	-0.55	1.36	17.31
51.37	7.93	0.36	0.29	-0.56	1.36	17.28
51.38	7.87	0.36	0.28	-0.56	1.36	17.25
51.40	7.79	0.35	0.28	-0.56	1.36	17.22
51.42	7.74	0.35	0.28	-0.56	1.36	17.19
51.43	7.79	0.35	0.28	-0.56	1.36	17.15
51.45	7.84	0.35	0.28	-0.56	1.36	17.13
51.47	7.87	0.35	0.28	-0.56	1.36	17.11
51.48	7.88	0.35	0.28	-0.56	1.36	17.09
51.50	7.85	0.35	0.28	-0.56	1.36	17.06
51.52	7.88	0.35	0.28	-0.55	1.36	17.03
51.53	7.96	0.35	0.28	-0.55	1.36	17.00
51.55	7.96	0.35	0.28	-0.56	1.36	16.98
51.57	7.93	0.35	0.27	-0.56	1.36	16.96
51.58	7.90	0.35	0.27	-0.56	1.36	16.92
51.60	7.96	0.35	0.27	-0.56	1.36	16.88
51.62	7.93	0.35	0.27	-0.55	1.36	16.86
51.63	7.74	0.35	0.27	-0.55	1.36	16.83
51.65	7.71	0.35	0.27	-0.55	1.36	16.81
51.67	7.70	0.35	0.27	-0.56	1.36	16.79
51.68	7.62	0.35	0.26	-0.56	1.36	16.76
51.70	7.70	0.35	0.26	-0.56	1.36	16.72
51.72	7.84	0.35	0.26	-0.56	1.36	16.69
51.73	7.88	0.35	0.26	-0.56	1.36	16.66
51.75	7.93	0.35	0.26	-0.56	1.36	16.64
51.77	7.96	0.35	0.26	-0.56	1.36	16.62
51.78	7.90	0.35	0.26	-0.56	1.36	16.59
51.80	7.96	0.35	0.26	-0.56	1.36	16.56
51.82	8.07	0.35	0.26	-0.56	1.36	16.53
51.83	8.04	0.35	0.25	-0.56	1.36	16.51
51.85	7.96	0.35	0.25	-0.56	1.36	16.48
51.87	7.90	0.35	0.25	-0.56	1.36	16.46
51.88	7.85	0.35	0.25	-0.56	1.36	16.43
51.90	7.87	0.35	0.25	-0.56	1.36	16.40
51.92	7.85	0.35	0.25	-0.56	1.36	16.37
51.93	7.79	0.35	0.25	-0.56	1.36	16.34
51.95	7.70	0.35	0.25	-0.56	1.36	16.33
51.97	7.65	0.35	0.25	-0.56	1.36	16.31
51.98	7.70	0.35	0.25	-0.56	1.36	16.28
52.00	7.77	0.35	0.25	-0.56	1.36	16.24
52.02	7.85	0.35	0.24	-0.56	1.36	16.21
52.03	7.94	0.35	0.24	-0.56	1.36	16.19
52.05	7.82	0.35	0.24	-0.56	1.36	16.16
52.07	7.73	0.35	0.24	-0.56	1.36	16.14
52.08	7.77	0.35	0.24	-0.56	1.36	16.11
52.10	7.81	0.35	0.24	-0.57	1.36	16.08
52.12	7.77	0.35	0.24	-0.56	1.36	16.06
52.13	7.76	0.35	0.24	-0.56	1.36	16.04
52.15	7.84	0.35	0.24	-0.56	1.36	16.01
52.17	7.82	0.35	0.24	-0.57	1.36	15.98
52.18	7.87	0.35	0.24	-0.56	1.36	15.96
52.20	8.07	0.35	0.23	-0.57	1.36	15.93
52.22	8.02	0.35	0.23	-0.57	1.36	15.90
52.23	7.84	0.35	0.23	-0.56	1.36	15.88
52.25	7.77	0.35	0.23	-0.56	1.36	15.86
52.27	7.70	0.35	0.23	-0.57	1.36	15.83
52.28	7.68	0.35	0.23	-0.57	1.36	15.80
52.30	7.84	0.35	0.23	-0.57	1.36	15.77

52.32	7.85	0.35	0.23	-0.57	1.36	15.75
52.33	7.77	0.35	0.23	-0.56	1.36	15.72
52.35	7.88	0.35	0.23	-0.56	1.36	15.69
52.37	7.96	0.35	0.23	-0.57	1.36	15.67
52.38	7.91	0.35	0.23	-0.57	1.36	15.65
52.40	7.85	0.35	0.22	-0.57	1.36	15.62
52.42	7.91	0.35	0.22	-0.57	1.36	15.60
52.43	7.94	0.36	0.22	-0.57	1.36	15.57
52.45	7.87	0.35	0.22	-0.57	1.36	15.54
52.47	7.73	0.35	0.22	-0.57	1.36	15.51
52.48	7.73	0.35	0.22	-0.57	1.36	15.48
52.50	7.98	0.35	0.22	-0.57	1.36	15.47
52.52	8.10	0.35	0.22	-0.57	1.36	15.44
52.53	7.88	0.35	0.22	-0.57	1.36	15.41
52.55	7.77	0.35	0.22	-0.57	1.36	15.39
52.57	8.01	0.35	0.22	-0.57	1.36	15.36
52.58	8.15	0.35	0.22	-0.57	1.36	15.34
52.60	8.04	0.35	0.22	-0.57	1.36	15.32
52.62	7.84	0.35	0.22	-0.57	1.36	15.29
52.63	7.76	0.35	0.21	-0.57	1.36	15.27
52.65	7.82	0.35	0.21	-0.57	1.36	15.25
52.67	7.91	0.35	0.21	-0.57	1.36	15.23
52.68	7.91	0.35	0.21	-0.57	1.36	15.19
52.70	7.93	0.35	0.21	-0.57	1.36	15.16
52.72	7.98	0.35	0.21	-0.57	1.36	15.14
52.73	7.96	0.35	0.21	-0.57	1.36	15.12
52.75	7.87	0.36	0.21	-0.57	1.36	15.10
52.77	7.84	0.36	0.21	-0.57	1.36	15.08
52.78	7.90	0.36	0.21	-0.57	1.36	15.06
52.80	7.98	0.35	0.21	-0.57	1.36	15.03
52.82	7.93	0.36	0.21	-0.57	1.36	15.00
52.83	7.85	0.36	0.21	-0.57	1.36	14.97
52.85	7.96	0.36	0.21	-0.57	1.36	14.95
52.87	8.10	0.36	0.21	-0.57	1.36	14.93
52.88	8.10	0.36	0.21	-0.57	1.36	14.90
52.90	8.05	0.36	0.21	-0.57	1.36	14.89
52.92	7.93	0.36	0.20	-0.57	1.36	14.87
52.93	7.79	0.36	0.20	-0.58	1.36	14.84
52.95	7.77	0.36	0.20	-0.58	1.36	14.82
52.97	7.88	0.36	0.20	-0.57	1.36	14.79
52.98	7.98	0.36	0.20	-0.57	1.36	14.77
53.00	7.91	0.36	0.20	-0.57	1.36	14.75
53.02	7.91	0.36	0.20	-0.57	1.36	14.72
53.03	7.93	0.36	0.20	-0.57	1.36	14.69
53.05	7.90	0.36	0.20	-0.57	1.36	14.67
53.07	7.96	0.36	0.20	-0.57	1.36	14.65
53.08	7.94	0.36	0.20	-0.57	1.36	14.63
53.10	7.91	0.36	0.20	-0.57	1.36	14.61
53.12	7.94	0.36	0.20	-0.58	1.36	14.58
53.13	7.91	0.36	0.19	-0.58	1.36	14.55
53.15	7.90	0.36	0.19	-0.58	1.36	14.53
53.17	7.82	0.36	0.19	-0.58	1.36	14.51
53.18	7.79	0.37	0.19	-0.58	1.36	14.49
53.20	7.88	0.37	0.19	-0.58	1.36	14.47
53.22	7.98	0.37	0.19	-0.58	1.36	14.45
53.23	8.01	0.37	0.19	-0.58	1.36	14.42
53.25	7.88	0.37	0.19	-0.58	1.36	14.39
53.27	7.84	0.37	0.19	-0.58	1.36	14.36
53.28	7.96	0.37	0.19	-0.58	1.36	14.34
53.30	8.04	0.37	0.19	-0.58	1.36	14.33
53.32	7.94	0.37	0.19	-0.58	1.36	14.31
53.33	7.94	0.37	0.19	-0.58	1.36	14.29
53.35	7.91	0.37	0.19	-0.58	1.36	14.27
53.37	7.81	0.37	0.19	-0.58	1.36	14.25
53.38	7.87	0.37	0.19	-0.58	1.36	14.22
53.40	8.04	0.37	0.19	-0.58	1.36	14.19
53.42	8.02	0.38	0.19	-0.58	1.36	14.17
53.43	7.98	0.38	0.19	-0.58	1.36	14.15
53.45	8.02	0.38	0.18	-0.58	1.36	14.12

53.47	8.13	0.38	0.18	-0.58	1.36	14.10
53.48	8.19	0.38	0.18	-0.58	1.36	14.08
53.50	8.15	0.38	0.18	-0.58	1.36	14.06
53.52	8.01	0.38	0.18	-0.58	1.36	14.04
53.53	7.98	0.38	0.18	-0.58	1.36	14.02
53.55	7.99	0.38	0.18	-0.59	1.36	14.00
53.57	8.01	0.38	0.18	-0.59	1.36	13.97
53.58	8.04	0.38	0.18	-0.58	1.36	13.94
53.60	8.07	0.38	0.18	-0.58	1.36	13.92
53.62	7.96	0.38	0.18	-0.58	1.36	13.90
53.63	7.76	0.38	0.18	-0.58	1.36	13.88
53.65	7.82	0.38	0.18	-0.58	1.36	13.86
53.67	7.99	0.38	0.18	-0.58	1.36	13.83
53.68	8.05	0.38	0.18	-0.58	1.36	13.81
53.70	8.05	0.38	0.18	-0.58	1.36	13.79
53.72	7.94	0.39	0.17	-0.58	1.36	13.78
53.73	7.94	0.39	0.17	-0.58	1.36	13.75
53.75	8.01	0.39	0.17	-0.58	1.36	13.73
53.77	8.04	0.39	0.18	-0.58	1.36	13.71
53.78	8.01	0.39	0.18	-0.58	1.36	13.69
53.80	7.91	0.39	0.17	-0.58	1.36	13.66
53.82	7.87	0.39	0.17	-0.58	1.36	13.64
53.83	7.94	0.39	0.17	-0.59	1.36	13.62
53.85	7.96	0.39	0.17	-0.59	1.36	13.59
53.87	7.90	0.39	0.17	-0.59	1.36	13.58
53.88	7.82	0.40	0.17	-0.59	1.36	13.56
53.90	7.71	0.39	0.17	-0.58	1.36	13.54
53.92	7.73	0.39	0.17	-0.59	1.36	13.52
53.93	7.93	0.39	0.17	-0.59	1.36	13.50
53.95	8.07	0.39	0.17	-0.58	1.36	13.47
53.97	8.02	0.39	0.17	-0.58	1.36	13.45
53.98	7.88	0.39	0.17	-0.58	1.36	13.43
54.00	7.94	0.39	0.17	-0.58	1.36	13.41
54.02	8.02	0.39	0.16	-0.58	1.36	13.39
54.03	7.93	0.39	0.16	-0.58	1.36	13.37
54.05	7.91	0.39	0.17	-0.58	1.36	13.36
54.07	7.96	0.39	0.16	-0.59	1.36	13.34
54.08	7.93	0.40	0.16	-0.59	1.36	13.31
54.10	7.88	0.40	0.16	-0.59	1.36	13.29
54.12	7.87	0.40	0.16	-0.59	1.36	13.27
54.13	7.88	0.39	0.16	-0.59	1.36	13.25
54.15	8.02	0.39	0.16	-0.59	1.36	13.23
54.17	8.10	0.39	0.16	-0.59	1.36	13.20
54.18	8.02	0.40	0.16	-0.59	1.36	13.17
54.20	7.98	0.40	0.16	-0.59	1.36	13.15
54.22	7.93	0.39	0.16	-0.59	1.36	13.13
54.23	7.93	0.39	0.16	-0.59	1.36	13.11
54.25	7.90	0.39	0.16	-0.59	1.36	13.10
54.27	7.91	0.39	0.16	-0.59	1.36	13.08
54.28	7.90	0.40	0.16	-0.59	1.36	13.06
54.30	7.79	0.40	0.16	-0.59	1.36	13.04
54.32	7.77	0.40	0.16	-0.59	1.36	13.02
54.33	7.79	0.39	0.16	-0.59	1.36	13.00
54.35	7.77	0.39	0.16	-0.59	1.36	12.98
54.37	7.71	0.39	0.16	-0.59	1.36	12.95
54.38	7.64	0.39	0.16	-0.59	1.36	12.93
54.40	7.68	0.39	0.16	-0.59	1.36	12.92
54.42	7.81	0.39	0.16	-0.59	1.36	12.90
54.43	7.93	0.39	0.16	-0.59	1.36	12.87
54.45	7.90	0.39	0.16	-0.59	1.36	12.85
54.47	7.85	0.39	0.16	-0.59	1.36	12.84
54.48	7.81	0.39	0.16	-0.59	1.36	12.82
54.50	7.74	0.39	0.16	-0.59	1.36	12.80
54.52	7.77	0.39	0.15	-0.59	1.36	12.77
54.53	7.74	0.39	0.15	-0.59	1.36	12.75
54.55	7.71	0.39	0.15	-0.59	1.36	12.73
54.57	7.77	0.39	0.15	-0.59	1.36	12.72
54.58	7.87	0.39	0.15	-0.59	1.36	12.70
54.60	7.99	0.38	0.16	-0.58	1.36	12.68

54.62	8.07	0.39	0.16	-0.59	1.36	12.66
54.63	7.94	0.39	0.16	-0.59	1.36	12.64
54.65	7.84	0.38	0.16	-0.59	1.36	12.62
54.67	7.99	0.38	0.15	-0.58	1.36	12.61
54.68	8.01	0.38	0.15	-0.58	1.36	12.58
54.70	7.85	0.38	0.15	-0.58	1.36	12.56
54.72	7.76	0.39	0.15	-0.58	1.36	12.53
54.73	7.73	0.39	0.15	-0.59	1.36	12.52
54.75	7.73	0.38	0.15	-0.59	1.36	12.50
54.77	7.77	0.38	0.15	-0.59	1.36	12.47
54.78	7.87	0.37	0.15	-0.59	1.36	12.45
54.80	7.81	0.37	0.15	-0.59	1.36	12.43
54.82	7.71	0.38	0.15	-0.59	1.36	12.41
54.83	7.65	0.38	0.15	-0.59	1.36	12.39
54.85	7.68	0.38	0.15	-0.59	1.36	12.37
54.87	7.71	0.38	0.15	-0.58	1.36	12.36
54.88	7.76	0.37	0.15	-0.58	1.36	12.34
54.90	7.81	0.37	0.15	-0.58	1.36	12.32
54.92	7.91	0.37	0.15	-0.58	1.36	12.30
54.93	7.90	0.37	0.15	-0.58	1.36	12.29
54.95	7.73	0.37	0.15	-0.58	1.36	12.27
54.97	7.65	0.37	0.15	-0.58	1.36	12.24
54.98	7.62	0.37	0.15	-0.58	1.36	12.22
55.00	7.64	0.36	0.15	-0.58	1.36	12.20
55.02	7.64	0.37	0.15	-0.59	1.36	12.19
55.03	7.62	0.37	0.15	-0.58	1.36	12.17
55.05	7.67	0.36	0.15	-0.58	1.36	12.15
55.07	7.65	0.36	0.15	-0.59	1.36	12.12
55.08	7.50	0.36	0.15	-0.58	1.36	12.10
55.10	7.31	0.36	0.15	-0.58	1.36	12.08
55.12	7.30	0.36	0.15	-0.58	1.36	12.06
55.13	7.48	0.36	0.15	-0.58	1.36	12.05
55.15	7.60	0.36	0.15	-0.58	1.36	12.03
55.17	7.60	0.36	0.15	-0.58	1.36	12.01
55.18	7.74	0.36	0.15	-0.58	1.36	11.99
55.20	7.81	0.36	0.14	-0.58	1.36	11.97
55.22	7.82	0.35	0.15	-0.58	1.36	11.95
55.23	7.84	0.35	0.15	-0.58	1.36	11.94
55.25	7.76	0.35	0.15	-0.58	1.36	11.92
55.27	7.70	0.35	0.15	-0.58	1.36	11.91
55.28	7.73	0.35	0.15	-0.58	1.36	11.89
55.30	7.71	0.35	0.14	-0.58	1.36	11.86
55.32	7.74	0.35	0.14	-0.58	1.36	11.84
55.33	7.81	0.35	0.14	-0.58	1.36	11.82
55.35	7.76	0.35	0.14	-0.58	1.36	11.81
55.37	7.68	0.35	0.14	-0.58	1.36	11.79
55.38	7.70	0.35	0.14	-0.58	1.36	11.77
55.40	7.77	0.34	0.14	-0.58	1.36	11.75
55.42	7.81	0.34	0.14	-0.58	1.36	11.73
55.43	7.87	0.34	0.14	-0.58	1.36	11.71
55.45	7.88	0.34	0.14	-0.58	1.36	11.70
55.47	7.85	0.35	0.14	-0.58	1.36	11.68
55.48	7.81	0.34	0.14	-0.58	1.36	11.66
55.50	7.74	0.34	0.14	-0.58	1.36	11.64
55.52	7.68	0.34	0.14	-0.58	1.36	11.62
55.53	7.62	0.34	0.14	-0.58	1.36	11.60
55.55	7.67	0.34	0.14	-0.58	1.36	11.59
55.57	7.71	0.34	0.14	-0.58	1.36	11.57
55.58	7.67	0.34	0.14	-0.58	1.36	11.55
55.60	7.71	0.34	0.14	-0.58	1.36	11.54
55.62	7.81	0.34	0.14	-0.58	1.36	11.52
55.63	7.79	0.34	0.14	-0.58	1.36	11.50
55.65	7.74	0.34	0.14	-0.58	1.36	11.48
55.67	7.68	0.34	0.14	-0.58	1.36	11.47
55.68	7.71	0.34	0.14	-0.58	1.36	11.45
55.70	7.67	0.34	0.14	-0.58	1.36	11.43
55.72	7.50	0.34	0.14	-0.58	1.36	11.41
55.73	7.59	0.34	0.14	-0.58	1.36	11.39
55.75	7.76	0.34	0.14	-0.58	1.36	11.37

55.77	7.77	0.33	0.14	-0.58	1.36	11.36
55.78	7.71	0.33	0.14	-0.58	1.36	11.35
55.80	7.76	0.34	0.14	-0.58	1.36	11.32
55.82	7.73	0.34	0.14	-0.58	1.36	11.30
55.83	7.54	0.33	0.14	-0.58	1.36	11.29
55.85	7.45	0.34	0.14	-0.58	1.36	11.27
55.87	7.54	0.34	0.14	-0.57	1.36	11.25
55.88	7.65	0.34	0.14	-0.57	1.36	11.23
55.90	7.65	0.34	0.14	-0.58	1.36	11.22
55.92	7.60	0.34	0.14	-0.58	1.36	11.20
55.93	7.56	0.34	0.13	-0.57	1.36	11.18
55.95	7.64	0.34	0.14	-0.57	1.36	11.16
55.97	7.62	0.33	0.14	-0.57	1.36	11.14
55.98	7.65	0.33	0.13	-0.57	1.36	11.13
56.00	7.85	0.33	0.13	-0.57	1.36	11.11
56.02	7.90	0.33	0.14	-0.57	1.36	11.10
56.03	7.84	0.33	0.14	-0.57	1.36	11.08
56.05	7.79	0.33	0.14	-0.57	1.36	11.06
56.07	7.73	0.33	0.14	-0.57	1.36	11.04
56.08	7.70	0.33	0.14	-0.57	1.36	11.03
56.10	7.70	0.33	0.14	-0.57	1.36	11.01
56.12	7.64	0.33	0.13	-0.57	1.36	11.00
56.13	7.59	0.33	0.13	-0.57	1.36	10.98
56.15	7.59	0.33	0.13	-0.57	1.36	10.96
56.17	7.62	0.33	0.13	-0.57	1.36	10.95
56.18	7.77	0.33	0.13	-0.57	1.36	10.93
56.20	7.87	0.33	0.13	-0.57	1.36	10.91
56.22	7.82	0.34	0.14	-0.57	1.36	10.90
56.23	7.74	0.34	0.14	-0.57	1.36	10.89
56.25	7.73	0.34	0.14	-0.56	1.36	10.87
56.27	7.74	0.33	0.13	-0.57	1.36	10.85
56.28	7.74	0.33	0.13	-0.57	1.36	10.84
56.30	7.74	0.33	0.13	-0.56	1.36	10.81
56.32	7.74	0.34	0.13	-0.56	1.36	10.79
56.33	7.81	0.34	0.13	-0.57	1.36	10.77
56.35	7.81	0.33	0.13	-0.57	1.36	10.76
56.37	7.74	0.34	0.13	-0.56	1.36	10.75
56.38	7.73	0.33	0.13	-0.56	1.36	10.73
56.40	7.81	0.33	0.13	-0.56	1.36	10.71
56.42	7.91	0.33	0.13	-0.56	1.36	10.70
56.43	7.96	0.34	0.13	-0.56	1.36	10.69
56.45	7.93	0.33	0.13	-0.57	1.36	10.66
56.47	7.94	0.33	0.13	-0.57	1.36	10.64
56.48	8.01	0.33	0.13	-0.56	1.36	10.63
56.50	8.01	0.33	0.14	-0.56	1.36	10.61
56.52	7.96	0.33	0.14	-0.57	1.36	10.59
56.53	7.93	0.34	0.13	-0.56	1.36	10.58
56.55	7.99	0.34	0.13	-0.56	1.36	10.57
56.57	8.04	0.34	0.13	-0.56	1.36	10.55
56.58	7.90	0.34	0.13	-0.56	1.36	10.53
56.60	7.70	0.34	0.13	-0.56	1.36	10.51
56.62	7.68	0.34	0.13	-0.57	1.36	10.49
56.63	7.76	0.34	0.13	-0.56	1.36	10.48
56.65	7.82	0.34	0.13	-0.56	1.36	10.47
56.67	7.87	0.34	0.13	-0.56	1.36	10.45
56.68	7.91	0.34	0.13	-0.56	1.36	10.43
56.70	7.93	0.33	0.13	-0.56	1.36	10.42
56.72	7.91	0.33	0.13	-0.56	1.36	10.40
56.73	7.93	0.34	0.13	-0.56	1.36	10.38
56.75	7.98	0.34	0.13	-0.56	1.36	10.36
56.77	7.98	0.34	0.13	-0.56	1.36	10.35
56.78	8.04	0.34	0.13	-0.56	1.36	10.33
56.80	8.01	0.34	0.13	-0.56	1.36	10.32
56.82	7.84	0.34	0.13	-0.56	1.36	10.30
56.83	7.82	0.34	0.13	-0.56	1.36	10.28
56.85	8.01	0.34	0.13	-0.56	1.36	10.27
56.87	8.05	0.34	0.13	-0.56	1.36	10.25
56.88	7.94	0.34	0.13	-0.56	1.36	10.24
56.90	7.91	0.34	0.13	-0.56	1.36	10.23

56.92	7.94	0.33	0.13	-0.56	1.36	10.22
56.93	7.93	0.33	0.13	-0.56	1.36	10.20
56.95	7.90	0.33	0.13	-0.56	1.36	10.17
56.97	7.94	0.34	0.13	-0.56	1.36	10.15
56.98	7.94	0.34	0.13	-0.55	1.36	10.14
57.00	7.91	0.34	0.13	-0.55	1.36	10.12
57.02	7.84	0.34	0.13	-0.55	1.36	10.11
57.03	7.84	0.33	0.13	-0.55	1.36	10.09
57.05	7.94	0.33	0.13	-0.55	1.36	10.07
57.07	7.96	0.33	0.13	-0.55	1.36	10.06
57.08	8.02	0.33	0.14	-0.55	1.36	10.05
57.10	8.10	0.34	0.13	-0.55	1.36	10.04
57.12	8.07	0.34	0.14	-0.55	1.36	10.01
57.13	7.91	0.33	0.14	-0.55	1.36	10.00
57.15	7.98	0.33	0.14	-0.55	1.36	9.98
57.17	8.05	0.33	0.14	-0.55	1.36	9.97
57.18	7.96	0.34	0.13	-0.55	1.36	9.95
57.20	7.94	0.34	0.13	-0.55	1.36	9.94
57.22	8.05	0.34	0.14	-0.55	1.36	9.92
57.23	8.04	0.33	0.14	-0.54	1.36	9.90
57.25	7.84	0.33	0.13	-0.54	1.36	9.89
57.27	7.82	0.33	0.13	-0.54	1.36	9.87
57.28	7.99	0.33	0.14	-0.54	1.36	9.86
57.30	7.96	0.34	0.14	-0.54	1.36	9.85
57.32	7.90	0.34	0.14	-0.54	1.36	9.83
57.33	7.91	0.34	0.14	-0.54	1.36	9.81
57.35	7.98	0.34	0.14	-0.54	1.36	9.80
57.37	8.04	0.33	0.14	-0.54	1.36	9.79
57.38	7.99	0.34	0.14	-0.54	1.36	9.77
57.40	7.93	0.34	0.14	-0.54	1.36	9.75
57.42	7.93	0.33	0.14	-0.54	1.36	9.74
57.43	7.91	0.33	0.14	-0.53	1.36	9.72
57.45	8.04	0.33	0.14	-0.53	1.36	9.70
57.47	8.11	0.33	0.14	-0.53	1.36	9.68
57.48	8.02	0.33	0.14	-0.52	1.36	9.67
57.50	8.05	0.33	0.14	-0.52	1.36	9.66
57.52	8.22	0.33	0.14	-0.52	1.36	9.64
57.53	8.19	0.33	0.14	-0.51	1.36	9.62
57.55	8.08	0.33	0.14	-0.51	1.36	9.61
57.57	8.05	0.33	0.14	-0.51	1.36	9.60
57.58	8.08	0.33	0.14	-0.50	1.36	9.58
57.60	8.05	0.33	0.14	-0.50	1.36	9.57
57.62	7.94	0.33	0.14	-0.50	1.36	9.55
57.63	7.81	0.33	0.14	-0.50	1.36	9.52
57.65	7.79	0.33	0.14	-0.50	1.36	9.51
57.67	7.87	0.33	0.14	-0.50	1.36	9.50
57.68	7.91	0.33	0.14	-0.49	1.36	9.49
57.70	7.91	0.33	0.14	-0.48	1.36	9.47
57.72	7.84	0.33	0.14	-0.48	1.36	9.45
57.73	7.79	0.33	0.14	-0.48	1.36	9.43
57.75	7.84	0.33	0.14	-0.48	1.36	9.43
57.77	7.79	0.33	0.14	-0.47	1.36	9.41
57.78	7.67	0.33	0.14	-0.47	1.36	9.39
57.80	7.79	0.33	0.14	-0.47	1.36	9.38
57.82	7.94	0.33	0.14	-0.46	1.36	9.37
57.83	7.96	0.33	0.14	-0.46	1.36	9.35
57.85	7.90	0.33	0.14	-0.46	1.36	9.33
57.87	7.91	0.33	0.14	-0.45	1.36	9.32
57.88	7.99	0.33	0.14	-0.45	1.36	9.31
57.90	7.99	0.33	0.14	-0.45	1.36	9.29
57.92	7.96	0.33	0.14	-0.45	1.36	9.27
57.93	7.90	0.33	0.15	-0.44	1.36	9.26
57.95	7.79	0.32	0.15	-0.44	1.36	9.24
57.97	7.76	0.32	0.15	-0.44	1.36	9.22
57.98	7.81	0.32	0.15	-0.43	1.36	9.20
58.00	7.76	0.33	0.15	-0.43	1.36	9.19
58.02	7.71	0.33	0.14	-0.43	1.36	9.17
58.03	7.74	0.33	0.15	-0.42	1.36	9.16
58.05	7.71	0.33	0.15	-0.42	1.36	9.15

biomedicines MDPI

58.07	7.70	0.32	0.15	-0.42	1.36	9.13
58.08	7.81	0.32	0.15	-0.42	1.36	9.12
58.10	7.84	0.32	0.15	-0.41	1.36	9.10
58.12	7.91	0.32	0.15	-0.41	1.36	9.09
58.13	7.96	0.32	0.15	-0.41	1.36	9.07
58.15	7.88	0.32	0.15	-0.41	1.36	9.05
58.17	7.87	0.32	0.15	-0.41	1.36	9.04
58.18	7.84	0.32	0.15	-0.41	1.36	9.02
58.20	7.82	0.32	0.15	-0.41	1.36	9.01
58.22	7.87	0.32	0.15	-0.40	1.36	8.99
58.23	7.91	0.32	0.15	-0.40	1.36	8.98
58.25	7.91	0.32	0.15	-0.40	1.36	8.96
58.27	7.82	0.32	0.15	-0.40	1.36	8.95
58.28	7.74	0.32	0.15	-0.40	1.36	8.93
58.30	7.71	0.32	0.15	-0.40	1.36	8.91
58.32	7.70	0.32	0.15	-0.40	1.36	8.90
58.33	7.87	0.32	0.15	-0.40	1.36	8.89
58.35	8.04	0.32	0.15	-0.40	1.36	8.87
58.37	8.01	0.31	0.15	-0.40	1.36	8.85
58.38	7.93	0.32	0.15	-0.40	1.36	8.84
58.40	7.91	0.32	0.15	-0.39	1.36	8.82
58.42	7.85	0.32	0.15	-0.40	1.36	8.82
58.43	7.84	0.32	0.15	-0.40	1.36	8.81
58.45	7.93	0.32	0.15	-0.40	1.36	8.79
58.47	7.96	0.32	0.15	-0.40	1.36	8.78
58.48	7.91	0.32	0.15	-0.40	1.36	8.76
58.50	7.91	0.32	0.15	-0.40	1.36	8.74
58.52	7.90	0.32	0.15	-0.40	1.36	8.72
58.53	7.85	0.32	0.15	-0.40	1.36	8.71
58.55	7.76	0.32	0.15	-0.40	1.36	8.70
58.57	7.71	0.32	0.15	-0.41	1.36	8.69
58.58	7.74	0.32	0.15	-0.41	1.36	8.68
58.60	7.73	0.32	0.15	-0.40	1.36	8.66
58.62	7.76	0.32	0.15	-0.41	1.36	8.64
58.63	7.82	0.32	0.15	-0.41	1.36	8.63
58.65	7.84	0.32	0.15	-0.41	1.36	8.62
58.67	7.90	0.32	0.15	-0.41	1.36	8.61
58.68	7.90	0.32	0.15	-0.41	1.36	8.59
58.70	7.76	0.32	0.15	-0.42	1.36	8.57
58.72	7.71	0.32	0.15	-0.42	1.36	8.56
58.73	7.88	0.31	0.15	-0.42	1.36	8.55
58.75	7.91	0.31	0.15	-0.42	1.36	8.53
58.77	7.76	0.31	0.15	-0.42	1.36	8.51
58.78	7.64	0.31	0.15	-0.43	1.36	8.50
58.80	7.68	0.31	0.15	-0.43	1.36	8.49
58.82	7.81	0.31	0.15	-0.43	1.36	8.48
58.83	7.90	0.31	0.15	-0.44	1.36	8.46
58.85	7.85	0.31	0.15	-0.44	1.36	8.44
58.87	7.82	0.31	0.15	-0.44	1.36	8.43
58.88	7.85	0.31	0.15	-0.44	1.36	8.42
58.90	7.88	0.31	0.15	-0.44	1.36	8.40
58.92	7.87	0.31	0.15	-0.45	1.36	8.39
58.93	7.84	0.31	0.15	-0.45	1.36	8.38
58.95	7.87	0.31	0.15	-0.46	1.36	8.37
58.97	7.90	0.31	0.15	-0.46	1.36	8.35
58.98	7.84	0.31	0.15	-0.46	1.36	8.34
59.00	7.81	0.31	0.15	-0.46	1.36	8.33
59.02	7.82	0.31	0.15	-0.46	1.36	8.31
59.03	7.87	0.31	0.15	-0.47	1.36	8.30
59.05	7.90	0.31	0.15	-0.47	1.36	8.28
59.07	7.88	0.32	0.15	-0.47	1.36	8.27
59.08	7.84	0.32	0.15	-0.47	1.36	8.26
59.10	7.79	0.31	0.15	-0.48	1.36	8.24
59.12	7.74	0.31	0.15	-0.48	1.36	8.23
59.13	7.77	0.31	0.15	-0.48	1.36	8.22
59.15	7.81	0.31	0.15	-0.49	1.36	8.21
59.17	7.82	0.31	0.15	-0.49	1.36	8.19
59.18	7.81	0.31	0.15	-0.49	1.36	8.17
59.20	7.71	0.31	0.15	-0.49	1.36	8.16

59.22	7.68	0.31	0.15	-0.50	1.36	8.14
59.23	7.65	0.31	0.15	-0.50	1.36	8.13
59.25	7.68	0.31	0.15	-0.50	1.36	8.11
59.27	7.79	0.31	0.15	-0.50	1.36	8.10
59.28	7.85	0.31	0.15	-0.50	1.36	8.09
59.30	7.96	0.31	0.15	-0.51	1.36	8.07
59.32	7.91	0.31	0.15	-0.51	1.36	8.06
59.33	7.73	0.31	0.15	-0.51	1.36	8.05
59.35	7.64	0.31	0.15	-0.51	1.36	8.04
59.37	7.76	0.31	0.15	-0.51	1.36	8.02
59.38	7.91	0.31	0.16	-0.51	1.36	8.00
59.40	7.82	0.31	0.16	-0.52	1.36	8.00
59.42	7.84	0.31	0.15	-0.52	1.36	7.99
59.43	7.82	0.31	0.15	-0.52	1.36	7.97
59.45	7.77	0.31	0.16	-0.52	1.36	7.96
59.47	7.82	0.31	0.16	-0.52	1.36	7.94
59.48	7.73	0.31	0.16	-0.52	1.36	7.93
59.50	7.62	0.31	0.16	-0.53	1.36	7.91
59.52	7.73	0.30	0.16	-0.53	1.36	7.90
59.53	7.85	0.31	0.16	-0.53	1.36	7.89
59.55	7.77	0.31	0.16	-0.53	1.36	7.88
59.57	7.65	0.31	0.16	-0.54	1.36	7.86
59.58	7.73	0.30	0.16	-0.53	1.36	7.85
59.60	7.94	0.31	0.17	-0.54	1.36	7.84
59.62	7.96	0.30	0.17	-0.54	1.36	7.83
59.63	7.82	0.30	0.17	-0.54	1.36	7.81
59.65	7.77	0.30	0.17	-0.54	1.36	7.80
59.67	7.74	0.30	0.17	-0.54	1.36	7.78
59.68	7.74	0.30	0.17	-0.54	1.36	7.77
59.70	7.81	0.30	0.17	-0.54	1.36	7.76
59.72	7.88	0.30	0.17	-0.55	1.36	7.74
59.73	7.93	0.30	0.18	-0.55	1.36	7.72
59.75	7.94	0.30	0.18	-0.55	1.36	7.71
59.77	7.85	0.30	0.18	-0.55	1.36	7.70
59.78	7.82	0.30	0.18	-0.55	1.36	7.69
59.80	7.85	0.30	0.18	-0.55	1.36	7.68
59.82	7.85	0.30	0.19	-0.55	1.36	7.67
59.83	7.93	0.31	0.19	-0.56	1.36	7.66
59.85	7.98	0.30	0.19	-0.56	1.36	7.65
59.87	7.82	0.31	0.19	-0.55	1.36	7.63
59.88	7.76	0.31	0.19	-0.56	1.36	7.62
59.90	7.85	0.31	0.19	-0.56	1.36	7.60
59.92	7.84	0.30	0.19	-0.56	1.36	7.59

Supplementary Table 4: Experimental data from Figure 4C. Exosome uptake analysis in regions of interest (dose: 5 µg/ml SCy-MiExo)

Time (min)	Exosome uptake (a.u.)																
0	94	57	58	53	41	86	26	30	60								
30	46	106	68	66	46	73	29	45									
60	59	126	156	85	76	156	94	40									
120	104	179	135	169	157	119	96	51	136	66							
240	113	228	185	168	64	91	82	58	63	47	73	84	121	142			
1440	243	218	206	240	227	233	207	151	163	233	210	155	180	131	202	130	121

Supplementary Table 5: Experimental data from Figure 4C. Exosome uptake analysis in regions of interest (dose: 0.5 µg/ml SCy-MiExo)

Time (min)	Exosome uptake (a.u.)											
0	40	49	38	9	12	10	21	8				
30	115	41	47	52	16	31	31	33	39	138	73	33
60	75	170	105	68	90	60	53	77	28	67	40	
120	126	96	70	115	63	72	42	83	53	66		
240	194	175	136	134	106	118	139	118	141	101		
1440	130	107	121	93	62	63	45	89	136	109	81	46

Supplementary Table 6: Experimental data from Figure 5B. In vivo average radiant efficiency (%), measured in livers from mice treated with SCy-MiExo

Time (h)	Avg. Radiant efficiency (%)		
1	88.36324	69.16803	83.84992
4	91.02773	84.71996	100
24	93.14845	87.16694	98.09679
